# Expanding the Crosslinking Mass Spectrometry Toolbox With Vinyl Sulfone Crosslinkers

**DOI:** 10.1016/j.mcpro.2025.101315

**Published:** 2025-10-21

**Authors:** Anthony Ciancone, Haitao Wu, Katerina Atallah-Yunes, Kitaik Lee, Chris Sibley, Jesse Spivey, Rolf E. Swenson, John S. Schneekloth, Francis J. O’Reilly

**Affiliations:** 1 Center for Structural Biology, Center for Cancer Research, National Cancer Institute (NCI), Frederick, Maryland, USA; 2 Chemistry and Synthesis Center, National Heart, Lung, and Blood Institute, National Institutes of Health, Bethesda, Maryland, USA; 3 Department of Structural Biology, St. Jude Children’s Research Hospital, Memphis, Tennessee, USA; 4 Chemical Biology Laboratory, National Cancer Institute, Frederick, Maryland, USA

**Keywords:** crosslink, vinyl sulfone, diazirine, mass spectrometry, proteomics, proteasome

## Abstract

Crosslinking mass spectrometry (MS) is a powerful approach for probing protein structures. However, most widely used crosslinkers rely on *N*-hydroxysuccinimide (NHS) esters, restricting reactivity primarily to lysine residues and protein *N*-termini, and rendering them incompatible with many amine-containing buffers (e.g., Tris) and key biochemical cofactors (e.g., ATP). To address these limitations, we introduce two novel vinyl-sulfone-based crosslinkers. Alkyne-BVSC is an enrichable, homobifunctional crosslinker featuring an acid-cleavable alkyne handle for downstream peptide enrichment. VSD is a heterobifunctional crosslinker combining a vinyl sulfone with a diazirine moiety for UV-activated photo-crosslinking. Both reagents are synthetically accessible from inexpensive precursors and retain reactivity in amine-rich biochemical environments. We show that vinyl sulfones react with cysteine, histidine, and lysine residues, thereby expanding crosslinkable residues beyond those accessible to NHS-esters. Moreover, we develop a stub-based post-search filtering strategy that leverages the MS-cleavable nature of vinyl sulfone linkages to improve crosslink identification sensitivity. Together, these advances establish vinyl-sulfone-based crosslinkers as versatile and complementary tools for structural proteomics.

## Highlights

• Vinyl sulfones are introduced as chemical crosslinkers that expand the crosslinkable space to cysteine and histidine residues.
• Synthesis of an enrichable vinyl sulfone crosslinker, Alkyne-BVSC, as well as a photo-activatable vinyl sulfone-diazirine crosslinker, VSD.
• Demonstration of a novel, stub-based, post-search filtering approach to boost the number of crosslinks identified.

## Graphical Abstract

**Figure:**
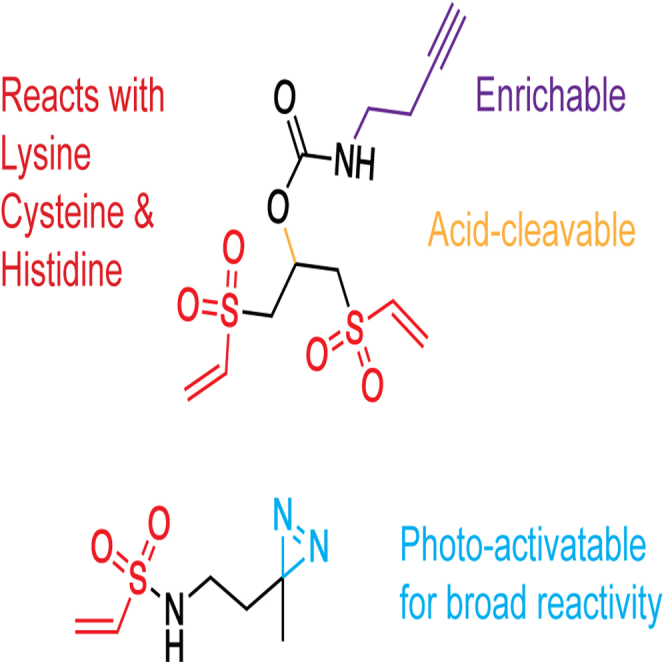

## In Brief

We synthesize novel vinyl sulfone crosslinkers that expand the crosslinkable protein space. We demonstrate that our novel crosslinker, Alkyne-BVSC, can be enriched via its alkyne handle and that this enrichable element can be removed with acid. We elucidate a novel, stub-based, post-search filtering approach using our vinyl sulfone crosslinkers to boost the number of crosslinks. We synthesize a novel, photo-activatable vinyl sulfone crosslinker, VSD, and demonstrate its utility for *in vitro* structural work complementary to traditional NHS-ester-based crosslinkers.

Crosslinking mass spectrometry (crosslinking MS) is a powerful method for uncovering protein–protein interactions and protein structures (1). First, covalent bonds are introduced between spatially proximal residues using bifunctional crosslinkers. Then, these crosslinked residues are discovered through mass spectrometry, providing distance constraints that can inform molecular modeling. The majority of widely used crosslinkers employ *N*-hydroxysuccinimide (NHS) ester moieties as their reactive groups, such as disuccinimidyl sulfoxide (DSSO (2)) or bis(sulfosuccinimidyl) suberate (BS^3^ (3)), which primarily react with primary amines. They have also been incorporated in heterobifunctional crosslinkers, such as succinimidyl 4,4′-azipentanoate (SDA), which combines an NHS-ester on one end of the crosslinker with a diazirine moiety on the other (4). Under physiological conditions, NHS-esters react preferentially with the primary amines present on lysine residues and protein *N*-termini and, to a lesser degree, with the hydroxyl side chains of serine, threonine, and tyrosine (5). Despite their utility, NHS-ester-based crosslinkers have notable limitations. The most important one is the propensity to be quenched by free amines, rendering them incompatible with many common biochemical buffers (e.g., Tris) and cofactors (e.g., ATP). Alternative crosslinker chemistries remain less explored, including maleimides for targeting cysteines (6), sulfonyl-fluorides for targeting nucleophilic residues (7), and both dihydrazide and diazo compounds for targeting aspartate, glutamate, and protein *C*-termini (8, 9).

Vinyl sulfones are a mechanistically distinct class of electrophilic reagents that have shown some promise as chemical probe reagents and have been included in some potential therapeutic drugs (10, 11, 12). Vinyl sulfones have been shown to target cysteine, lysine, and histidine residues on proteins (13, 14, 15). The first reported vinyl-sulfone-based crosslinker, pBVS, demonstrated that the bonds formed with these residues are inherently cleavable during HCD fragmentation in the mass spectrometer (15), which can hinder identification of the crosslinked residues. Still, many questions remain about vinyl-sulfone-based crosslinkers: the optimal fragmentation regime, MS cleavability during database search, incorporation into heterobifunctional crosslinkers, and viability for use in live-cell experiments.

Here, we present a new generation of vinyl-sulfone-based crosslinkers designed to overcome key limitations of existing reagents and describe their properties and applications in crosslinking MS. We introduce two novel compounds: Alkyne-BVSC, a homobifunctional vinyl sulfone crosslinker equipped with an acid-cleavable alkyne handle, enabling flexible strategies for peptide enrichment, and VSD, a heterobifunctional crosslinker that combines a vinyl sulfone and a diazirine moiety for UV-activated photo-crosslinking. Importantly, both crosslinkers can be synthesized from inexpensive, readily available starting materials with simple reaction schema. We demonstrate that these reagents maintain reactivity in amine-rich biochemical environments, including buffers and cofactors that typically quench NHS-esters. In addition, the MS-cleavable nature of the vinyl sulfone linkage enables a novel stub-based, post-search filtering strategy, which we show significantly improves the identification of crosslinked residue-pairs. Together, these features establish vinyl sulfone chemistry as a versatile and orthogonal addition to the crosslinking MS reagent repertoire, expanding both chemical reactivity and experimental flexibility.

## Results and Discussion

### Design of Enrichable, Acid-, and MS-cleavable Vinyl-Sulfone-Based Crosslinkers

**Fig. 1:**
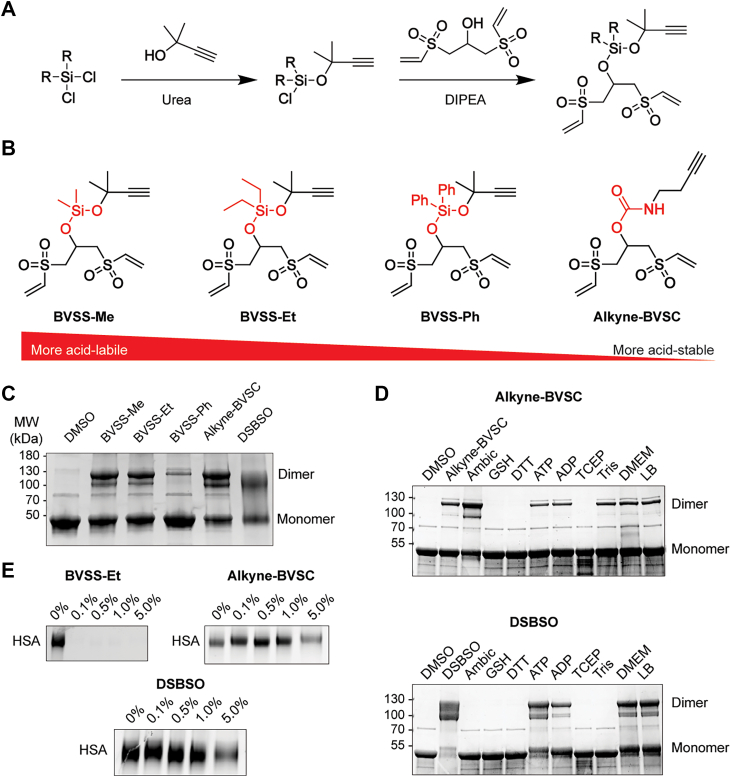
**Vinyl-sulfone-based crosslinkers with acid- and MS-cleavable properties.***A*, general synthetic scheme for the preparation of vinyl-sulfone-based crosslinkers (see methods). *B*, chemical structures of the four novel crosslinkers: BVSS-Me, BVSS-Et, BVSS-Ph, and Alkyne-BVSC. The acid-cleavable moiety in each compound is highlighted in red. Compounds are shown from left to right in order of decreasing acid lability, with BVSS-Me being the most acid-cleavable. *C*, SDS-PAGE analysis of Coomassie-stained α-enolase crosslinked with the indicated reagents (1 mM, 1 h, 37 °C). All four novel crosslinkers, along with DSBSO, promote dimer formation to varying degrees, demonstrating their ability to crosslink protein in solution. The DMSO-treated sample serves as a negative control (*n = 2*). *D*, alkyne-BVSC (*top*) and DSBSO (*bottom*) were pre-incubated with the indicated biochemical additives prior to reaction with α-enolase to assess buffer compatibility. DSBSO crosslinking was quenched by ammonium bicarbonate (ambic), glutathione (GSH), dithiothreitol (DTT), tris(2-carboxyethyl)phosphine (TCEP), and Tris, whereas Alkyne-BVSC retained activity. Comparatively, Alkyne-BVSC was quenched by GSH, DTT, and TCEP (*n = 2*). *E*, representative panel from the TFA-dependent cleavage assay (1 h at room temperature) showing crosslinked HSA samples labeled with rhodamine via CuAAC. BVSS-Et, Alkyne-BVSC, and DSBSO were tested at varying TFA concentrations; water (0%) served as a negative control. Increased TFA concentrations result in cleavage of the rhodamine-labeled crosslinker, as evidenced by reduced fluorescence at the monomeric protein band (*n = 2*)

We synthesized four different homobifunctional, vinyl-sulfone-based crosslinkers, each containing an acid-cleavable moiety comprising an alkyne handle to allow clicking on of biotin for downstream enrichment via streptavidin beads (Fig. 1, *A* and *B*). The syntheses for these compounds are straightforward, and the starting materials are readily available. Our crosslinkers explore several acid-labile motifs that do not require high concentrations of acid exposure that can cause artificial deamidation and formylation of peptides, complicating downstream analysis (16). Three of the crosslinkers contain an acid-cleavable siloxane with variably bulky side groups to modulate acid cleavability (BVSS-Me, BVSS-Et, and BVSS-Ph (Bisvinylsulfonesilane-methyl/ethyl/phenyl)) (Fig. 1*B*). The fourth crosslinker, Alkyne-BVSC (Alkyne-Bisvinylsulfonecarbamate), contains a comparatively more stable carbamate (Fig. 1*B*).

To test whether these new compounds can crosslink proteins, we incubated BVSS-Me, BVSS-Et, BVSS-Ph, Alkyne-BVSC, and Alkyne-A-DSBSO (DSBSO) with alpha-enolase for 1 h at 37 °C. We visualized the formation of an enolase dimer via SDS-PAGE (Fig. 1*C*). We demonstrated that this dimer formation was dose-dependent using BVSS-Et (Supplemental Fig. S1). Critically, we noted that BVSS-Ph was largely insoluble in water, forming an opaque solution, which likely rules it out as a useful crosslinker in biological contexts. Next, we pre-incubated Alkyne-BVSC and DSBSO with common buffers and cofactors before adding these solutions to enolase to crosslink the protein. While DSBSO was unable to crosslink enolase after exposure to tris, ammonium bicarbonate, ATP, ADP, TCEP, glutathione (GSH), and DTT, Alkyne-BVSC was only quenched by the thiol-containing compounds DTT and GSH, along with TCEP (Fig. 1*D*).

To determine whether the alkyne moiety remains accessible for click-chemistry and subsequent acid cleavage after crosslinking, we performed the same gel-based assays except, after crosslinking, we appended on rhodamine using copper-catalyzed azide-alkyne cycloaddition (CuAAC (17)) before separation by SDS-PAGE (18). We then imaged the gel using 565 nm wavelength light to visualize the rhodamine fluorescence on the crosslinked protein, which confirmed fluorescent signal for each crosslinker at the estimated molecular weights for both HSA and enolase (Fig. 1*E*, Supplemental Figs. S2–S4). We tested the acid-lability of our novel crosslinkers by crosslinking HSA, clicking on rhodamine, and treating the crosslinked proteins with varying concentrations of trifluoroacetic acid (TFA) at different time points and temperatures. We performed a similar gel assay to visualize the loss of crosslinked protein signal and consequent build-up of unbound rhodamine at the bottom of the gel as the primary readout. With this gel-based assay, we found that BVSS-Et, and by extension BVSS-Me, are both highly acid-cleavable (Fig. 1*E* and Supplemental Fig. S2). Alkyne-BVSC is comparably more resistant, but appears largely acid-cleaved in 5% TFA after 1 h (37 °C); however, it seems 5% TFA for 24 h (37 °C) is required for complete acid-cleavage (Fig. 1*E* and Supplemental Fig. S3). DSBSO has been noted to require high concentrations of acid for extended periods of time for complete cleavage of the dioxane ring (19, 20); although our gel-based assay agrees with these previously published findings, we find DSBSO requires only 0.5% TFA for 24 h at 37 °C to achieve nearly complete cleavage (Fig. 1*E* and Supplemental Fig. S4).

**Fig. 2:**
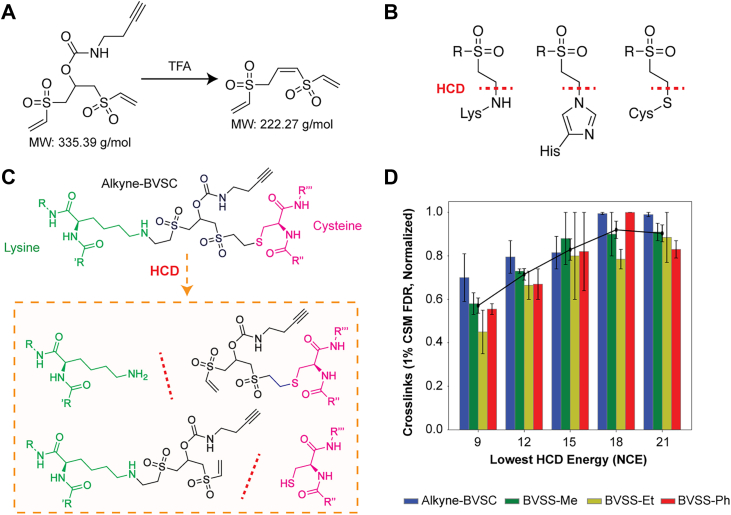
**Vinyl-sulfone-based crosslinkers are highly compatible for crosslinking mass spectrometry.***A*, chemical structures of both Alkyne-BVSC (*left*) and its predicted, TFA-cleaved product (*right*), with molecular weights (MW) listed. *B*, vinyl-sulfones react covalently with lysine, histidine, and cysteine residues. HCD conditions can cleave these bonds via retro-Michael addition (*red* dashes). *C*, an illustration of an example residue-pair crosslink between a lysine (*green*), Alkyne-BVSC (*black*), and a cysteine (*pink*). HCD (*red* dashes) can fragment the vinyl-sulfone-nucleophile bonds as shown (*orange box*), showing liberation of the crosslinked peptides. *D*, Bar plot of crosslinked spectral matches (CSMs) at a 1% FDR at the CSM level for HSA for each crosslinker. A three-step, stepped HCD regime was used to fragment the precursor ions, with the two highest energies fixed at 26 and 30 NCE. Each dataset was normalized to the highest number of crosslinks for that dataset, where the maximum value was set to 1.0 (*n = 2*)

BVSS-Et completely cleaved at all tested TFA concentrations, so we feared that the crosslinker could be prone to unwanted acid-cleavage under even mildly acidic conditions. Comparatively, Alkyne-BVSC demonstrates a controllable acid-cleavability, while still appearing more labile than DSBSO (Fig. 1*E*). Although the gel-based rhodamine assay was not definitive for which acid concentrations are optimal to fully cleave Alkyne-BVSC, we identified a monolink mass of 222.27 Da on peptides from an Alkyne-BVSC crosslinked HSA sample (164.98 Da for cysteine searched with a fixed carbamidomethylation modification) using an Open modification search (21) in FragPipe/MSFragger (22) (Fig. 2*A*, Supplemental Table S1). This modification mass can be explained by removal of the carbamate moiety: a cleavage that occurs when TFA is added to the peptides to achieve a pH of 3 for C18 cleanup. We detect four times the number of peptide spectral matches with the acid-cleaved mass of the crosslinker compared to the uncleaved mass (Supplemental Table S1). Together, these data demonstrate that we can control Alkyne-BVSC’s acid-lability for crosslinking MS applications.

### Optimizing HCD Fragmentation and Data Filtering Strategies for Cinyl Sulfone Crosslinkers

**Fig. 3:**
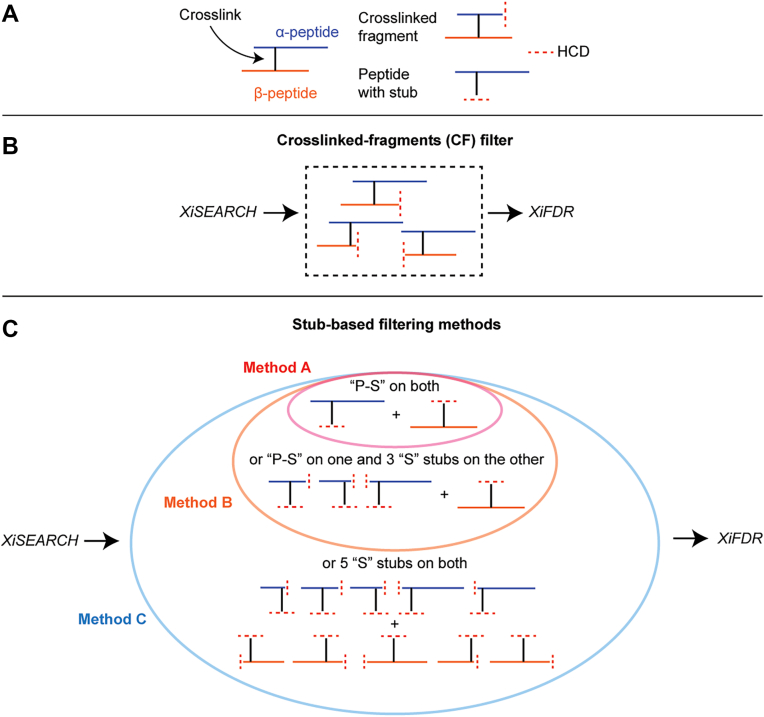
**Post-search filters visualized.***A*, peptides (*red* and *blue lines*) that have been crosslinked (vertical *black* lines) can fragment in the mass spectrometer with HCD (dashed *red* lines), which can result in cleavage of the peptide or the vinyl-sulfone bond. At low-energy HCD, the peptide bond can fragment while the crosslink is maintained, generating a crosslinked-fragment (CF, dashed box). Alternatively, the crosslink itself can fragment, liberating the two peptides and generating a fragment containing the intact peptide and crosslinker (“P-S” stub). There is another possibility: a double fragmentation via HCD of both the crosslink and the crosslinker-containing peptide simultaneously (“S” stub). After xiSEARCH, we can filter CSMs using the CF method (*B*, dashed box), which requires three CF peptides for each peptide, or a stub-based approach (*C*, bubble plots) before assessing FDR. In this paper, we test three different stub-based filtering methods: method A (*red bubble*)—require a P-S on both peptides, method B (*orange bubble*)—method A in addition to allowing for one P-S on one peptide and 3 S stubs on the other peptide, and method C (*blue bubble*)—method B in addition to allowing for 5 S stubs on both peptides. Essentially, method B is a less restrictive form of method A and method C is a less restrictive form of method B. Method C is the selected method we chose for stub-based filtering for this work.

Vinyl-sulfone-derived crosslinks to cysteine, lysine, and histidine can cleave via HCD in the mass spectrometer through a retro-Michael addition to liberate the crosslinked peptides, as shown previously with pBVS (Fig. 2, *B* and *C*) (15). Crosslinkers that cleave under HCD conditions improve the fragmentation of both peptides (23); however, for vinyl-sulfone-based crosslinks, the retro-Michael addition liberates an unmodified peptide, causing the site of crosslinking to be lost (Fig. 2*C*). Other crosslinkers, like DSSO, cleave internally to produce “stubs,” which are remnants of the crosslinker, on each peptide that provide compelling evidence that a crosslinked spectral match is not simply the co-isolation of two gas-phase associated peptides (2, 24). We decided to test whether we could treat the entire vinyl sulfone crosslinker as such a “stub” on both peptides, which cannot occur on gas-phase associated peptides (Fig. 3*A*). We also needed to optimize acquisition conditions that would allow these stubs to be utilized.

We crosslinked HSA with Alkyne-BVSC *in vitro*, performed multiple crosslinking MS data acquisitions using different stepped HCD regimes, and processed our crosslinking MS data using the Xi software suite (Rappsilber lab, see methods) (25). We then tested different post-search filtering approaches to determine the usefulness of these “stubs” for boosting sensitivity of identifying true crosslinks, based on a target-decoy-based false-discovery rate (FDR). We utilized a stepped collision energy HCD, where we fixed two higher energies for typical peptide bond fragmentation, and altered the lowest collision energy from 9 to 21 (NCE, in intervals of 3, Fig. 2*D*). We tested five different post-search filtering approaches before using xiFDR to estimate a 1% FDR at the crosslinked spectrum match (CSM)-level (Fig. 3). The first approach does not filter the CSMs (no filter, or “NF”). The second approach requires matching three crosslinked-fragments (CF), which has been used for MS-cleavable crosslinkers that do not generate stubs on both peptides, like sulfo-SDA (see methods; Fig. 3*B*) (26, 27). The next three use some form of stub-based filtering: stub method A—requires matching both intact peptide masses plus the crosslinker stub (P-S); stub method B—same as method A but three stub-containing fragments can replace the P-S for one peptide; stub method C—same as B but five stubs on both peptides can replace no P-S being matched in the spectrum (see methods; Fig. 3*C*).

We identified crosslinks on cysteine, lysine, and histidine residues, which concurs with previously published data (Supplemental Table S1) (15). We obtained a maximum of 255 CSMs at a low-energy HCD step of 21 (NCE) without filtering, 196 CSMs at an NCE of 18 for the CF filter, 69 CSMs at an NCE of 18 for Stub method A, 166 CSMs at an NCE of 21 for Stub method B, and 238 CSMs at an NCE of 18 for Stub method C (Supplemental Table S2). These filters provided significant changes to the distributions of matched target and decoy CSMs (Supplemental Fig. S5). The HCD steps of 18 or 21 produced similar numbers of CSMs regardless of filtering technique (Supplemental Table S2, Fig. 2*D*). Surprisingly, no filter (NF) actually outperformed all filtering methods for CSMs at 1% FDR (Supplemental Tables S2 and S3), but this may be due to analyzing a single protein that does not need to deal with the n^2^ problem of increasing potential peptide combinations as the number of proteins in the sample increases (28).

To test these filters in a system more representative of typical crosslinking MS applications, we pulled down proteasomes through a biotin-tagged PSMD2 (RPN1) (29), a subunit of the human 19S regulatory particle of the 26S proteasome, derived from an engineered, colon cancer cell line (HCT116). We then crosslinked the proteasomes with Alkyne-BVSC (250 μM and 1 mM, which were combined after treatment) and analyzed the samples using our crosslinking MS workflow. Guided by the HCD-dose experiment, we used a stepped collision HCD regime of 18/26/30 NCE. We tested the five different post-search filter methods, setting the FDR to 5% at the residue-pair level. We identified 126 inter-protein unique residue-pairs (URPs) without filtering, 167 URPs using CF filtering, 101 URPs using stub method A, 113 URPs using stub method B, and 232 URPs using stub method C (Supplemental Table S4). We opted for a 5% residue-pair FDR, as we did not identify enough inter-protein crosslinks to justify the same 1% FDR used for the stub-based filtering approach. Stub method C outperforms not using any post-search filter (NF) by 84% on inter-protein URPs from a total of 648 URPs. We also conducted the same experiment with DSSO (same concentrations), but instead applied a doublet-based filtering approach before FDR. In this DSSO experiment, we identified 648 inter-protein URPs (1870 total), which demonstrates how much more reactive NHS-esters are than vinyl sulfones (Supplemental Table S5).

**Fig. 4:**
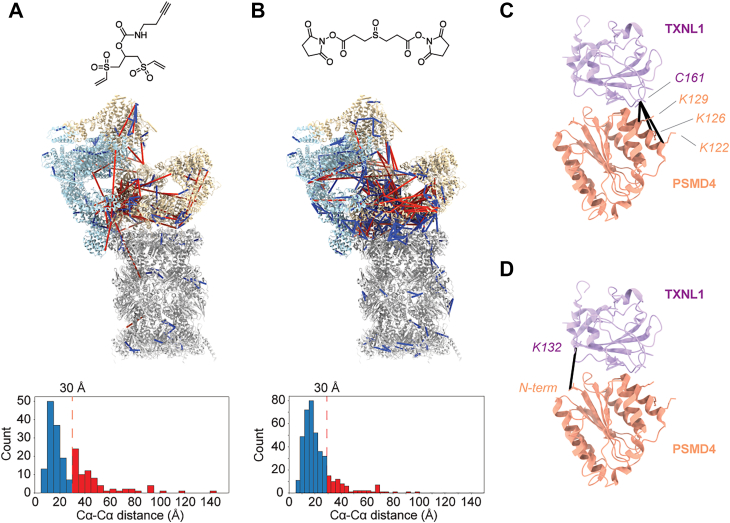
**Vinyl-sulfone-based crosslinkers elucidate residue-pair interactions in a purified proteasome sample.** Crosslinking MS data comparing Alkyne-BVSC (*A*) and DSSO (*B*) for PSMD2 pulldowns. Crosslinks (*blue* lines – within distance restraint of 30 Å, *red* lines – over 30 Å) were mapped onto a 3D structure of the 26S (19S + 20S) proteasome (PDB ID: 6MSE (30)). The 20S core particle is colored in *grey*, while the 19S regulatory particle is split into two colors: light *blue* (lid) and wheat (base). Shown below each structure is a histogram for the number of crosslinks plotted against Cα-Cα distance on 6MSE for Alkyne-BVSC (*left*) and DSSO (*right*) with the same color scheme (*n = 1*). *C*, structure of TXNL1 (*purple*) with PSMD4 (*orange*), with Alkyne-BVSC crosslinks (*black*) mapped as pseudobonds using ChimeraX (PDB ID: 9E8I (33)). Crosslinked residue-pairs are highlighted. *D*, same as (*C*) but for the single DSSO crosslink.

We mapped the crosslinks onto a 3D structure of the 26S (19S regulatory particle plus 20S core particle) proteasome (Fig. 4*A*, PDB ID: 6MSE (30)) to determine how many were overlength (>30 Å Cα-Cα distance). In all, 200 inter-protein URPs from Alkyne-BVSC could be mapped to this structure, 74 of which (37%) were found to be overlength. Comparatively, we found 470 inter-protein URPs from DSSO, 84 of which (18%) were overlength. All of these overlength crosslinks are located in the 19S proteasome, which is known to be very dynamic (29), and homobifunctional crosslinkers, such as NHS-esters, are known to create a large number of overlength links (31, 32). Importantly, the protein coverages provided by Alkyne-BVSC and DSSO were complementary, as Alkyne-BVSC was able to find combinations of lysine, cysteine, and histidine residue pairs, along with the NHS-ester-accessible lysine-to-lysine crosslinks (Supplemental Table S6). Finally, Alkyne-BVSC was able to localize TXNL1 to PSMD4 (RPN10) with 3 URPs between cysteine-161 on TXNL1 and lysines 122, 126, and 129 on PSMD4. The TXNL1-PSMD4 interface was only recently characterized by cryo-EM (PDB ID: 9E8I (33), Fig. 4*C*). A single TXNL1-PSMD4 crosslink was also identified with the DSSO data, but only to the *N*-terminus of PSMD4, demonstrating the complementarity of the added chemical space afforded by vinyl sulfones (Fig. 4*D*).

In summary, we identify the optimal range of low-energy HCD (18–21) to generate stubs inherent to vinyl-sulfone-based crosslinks. These stubs can be used to boost the sensitivity of crosslink identifications. We show that vinyl sulfones can access residue pairs normally inaccessible to NHS-esters to provide orthogonal proteome coverage, expanding the chemical crosslinking space.

### Highly Efficient Affinity Enrichment of Alkyne-BVSC-Crosslinked Peptides

To test the enrichment of our Alkyne-BVSC crosslinks, we crosslinked HSA, appended on desthiobiotin via CuAAC, and enriched the digested, biotinylated peptides via avidin beads. An aliquot of this sample, as an unenriched control, showed a >10-fold reduction in the ratio of intensity of unmodified peptides to monolinks and a ∼22-fold reduction in the overall intensity of unmodified peptides (Supplemental Table S7). In parallel, HSA was crosslinked with the enrichable, NHS-ester-based crosslinker, Alkyne-A-DSBSO (19) (DSBSO), and processed identically for comparison. After crosslinking MS analysis for both datasets, we identified 266 and 80 URPs for Alkyne-BVSC and DSBSO, respectively, at a 1% FDR at the residue-pair level (Supplemental Table S8). As expected, Alkyne-BVSC generated crosslinks primarily between lysine and cysteine, and DSBSO generated crosslinks primarily between lysines, further demonstrating the complementarity afforded by vinyl sulfones when compared to NHS-esters (Supplemental Fig. S6).

To further demonstrate that our enrichment can deplete non-crosslinked peptides, we performed Alkyne-BVSC crosslinking on HSA, clicked on desthiobiotin via CuAAC, and digested the protein with trypsin. We then mixed the crosslinked HSA peptides 1:1 (w/w) with *Escherichia coli* peptides, followed by avidin-bead enrichment. Our stub-based crosslinking analysis on this enriched mixture identified 950 CSMs (357 URPs) at a 1% CSM FDR (Supplemental Table S9). For comparison, we detected 1646 unique, HSA CSMs (429 URPs) at the same FDR for an aliquot of the same crosslinked, enriched HSA sample that had not been mixed with *E. coli* peptides (Supplemental Table S9). An explanation for the difference in these two numbers is that the enrichment does not completely remove all linear peptides, so the mass spectrometer will waste time selecting linear peptides for fragmentation. Linear peptide analysis demonstrated that enrichment starkly reduced linear peptides while boosting monolinks and crosslinks (Supplemental Fig. S7). Without any enrichment (or CuAAC), we detected only 53 and 122 CSMs at the same FDR for HSA peptides mixed or not mixed with *E. coli* peptides, respectively (Supplemental Table S9).

### Vinyl-Sulfone-Based Crosslinkers are Quenched by Intracellular Glutathione

To determine whether our novel crosslinkers could be used to analyze in-cell mammalian and bacterial data for *in situ* applications, we utilized fluorescence microscopy to determine whether Alkyne-BVSC was entering HeLa cells. After *in situ* crosslinking, HeLa cells were permeabilized and rhodamine was clicked onto the alkyne handle present on the crosslinker to visualize the extent of crosslinking within the cells. Alkyne-BVSC was readily able to enter cells and to disperse throughout the cell cytoplasm (Supplemental Fig. S8). Interestingly, when we performed this assay in parallel for DSBSO, we noted large aggregates that formed inside cells (Supplemental Fig. S8). Previous research has demonstrated visible distortion of the actin cytoskeleton in A549 cells after *in situ* DSS crosslinking, although not to the extent that we have shown here (34). Disappointingly, SDS-PAGE of the lysates from the Alkyne-BVSC-crosslinked cells suggested that very little crosslinking was occurring under these conditions (Supplemental Fig. S9).

Regardless, we performed *in situ* crosslinking MS with Alkyne-BVSC in HeLa and *E. coli* cells, as it was possible that the crosslinking was not observable by SDS-PAGE. Unfortunately, we were unable to detect much crosslinking: enrichment of the crosslinked peptides revealed only 224 (HeLa) and 8 (*E. coli*) crosslinks, respectively, at a 5% residue-pair FDR (CF filtering, as stub-based was too computationally intensive, Supplemental Table S10). The reason for this became apparent after performing an Open modification search (21) using FragPipe/MSFragger (22) that found cysteines, histidines, and lysines modified with a mass of 1056, consistent with Alkyne-BVSC reacting with intracellular glutathione (Supplemental Fig. S10, Supplemental Table S11). We know from the SDS-PAGE experiment that Alkyne-BVSC is very efficiently quenched by glutathione *in vitro* (Fig. 1*D*), and considering that the concentration of glutathione in the cells is estimated to be in the millimolar range (35), this is probably a dead-end for in-cell applications of vinyl-sulfone-based crosslinkers.

### A Novel, Vinyl-Sulfone-Diazirine, Heterobifunctional Crosslinker: VSD

**Fig. 5:**
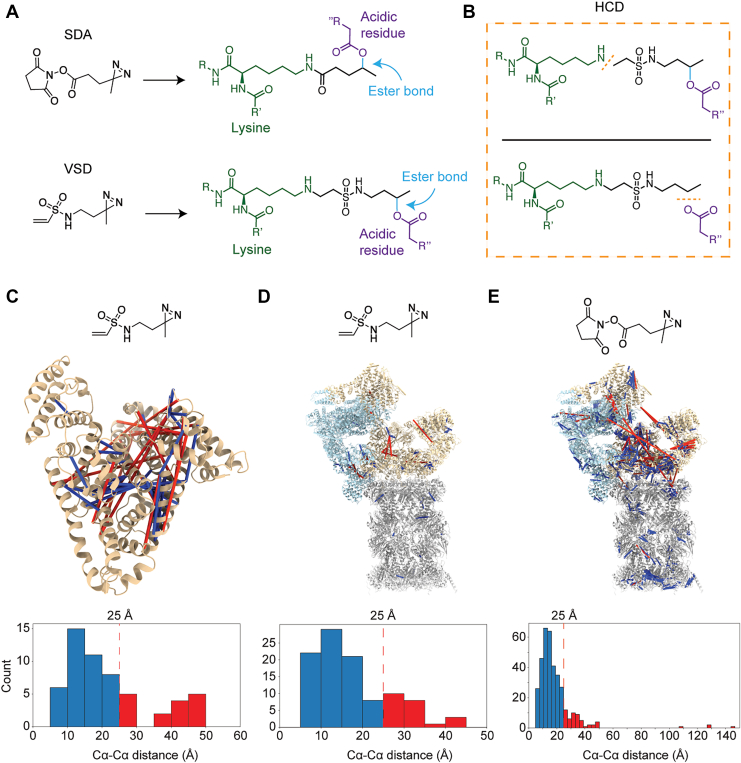
**VSD is a novel chemical photo-crosslinker.***A*, chemical structures of both SDA (*top*) and VSD (*bottom*) and their theoretical reactions with lysine (*green*) and an acidic residue (*purple*). *B*, the resulting ester (*blue*) and the vinyl sulfone bonds can be fragmented by low-energy HCD (NCE ∼20, *orange* dashed line). *C*, chemical structure of VSD (*top*), annotated crosslinks (*blue* lines – within distance restraint of 25 Å, *red* lines – over 25 Å) mapped onto a single-chain of HSA (from 1AO6 (38)), and a histogram of crosslinks binned by Cα-Cα distance (*n = 1*). *D*, Same as *C*, but the structure displayed is a model of the 26S proteasome (6MSE (30), *n = 1*). *E*) Same as D, but for SDA (*n = 1*).

Photo-crosslinkers containing diazirines capture protein interactions with high spatial and temporal precision by generating short-lived reactive intermediates after activation by UV light. This minimizes background from transient or non-specific encounters, making photo-crosslinkers particularly useful for structural studies (31, 32). UV-activated diazirines have broad amino acid reactivity; however, heterobifunctional crosslinkers that combine NHS-ester functionality on one side with diazirine functionality on the other, such as SDA, are limited by the specificity of the NHS-ester (27, 36). Vinyl sulfones can target protein surfaces inaccessible to NHS-esters, so we synthesized a crosslinker that contains both a vinyl sulfone and a diazirine, called VSD (Vinyl Sulfone Diazirine, Fig. 5*A*). Just like Alkyne-BVSC, VSD can fragment via HCD at either side of the crosslinker, but only when the diazirine performs O-H or N-H insertions, as a C-H insertion will form a bond that is not typically fragmentable by the collision energies we utilize, preventing the consistent and predictable formation of ‘stubs’ for diazirine-based crosslinkers (37).

To demonstrate the utility of this crosslinker, we crosslinked HSA with VSD *in vitro* and analyzed the data using our crosslinking MS workflow. We annotated 162 URPs at a 5% residue-pair FDR; cysteine was the most frequently annotated residue (Supplemental Table S12). 123 of the 158 (∼78%) crosslinks found on the monomeric HSA (PDB: 1AO6 (38)) were within a Cα-Cα distance of 25 Å, the estimated distance restraint allowed by VSD (Fig. 5*C*). We opted to use a CF filtering technique to analyze VSD crosslinking MS data, which allowed for a direct SDA comparison, as SDA data has previously been analyzed using this CF filtering technique (26, 27). We crosslinked a PSMD2 pulldown with VSD (8–24 mM) or SDA (250 μM and 1 mM) and performed the same crosslinking MS workflow on each. In summary, we identified 155 and 728 URPs for VSD and SDA, respectively, at a 5% residue-pair FDR, 102 and 360 of which can be mapped onto the 6MSE proteasome structure, respectively (Supplemental Tables S13 and S14). Both VSD and SDA provide dozens of structurally relevant crosslinks, as determined by their estimated distance constraints (78% for VSD and 85% for SDA, Fig. 5, *D* and *E*). VSD does increase the residue-pair coverage that is afforded by SDA, owing to the vinyl sulfone reactive group; however, this data is best regarded as complementary, as NHS-esters are far more reactive than vinyl sulfones. This is evident by the ∼4.5x number of unique residue pairs identified in the SDA dataset compared to the VSD dataset, as well as the lack of inter-protein crosslinks detected by VSD (Supplemental Tables S13 and S14). Interestingly, in a result that highlights how comparatively unreactive vinyl sulfones can be, an Open modification search found nearly 1% of peptide spectral matches (PSMs) in our VSD PSMD2 crosslinking dataset to contain a modification that can be explained by the diazirine reacting with a protein followed by glutathione reacting with the vinyl sulfone during the reaction quench step (Supplemental Fig. S11). Additionally, only about half of a percent of PSMs were found to contain a VSD monolink, where the vinyl sulfone had reacted with cysteine, lysine, or histidine, and the diazirine had been quenched by bulk solvent (Supplemental Table S15). In a stark contrast, in the SDA dataset, we find that nearly 13% of PSMs identified by the Open modification search (21) contain an SDA monolink (Supplemental Table S16).

We synthesize and characterize a novel, vinyl-sulfone-diazirine crosslinker, VSD, and demonstrate its ability to discover structurally relevant crosslinks in HSA and in purified proteasome that complement the chemical crosslinking space accessible to NHS-esters.

## Discussion

In this study, we introduce a new class of vinyl-sulfone-based crosslinkers designed to expand the chemical and experimental landscape of crosslinking mass spectrometry. We developed Alkyne-BVSC and related analogs: homobifunctional vinyl sulfone crosslinkers that combine MS-cleavability, acid-cleavability, and enrichment compatibility. These features address longstanding limitations of chemical crosslinkers for analyzing heterogeneous protein mixtures. The BVSS series of silane-based crosslinkers demonstrate the nuance required to balance solubility and acid-cleavability. We also introduce a heterobifunctional vinyl-sulfone-diazirine crosslinker, VSD, to expand residue pairs that can be captured on protein complexes.

Compared to NHS-ester crosslinkers, vinyl sulfone crosslinkers maintain reactivity in the presence of common biochemical additives and expand residue specificity to include cysteine and histidine. This extension of chemical space can capture additional structural information that is complementary to that already afforded by NHS-ester-based crosslinkers. We demonstrated this with crosslinks from both DSSO and Alkyne-BVSC on an endogenous human proteasome pulldown that together describe the interface between the thioredoxin-like protein, TXNL1, and a subunit of the 19S regulatory particle of the proteasome, PSMD4 (RPN10), an interaction only recently reported (33). Our vinyl-sulfone-diazirine crosslinker, VSD, complements current photo-crosslinkers by targeting regions of proteins that lack accessible lysines, diversifying the reach of photo-crosslinking into previously inaccessible structural contexts. We believe that this work demonstrates the broad potential for further development of heterobifunctional crosslinkers.

We introduce a novel stub-based post-search filtering strategy that leverages the inherent MS-cleavability of vinyl-sulfone-derived linkages. We demonstrate that this stub-based filtering technique boosts the sensitivity of identifications of vinyl sulfone crosslinks from complex mixtures. By treating fragment ions that contain the HCD-fragmented crosslink (i.e., a stub) as evidence of correct peptide identification, we were able to boost the number of inter-protein URPs in the PSMD2 (RPN1) pulldowns by 84% (Supplemental Table S4). It may be possible in the future for crosslinking search software to be able to add weight to these stubs in scoring functions, so that this type of post-search filtering becomes unnecessary. Stub-based and expected-fragment-based post-search filtering strategies may prove to be increasingly important as new, more sensitive mass spectrometers produce much more complicated fragmentation spectra (39).

There is still much development needed in crosslinker chemistry. There is a need for crosslinkers that can penetrate deep into cells to capture structural information on proteins and to characterize their interactions. Due to the complexity of these samples, crosslinkers must include an enrichable element to capture the full dynamic range of the proteome. NHS-ester-based crosslinkers, such as DSBSO, are very reactive and, consequently, can react with proteins toward the cell surface before they penetrate the cellular interior. Effectively, this diminishes the concentration of crosslinker able to assay proteins in the cytoplasm and the nucleus, limiting the number of detectable crosslinks from these regions. Furthermore, cells must be removed from growth media before crosslinking with NHS-esters, which can alter their interactome (34). Vinyl sulfone crosslinkers, with their lower reactivity, were a promising candidate for this, but we demonstrate that they are efficiently quenched by intracellular glutathione. Future work will continue to evaluate reactive groups for crosslinkers that strike a balance between reactivity and viability.

As always, there are limitations with the novel research employed in this work. For crosslinking MS, we utilize high-resolution MS1 and MS2 Orbitrap scanning. We did not benchmark our results for other types of mass spectrometers or for low-resolution data. Potentially, expected-ion filtering, like the stub-based approach, could produce even larger gains for those data types. Additionally, we utilized the Xi crosslinking search software in this paper because it generates an annotation for each individual peak prior to a separate FDR calculation. These key features are critical for our stub-based filtering approach. We did not benchmark against other crosslinking search software, as this comparison was not the goal of this work. Taking these considerations into account, we believe that the work performed herein is both robust and impactful for the field of crosslinking MS.

In conclusion, our results characterize vinyl-sulfone-based crosslinkers and establish them as a valuable addition to the chemical toolbox for crosslinking MS. By enabling reactivity toward previously underutilized residues and tolerating conditions that quench NHS-esters, we demonstrate that vinyl-sulfone-based crosslinkers serve as versatile and complementary tools for structural proteomics.

## Experimental procedures

### Materials

All solvents for mass spectrometry are LC-MS-grade unless otherwise stated: water with 0.1% formic acid (Thermo, cat#85171), acetonitrile with 0.1% formic acid (ProteoChem, cat#LC6312-1L), acetonitrile (“ACN”, Fisher Scientific, cat#AA47138M6), formic acid (Thermo, cat#85178), methanol (PerkinElmer, cat#N9304938). Other solvents are HPLC-grade unless otherwise stated: acetone (Sigma-Aldrich, cat#270725-4L), dimethylsulfoxide (“DMSO”, anhydrous, Sigma-Aldrich, cat#276855-100ML), tert-butyl alcohol (ACS-grade, 99+%, Thermo, cat#AA33278AK). The following reagents were used: desthiobiotin PEG-3-azide (Sigma-Aldrich, cat#902020), tris(2-carboxyethyl)phosphine hydrochloride (“TCEP”, Sigma-Aldrich, cat#C4706), tris[(1-benzyl-1H-1,2,3-triazol-4-yl)methyl]amine (“TBTA”, Sigma-Aldrich, cat#678937), copper(II) sulfate pentahydrate (“CuSO_4_”, Sigma-Aldrich, cat#209198), TAMRA azide (Click Chemistry Tools, cat# AZ109-5), DL-dithiothreitol (“DTT”, Sigma-Aldrich, cat#43815-5G), iodoacetamide (“IAA”, Sigma-Aldrich, cat#I1149-5G), ammonium bicarbonate (Sigma-Aldrich, cat#A6141-1KG), urea (Sigma-Aldrich, cat#51456), phosphate-buffered saline (Research Products International Corp, cat#P32060-10000.0), human serum albumin (Sigma-Aldrich, cat#A9731), alpha-enolase (baker’s yeast, Sigma-Aldrich, cat#E6126.2.5KU), trypsin (MS grade, Thermo, cat#90057), lys-c (MS-grade, Fukifilm Wako Chemicals, cat#125-05061), trifluoroacetic acid (“TFA”, MS-grade, Fisher Scientific, cat#PI85183), SPE C18 disks (Empore, cat#66883-U), 4x Laemmli sample buffer (Bio-Rad, cat#1610747), beta-mercaptoethanol (“BME”, Bio-rad, cat#1610710), 4 to 20% Mini-PROTEAN TGX Precast Protein Gels (Bio-Rad, cat#4561096, 4561093, 4561094), 10x Tris/Glycine/SDS running buffer (Bio-Rad, cat#1610732), glutathione (“GSH”, reduced form, Sigma-Aldrich, cat#G6529-25G), sodium hydroxide (Sigma-Aldrich, cat#S8045-500G), InstantBlue Coomassie Protein Stain (Abcam, cat#ab119211), BCA protein assay kit (Thermo Fisher, cat#23225), cOmplete EDTA-free protease inhibitor cocktail (Sigma-Aldrich, cat# 5056489001), Dulbecco’s Modified Eagle Medium (‘DMEM’, Thermo Fisher, cat#11995065), Fetal Bovine Serum (‘FBS’, Thermo Fisher, cat#26140079), DNase I (GoldBio, cat#D-300-100), lysozyme, egg white (GoldBio, cat#L-040-25), ultra centrifugal filter, 100 kDa MWCO (Sigma-Aldrich, cat#UFC910008).

*E. coli* (*E. coli*) was obtained from the ATCC (cat#700926) and cells were grown in RPI Luria Broth (“LB”, Miller’s LB Broth, Grainger, cat#31FZ62).

### Chemical Synthesis

Please see supporting methods.

### Gel-Based Crosslinking Assay

A 1 mg/ml solution of human serum albumin or alpha-enolase (baker’s yeast) in phosphate-buffered saline (PBS) was incubated with 20x DMSO stocks of crosslinker for 1 h at 37 °C, or for 30 min at RT for DSBSO, with shaking. For every 50uL of crosslinked solution, 6uL of a master click-chemistry mix containing tetramethylrhodamine-azide, tris(2-carboxyethyl)phosphine hydrochloride (TCEP), tris[(1-benzyl-1H-1,2,3-triazol-4-yl)methyl]amine (TBTA), and copper (II) sulfate (CuSO_4_) was added and reactions were incubated at RT for 1 h with shaking as previously described (40). Crosslinking and click reactions were quenched with addition of glutathione (5 mM as a water stock) for 20 min at RT with shaking. A 4x Laemmli sample buffer (with beta-mercaptoethanol) was added to each sample, samples were boiled at 95 °C for 5 min, and samples were then cooled briefly and run on a 4 to 20% polyacrylamide gel at 150V for 40 min. Gels were imaged with a ChemiDoc Touch using the 565 nm channel and subsequently incubated with InstantBlue Coomassie Protein Stain for at least 15 min at RT with rotation before being imaged using the Coomassie channel.

### *In Vitro* Crosslinking Protocol

Protein solutions were prepared in PBS at a concentration of 1 mg/ml. Crosslinker stock solutions were made from dry compound stocks by resuspending in dry DMSO to give 20x working solutions. For the HSA crosslinking experiments, crosslinkers were made as 20 mM DMSO stock solutions to give 1 mM final concentrations. Protein solutions (in Eppendorf tubes) were flicked to mix and incubated at 37 °C for 1 hour, or for 30 min at RT for DSBSO, with shaking. Optionally, a 9x master CuAAC mix was generated using desthiobiotin PEG-3-azide as previously described (40) and this solution was added to the crosslinked protein solutions, solutions were vortexed to mix and then incubated at RT with shaking for 1 h. Reactions were then optionally quenched with addition of glutathione (5 mM final as a water stock), tubes were flicked to mix and incubated at RT for 20 min with shaking.

### TFA Cleavage Assay

The gel-based crosslinking assay protocol was followed, except CuAAC was stopped by acetone protein precipitation at −20 °C for at least 1 hour. Samples were then spun at 15,000 x G at 4 °C for 10 min and acetone was removed via pipette; samples were allowed to air-dry. Protein pellets were then resuspended in 50 uL of listed concentrations of TFA at given temperatures (RT or 37 °C) and timepoints (1, 2, or 24 h). Acid-cleavage was quenched by addition of Laemmli sample buffer, sodium hydroxide was added to obtain a sample pH of ∼7, and gels were loaded, run, and imaged as described in the gel-based crosslinking assay protocol.

### *In vitro* PSMD2 Pulldown Crosslinking

Purified proteasome was obtained via PSMD2 (RPN1) pulldown as previously described (29). The same *in vitro* crosslinking protocol was followed as above, except crosslinkers were treated at both 1 mM and 250 μM final concentrations, photoactivation of the relevant diazirine crosslinkers was accomplished as previously described (26), and no CuAAC was performed. For VSD, compound was incubated with eluates at 8, 16, and 24 mM before UV-activating, quenching, and combining samples.

### In-Cell *E. coli* crosslinking

*E. coli* (*E. coli*) were grown overnight in 5 ml of LB broth in 10 ml falcon tubes at 37 °C with shaking. 15 h later, 10 ml of cell growth was added to 1L of fresh LB in a 2L flask with an aerated top until cells reached an OD_600_ of 0.8. Cells were then spun at 4,000 x G at 4 °C for 15 min and supernatant was removed. The resulting cell pellet was weighed and resuspended in LB to achieve a concentration of 50 mg/ml of cells in a 50 ml conical. A 20x DMSO stock solution of Alkyne-BVSC (4 mM final) was added and cells were rotated at 37 °C for 1 hour. Crosslinking was quenched with addition of glutathione (50 mM final as a water stock) with rotation at RT for 20 min. Sample was then spun at 4,000 x G at 4 °C for 10 min and the supernatant was poured off before pellets were snap-frozen in liquid nitrogen to store away at −80 °C. The pellet was thawed on ice before resuspension in 50 ml of cold PBS with protease inhibitor, DNase, and lysozyme. The cells were rotated at 4 °C for 20 min before being Dounce homogenized (5 passes of both course and fine). Sample was diluted with 50 ml of cold PBS and subsequent lysis using an EmulsiFlex-C3 (Avestin, five passes at 15,000–20,000 psi). The resulting lysate was spun at 24,000 x G at 4 °C for 30 min to yield the supernatant, which was spun at 100,000 x G at 4 °C for 1 h. Samples were concentrated using a 100,000 Da molecular weight cutoff spin column before incubation with a master CuAAC mix of desthiobiotin PEG-3-azide, TCEP, TBTA, and CuSO_4_ for 1 h at RT with shaking. Reaction was stopped and proteins were precipitated via addition of 4x the volume of ice-cold acetone.

### In-Cell HeLa Crosslinking

HeLa cells were grown in T-175 flasks with 50 ml of DMEM supplemented with 10% FBS to roughly 90% confluency. Media was aspirated and DMEM (20 ml) that contained Alkyne-BVSC (8 mM final as a 20x DMSO stock) was added to cells and flasks were gently swirled before incubation at 37 °C and 5% CO_2_ for 1 h. Media was then aspirated, cells were gently washed with cold PBS (2 × 10 ml), scraped into conicals, and spun at 1,400 x G for 3 min at 4 °C before the supernatant was aspirated. Cell pellets were snap-frozen using liquid nitrogen to store at −80 °C. Pellets were subsequently thawed on ice, resuspended in cold PBS with protease inhibitor, lysed via probe-tip sonication (3 × 1 s pulses at 30% amplitude), and spun at 21,300 x G for 30 min at 4 °C to clarify the supernatant, which was clicked onto desthiobiotin and acetone precipitated to yield protein as described in the previous section.

### Preparation of Crosslinked Peptides for LC-MS or Size-Exclusion Chromatography

Proteins were crosslinked as described above, but instead of addition of sample buffer, proteins were precipitated in acetone at −20 °C overnight. Pellets were then spun at 15,000 x G at 4 °C for 10 min and acetone was removed via pipette; samples were allowed to air-dry. For samples with large amounts of liquid, samples were spun at 4,000 x G. Proteins were resuspended in an 8 M/100 mM urea/ammonium bicarbonate solution, and a BCA assay was used to determine protein concentration; samples were subsequently diluted to 1 to 3 mg/ml. Proteins were then reduced with dithiothreitol (5 mM) for 30 min at RT with shaking. Solutions were incubated with iodoacetamide (15 mM) for 20 min at RT in the dark. Reactions were quenched with re-addition of DTT (5 mM) and urea concentration was diluted to 1.6 M using 100 mM ammonium bicarbonate. Samples were incubated with trypsin (1:50 protease:protein) overnight at RT with shaking. For the HSA crosslinked samples, the Lys-C digest was skipped.

The following day, samples were either: (A) acidified with TFA or formic acid (depending on the experiment) to pH three and a C18 stage-tip clean-up was performed or (B) diluted with PBS and incubated with streptavidin beads for 1 h at RT with rotation. For the latter, beads were pre-washed with PBS (3 × 10 ml, 1,400 x G spin for 1 min at 4 °C) and aliquoted as per the manufacturer's recommendation based on the amount of protein in the sample. Beads were washed with ammonium bicarbonate (25 mM, 3 × 10 ml, 1,400 x G spin for 3 min) followed by sterile-filtered water (3 × 10 ml, 1,400 x G spin for 3 min). Beads were transferred to protein lo-bind tubes and liquid was removed after another spin. Beads were then incubated with 50% acetonitrile with 0.1% formic acid for 3 min at RT, followed by spinning at 1,400 x G spin for 3 min. Supernatant was pipetted into a protein lo-bind tube; this process was repeated twice. Samples were dried *in vacuo* before desalting with C18 stage tips to elution and then drying again *in vacuo*.

Stage tips (41) were made in-house using Empore SPE Disks by packing three C18 pieces on top of one another in a 200 μL pipette tip. Stage tips were activated using 20 uL of methanol, and the methanol was washed out twice using 20 μL of 80% acetonitrile, followed by two more washes of 0.1% TFA. All samples were loaded onto the stage tips and washed twice with 20 μL of 0.1% TFA. To elute samples from still-wet stage-tips, two elutions of 10 μL of 80% acetonitrile were performed. To elute samples from the dry stage tips, 10 μL of methanol was pushed almost all the way through the stage tip, followed by 2 × 10 uL of 80% acetonitrile, with the first elution pushed almost all the way through and the second all the way to complete dryness. Eluted peptides were collected in protein lo-bind tubes and dried *in vacuo*. Stage tips were never allowed to go to full dryness unless storing washed, loaded samples at −20 °C.

### LC-MS Data Collection

Dried peptides were resuspended in 10 uL of 1.6% acetonitrile with 0.1% formic acid, vortexed, and sonicated for 1 min before injecting 1 ug of estimated peptide sample onto the Thermo Eclipse Orbitrap coupled to a Vanquish Neo HPLC system. Peptides were ionized using an EASY-Spray source and eluted over an EASY-Spray PepMap Neo 75 um × 500 mm C18 column (heated to 40 °C) with LC-MS quality water or acetonitrile containing 0.1% formic acid (mobile A and B, respectively) with the following gradients (%B): early peptide size-exclusion samples: 300 nl/min flow rate, 0 to 1 min (1.6%), 1 to 10 min (1.6–17.6%), 10 to 87 min (17.6–32.0%), 87 to 92.5 min (32.0–44.0%), 92.5 to 95 min (44.0–76.0%), 400 nl/min flow rate, 95 to 100 min (76.0%); middle peptide size-exclusion samples: 300 nl/min flow rate, 0 to 1 min (1.6%), 1 to 10 min (1.6–10.4%), 10 to 87 min (10.4–32.8%), 87 to 92.5 min (32.8–44.0%), 92.5 to 95 min (44.0–76.0%), 400 nl/min flow rate, 95 to 100 min (76.0%); late peptide size-exclusion samples: 300 nl/min flow rate, 0 to 1 min (1.6%), 1 to 10 min (1.6–7.2%), 10 to 87 min (7.2–25.6%), 87 to 92.5 min (25.6–44.0%), 92.5 to 95 min (44.0–76.0%), 400 nl/min flow rate, 95 to 100 min (76.0%). Note that the first biological replicate for the HSA HCD dose experiment was performed with the above gradient but using an UltiMate 3000 RSLC nano LC system with an Acclaim PepMap 100 C18 3 μm, 75 μm × 2 cm trap column and with the analytical column heated to 50 °C.

Peptides were analyzed using the following MS global parameters: method duration of 85 min, infusion mode – liquid chromatography, expected LC peak widths – 30 s, advanced peak determination checked, default charge state of 2, EASY-IC internal mass calibration, NSI ion source, static spray voltage at 2000V in positive mode, static gas mode with a sweep gas setting of two and ITT temperature of 280 °C. Samples were collected using the following shared scan parameters: Duty-cycle of 3 s, MS-OT at 240k resolution, normal mass range, quadrupole isolation checked, scan range of 380 to 2000, RF lens of 35%, a custom AGC target of 150% with a max injection time of 100 ms, 1 microscan in profile mode at positive polarity with a source fragmentation of 10V, EASY-IC checked; subbranch MIPS – peptide; subbranch intensity – 2.5e4, subbranch charge state 3 to 7, subbranch dynamic exclusion of one time after 30 s with a mass tolerance of 10 ppm, exclude isotopes and dependent scan on single charge state checked; subbranch priority 1: subbranch A: charge state – 4, precursor selection range – 380 to 1800, subbranch B: charge state – 5, precursor selection range – 380 to 1350, subbranch C: charge state – 6 to 7, precursor selection range – 380 to 1000; rejoined subbranch: sort by intensity – most intense, subbranch ddMS2 OT HCD: isolation mode – quadrupole with a window of 1.4 m/z, stepped HCD (normalized) of 18, 26, and 30, detector type – orbitrap at 60k resolution from 150 to 2000 m/z, AGC target of 750% with a max injection time of 150 ms, 1 microscan, centroid data. Subbranch priority 2: subbranch: charge state – 3, precursor selection range – 380 to 2000, subbranch: sort by intensity – most intense, subbranch ddMS2 OT HCD: isolation mode – quadrupole with a window of 1.4 m/z, stepped HCD (normalized) of 18, 26, and 30, detector type – orbitrap at 60k resolution from 150 to 2000 m/z, AGC target of 750% with a max injection time of 150 ms, 1 microscan, centroid data. For the HCD energy dose-response HSA experiment, the lowest of the three HCD energies was changed to 9, 12, 15, 18, or 21, while the higher two energies remained the same and a 95-min method duration was used. For DSBSO crosslinked samples, we used a stepped HCD of 20, 26, and 30 with the rest of the method being the same; these energies closely mirror what has been used previously (42). For the VSD and SDA experiments, the above standard method was used, but with a stepped HCD with NCEs of 20, 26, and 30, an MS2 max injection time of 250 ms, and RunStart Easy-IC internal mass calibration. For the RPN1 experiments, a second injection of each fraction was performed using the relevant base method, except that the scan and precursor selection ranges were narrowed to 650 to 905 m/z.

The HSA HCD dose-response crosslinking datasets were acquired with the above methods, adjusted to accommodate an UltiMate 3000 RSLC nano LC system with an Acclaim PepMap 100 C18 3 μm, 75 μm × 2 cm trap column.

### Peptide Size-Exclusion Chromatography

Dried peptides were resuspended in 25uL of 30% acetonitrile with 0.1% TFA, vortexed, and sonicated for 1 min before injecting onto a Superdex 30 Increase 3.2/300 column (Cytiva, cat#29219758) with a 20uL injection loop using an Äkta pure micro system and eluting peptides using the following gradient: 0 to 0.03 ml (0.010 μl/min), 0.03 to 0.50 ml (0.010 μl/min), 0.50 to 0.95 ml (0.015 μl/min), 0.95 to 1.45 ml (0.030 μl/min), 1.45 to 1.85 ml (0.040 μl/min), 1.85 to 3 ml (0.040 μl/min). Crosslinked peptides eluted over the range 1.00 to 1.45 ml as 50 μL fractions pipetted into protein lo-bind tubes.

### Crosslinked Peptide Identification Workflow

Raw files were processed using the preprocessing workflow described by the Rappsilber lab (https://github.com/Rappsilber-Laboratory/preprocessing). xiSEARCH (https://github.com/Rappsilber-Laboratory/XiSearch, v. 1.8) (25) was used to perform the crosslinked peptide search. Recalibrated mgfs were searched against a fasta file of the select protein using the following global parameters: methionine oxidation (variable, 147.035395), cysteine carbamidomethylation (fixed, 160.03065, “Ccm”), b and y ions, water loss (S/T/D/E/c-term, 18.01056027), ammonia loss (R/K/N/Q/*N*-terminus, 17.02654493), methionine hydroxylation loss (63/99828547), digestion after K or R except when followed by P (Trypsin), three missed cleavages allowed, non-covalent search, MS1 precursor tolerance of 2.0 ppm, MS2 fragment tolerance of 5.0 ppm, linears evaluated, PeptideIon enabled, three conservative losses, missing monoisotopic automated match, 10 peaks to consider for alpha peptide, three peptide modifications max, 20 modified peptides per peptide match, fragment tree FU, two missing isotope peaks, and minimum charge of 3. The ‘write peak annotations’ box was selected. For Alkyne-BVSC, the following search parameters were added: monolink (variable, K/H/*N*-terminus, 222.0016), cleavable crosslinker mass (0, name: 0; 222.0016, name: S), crosslinker mass (222.0016, K/H/*N*-terminus to K/H/*N*-terminus), crosslinker mass (164.980136, Ccm to K/H/*N*-terminus), crosslinker mass (107.958672, Ccm to Ccm). For BVSS-Me/BVSS-Et/BVSS-Ph, the following search parameters were added: monolink (variable, K/H/*N*-terminus, 240.0166), cleavable crosslinker mass (0, name: 0; 240.0166, name: S), crosslinker mass (240.0166, K/H/*N*-terminus to K/H/N-terminus), crosslinker mass (182.995136, Ccm to K/H/*N*-terminus). For *in situ* experiments, the monolink variable modification was removed. For Alkyne-A-DSBSO, the following search parameters were added: cleavable crosslinker mass (0, name: 0; 54.0106, name: A; 85.9824, name: B; 158.0038, name: S), crosslinker mass (158.0038, K/*N*-terminus to K/*N*-terminus) as previously described (42). For VSD, the following search parameters were added: glutathione quench (variable, D/E, 468.1352), cleavable crosslinker mass (0, name: 0; 161.050964, name: S), crosslinker mass (161.050964, K/H/*N*-terminus to any), crosslinker mass (104.0295, Ccm to any), crosslinker mass (47.008036, Ccm to Ccm).

For Alkyne-BVSC with CuAAC but without TFA cleavage, the following search parameters were added to the base Alkyne-BVSC config: deamidation (variable, N/Q, 0.984016), pyroglutamate (variable, Q (peptide *N*-terminus only), glutathione-quench (variable, K/H/*N*-terminus, 529.08586), uncleaved glutathione-quench (variable, K/H/*N*-terminus, 1056.392), uncleaved glutathione-quench (variable, C, 999.3724), cleavable crosslinker mass (749.30880, name: U), crosslinker mass (749.30880, K/H/*N*-terminus to K/H/N-terminus), crosslinker mass (692.287336, Ccm to K/H/*N*-terminus), crosslinker mass (635.265872, Ccm to Ccm).

For post-search filtering: for CF filtering, the same filters were applied post-search as previously described (26). For stub-based filtering, we used three different methods: method A—require a P-S on both peptides, method B—method A in addition to allowing for one P-S on one peptide and 3 S stubs on the other peptide, and method C—method B in addition to allowing for 5 S stubs on both peptides. See Figure 3 for an illustration of these filters. Custom Python scripts were used to achieve these filters and are available upon request. The non-acid-cleaved crosslinker on the full peptide (P-U) or fragmented peptides (U) was also allowed for these filters. The resulting file that contained a list of these matches was then used to filter the original xiSEARCH CSV file output.

The Python scripts used to achieve this filtering work as follows: the first script filters the unzipped peak tsv annotations file from xiSEARCH for instances of column 13 (‘FragmentName’) that contain the text “_S” and instances of column 21 (‘Description’) that contain the text “crosslinked”. The output of the first script is then filtered by a subsequent script for whether the combination of scan (column 2, ‘ScanNumber’) and peptide type (“alpha” or “beta” denoting the alpha or beta peptide match, column 9, ‘MatchedPeptide’) contains “P_S” or “_S” as text in column 13. If both the alpha and beta peptide for that scan contain “P_S” in any of the rows for column 13, or if one of the alpha or beta peptides contains “P_S” and the other contains three text instances of “_S”, or if both the alpha and beta peptides for that scan contain at least five instances of “_S”, the entire row is written into a new tsv file. Finally, this resulting tsv file is used to filter for scans from the csv xiSEARCH output file. The “S” in “P_S” and “_S” is set in the xiSEARCH configuration as a cleavable crosslinker loss (“loss:CleavableCrossLinkerPeptide:MASS:222.0016;NAME:S”), so this letter is customizable, and more letters can be added for filtering for additional cleavable crosslinker losses. For example, we also searched for “U” the exact same way as “S” for experiments where we appended on desthiobiotin in the above scripts (“loss:CleavableCrossLinkerPeptide:MASS:749.30880;NAME:U”).

The filtered csv was imported along with the xiSEARCH config file and the fasta into XiFDR (https://github.com/Rappsilber-Laboratory/xiFDR, v. 2.2.1) and run with the following parameters for setting a 1% residue-pair FDR: complete FDR selected, PSM: 100, Peptide Pair: 100, Protein Group: 100, Residue Pairs: 1, Protein Pairs: 100, min. pep. length of 5, no consecutive selected, boost separately selected, boost selected: residue pairs with four steps. For RPN1 experiments, the above parameters were changed: Residue Pairs: 5, min. pep. Length of 6, no consecutive not selected, ‘betweens’ selected. For setting a 1% PSM FDR, the following parameters were set: PSM: 1, Peptide Pair: 100, Protein Group: 100, Residue Pairs: 100, Protein Pairs: 100. Boosting on ‘betweens’ was performed optionally, where noted. The resulting mzid and csv files were exported and the CSM, mgfs, and fasta files were uploaded to XiView (43) (https://github.com/Rappsilber-Laboratory/xiview) to visualize crosslinking data.

### Open Modification Search

FragPipe (https://github.com/Nesvilab/FragPipe, v. 22) was used in conjunction with MSFragger (v. 4.1), IonQuant (v. 1.10.27), diaTracer (v. 1.1.5), DIA-NN (v. 1.8.2_beta_8), and Python (v. 3.9.13) (22, 44, 45, 46). ‘Open’ workflow (21) was loaded and run against selected raw files using the selected fasta with reverse sequence(s) incorporated by FragPipe. Two missed cleavages were allowed (‘stricttrypsin’, KR) with a precursor mass tolerance of -150 to 500 Da and a fragment mass tolerance of 20 ppm with a 1% FDR at both the peptide and protein level. For the Alkyne-BVSC *in situ* Open modification search, a custom fasta was generated based on the top 300 HeLa proteins by intensity rank as described in ‘FASTA file generation’ with decoys added using FragPipe’s built-in feature. Additionally, a precursor mass tolerance of -150-1000 Da was utilized.

### FASTA File Generation

The FASTA file used for human serum albumin (P02768, download date: August 2, 2023) was downloaded from UniProt. For experimental samples, protein identifications were generated using the FragPipe ‘HeLa’ workflow with MS1 Quant enabled (Top 3 N) to search against raw files from the unenriched protein sample using the human (taxonomy ID: 9606, Reviewed entries only, download date: March 3, 2025, size: 20,430) or *E. coli* proteome (taxonomy ID: 83333, Reviewed entries only, download date: April 15, 2024, size: 6066), both downloaded from UniProt. Proteins were then filtered for at least 1 × 10^8^ (HeLa, 1998 proteins or *E. coli*, 1919 proteins) or 5 × 10^8^ intensity (proteasome, human, 202 proteins).

### Confocal Microscopy

HeLa cells were seeded at 10,000 cells per well and grown on 8-well dishes (Ibidi, cat#80826) in 300 μL of DMEM (Life Technologies, cat#11995065) supplemented with 10% FBS (Thermo, cat#A3382001) and L-glutamine (ATCC, cat#30-2214) for 48 h at 37 °C, 5% CO2. For cell treatments, media was aspirated and replaced with 300 μL of fresh DMEM containing the chemical crosslinker at noted concentrations. Dishes were gently mixed and incubated for 1 h at 37 °C, 5% CO2. The media was aspirated and cells were washed with 300 uL of PBS once before being incubated with formaldehyde (4% wt/v in PBS made from commercial stock, Sigma-Aldrich, cat#252549) for 20 min under a chemical hood. Cells were then washed with PBS with gentle shaking for 3 min twice before being incubated with 0.2% Triton X-100 in PBS for 5 min with shaking at RT. The PBS washes were repeated, and rhodamine was appended onto crosslinked protein by incubating the CuAAC mix with cells for 1 h at RT with gentle shaking. The PBS washes were repeated, and cells were incubated with a 1:1000 DAPI solution (Thermo, cat#62248) in PBS for 1 h at 37 °C with gentle shaking. PBS washes were repeated and cells were stored in PBS at 4 °C in the dark until ready to image.

Confocal microscopy was performed using an Andor spinning disk confocal on Leica DMi8 base with a 63x water objective lens. Images were captured via a Z-stack of 22 μm with an automatic number of steps for each of the following excitation wavelengths (in order): 561 nm, 488 nm, and 405 nm. Exposure times and laser powers were adjusted based on the sample set.

Custom ImageJ (Fiji) macros were used to process the images to quantify rhodamine intensity normalized to the number of cells in the given image. Custom Python scripts were written to combine output csv files and to generate plot summaries of the corresponding data. Imaris Viewer was used to generate the 3D images.

### Experimental Design and Statistical Rationale

We have performed two independent biological replicates for all experiments, unless otherwise stated. Crosslinking MS experiments can vary greatly, even within samples, as identification of ions to undergo MS2 is highly stochastic, especially considering the stoichiometries of crosslinked peptides within typical proteomics samples. For many of these experiments, only one biological replicate was performed; however, as we do not make any claims about new biology, we emphasize that our results are appropriate for the constraints of the experiments.

## Data Availability

The HSA HCD dose-response crosslinking data can be accessed via PRIDE (PXD065869). The Alkyne-BVSC enriched HSA crosslinking data can be accessed via PRIDE (PXD065859). The Alkyne-BVSC proteasome crosslinking data can be accessed via PRIDE (PXD065858). The DSSO proteasome crosslinking data can be accessed via PRIDE (PXD065912). The VSD HSA crosslinking data can be accessed via PRIDE (PXD065870). The VSD proteasome crosslinking data can be accessed via PRIDE (PXD065871). The SDA proteasome crosslinking data can be accessed via PRIDE (PXD065946). The DSBSO enriched HSA proteomics data can be accessed via PRIDE (PXD065949). The Alkyne-BVSC HSA/*E. coli* enrichment proteomics data can be accessed via PRIDE (PXD065961). The Alkyne-BVSC *E. coli* enriched crosslinking data can be accessed via PRIDE (PXD065958). The Alkyne-BVSC HeLa *in situ* crosslinking data can be accessed via PRIDE (PXD065956).

## Supplemental Data

This article contains supplemental data.

## Conflict of Interest

The authors declare that they do not have any conflicts of interest with the content of this article.
