# Supplementary material for: Expanding the Crosslinking Mass Spectrometry Toolbox With Vinyl Sulfone Crosslinkers
Source: Mol Cell Proteomics. 2025 Oct 21;24(12):101315. doi: 10.1016/j.mcpro.2025.101315 (PMC12682122; doi:10.1016/j.mcpro.2025.101315)
Supplement: Supporting Information [file mmc1.pdf]

Supporting Information *for*

Expanding the crosslinking mass spectrometry toolbox with vinyl sulfone crosslinkers

Anthony Ciancone[1], Haitao Wu[2], Katerina Atallah-Yunes[1], Kitaik Lee[1], Chris Sibley[3], Jesse Spivey[3], Rolf E. Swenson[2], John S. Schneekloth Jr.[3], Francis J. O'Reilly[1,*]

[1]Center for Structural Biology, Center for Cancer Research, National Cancer Institute (NCI), Frederick, MD 21702-1201, U.S.A.

[2]Chemistry and Synthesis Center, National Heart, Lung, and Blood Institute, National Institutes of Health, Bethesda, MD, U.S.A.

[3]Chemical Biology Laboratory, National Cancer Institute, Frederick, MD 21702, U.S.A.

*Corresponding author: Francis J. O'Reilly - [1]Center for Structural Biology, Center for Cancer Research, National Cancer Institute (NCI), Frederick, MD 21702-1201, U.S.A. Orcid ID: https://orcid.org/0000-0001-9258-0150 email: oreillyfj@nih.gov

Table of Contents

A. Supporting Figures
- **I. Selected Annotated Spectra**
- **II. Full-length Gels**

B. Biochemical Methods

C. Chemical Methods

D. Compound Characterization Data

A. Supporting Figures

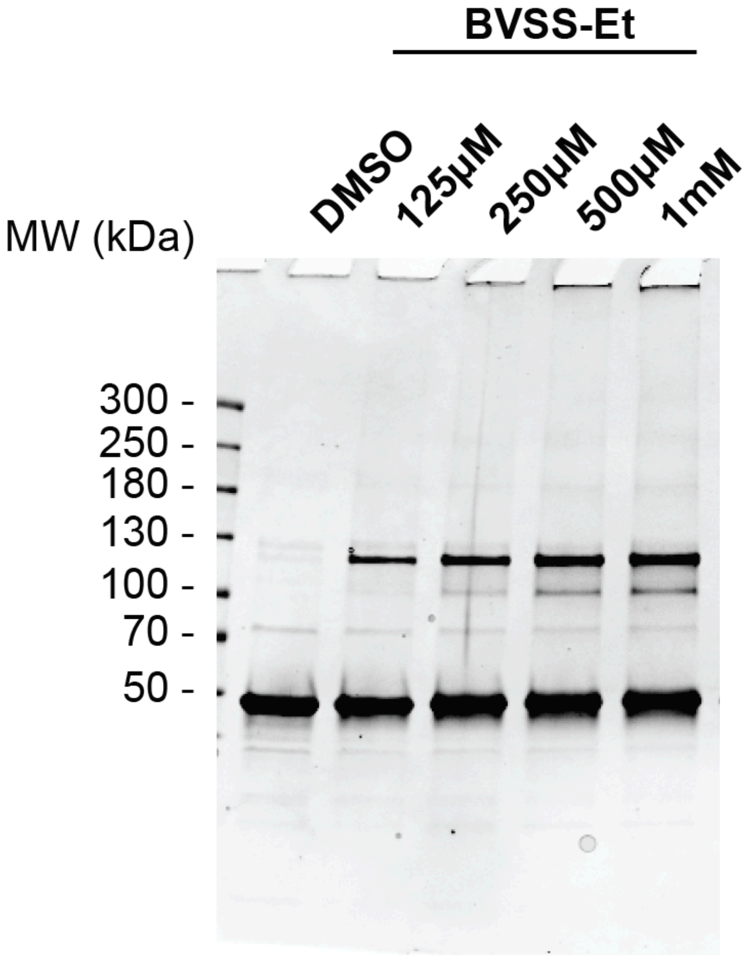

Supporting Figure 1. BVSS-Et crosslinks enolase to stabilize the dimer in an SDS-PAGE experiment. Alpha-enolase was untreated (first lane) or crosslinked with BVSS-Et at increasing concentrations (subsequent lanes, increasing from left to right, *n=2*).

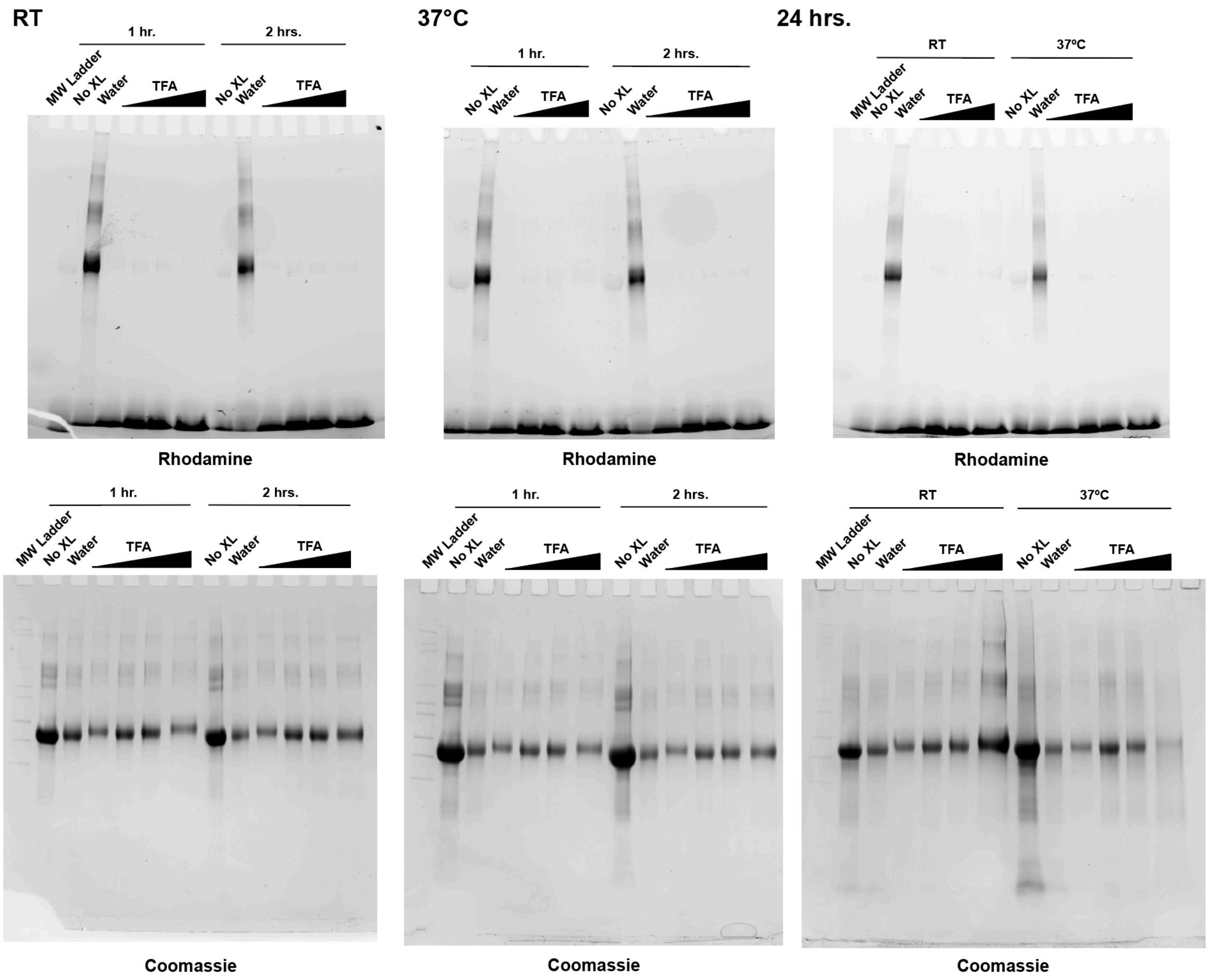

Supporting Figure 2. HSA crosslinked with BVSS-Et *in vitro* then clicked on to rhodamine and subsequently treated with TFA at differing concentrations (0.1%, 0.5%, 1%, and 5%), timepoints (1, 2, and 24 hours), and temperatures (RT and 37ºC, *n=2*).

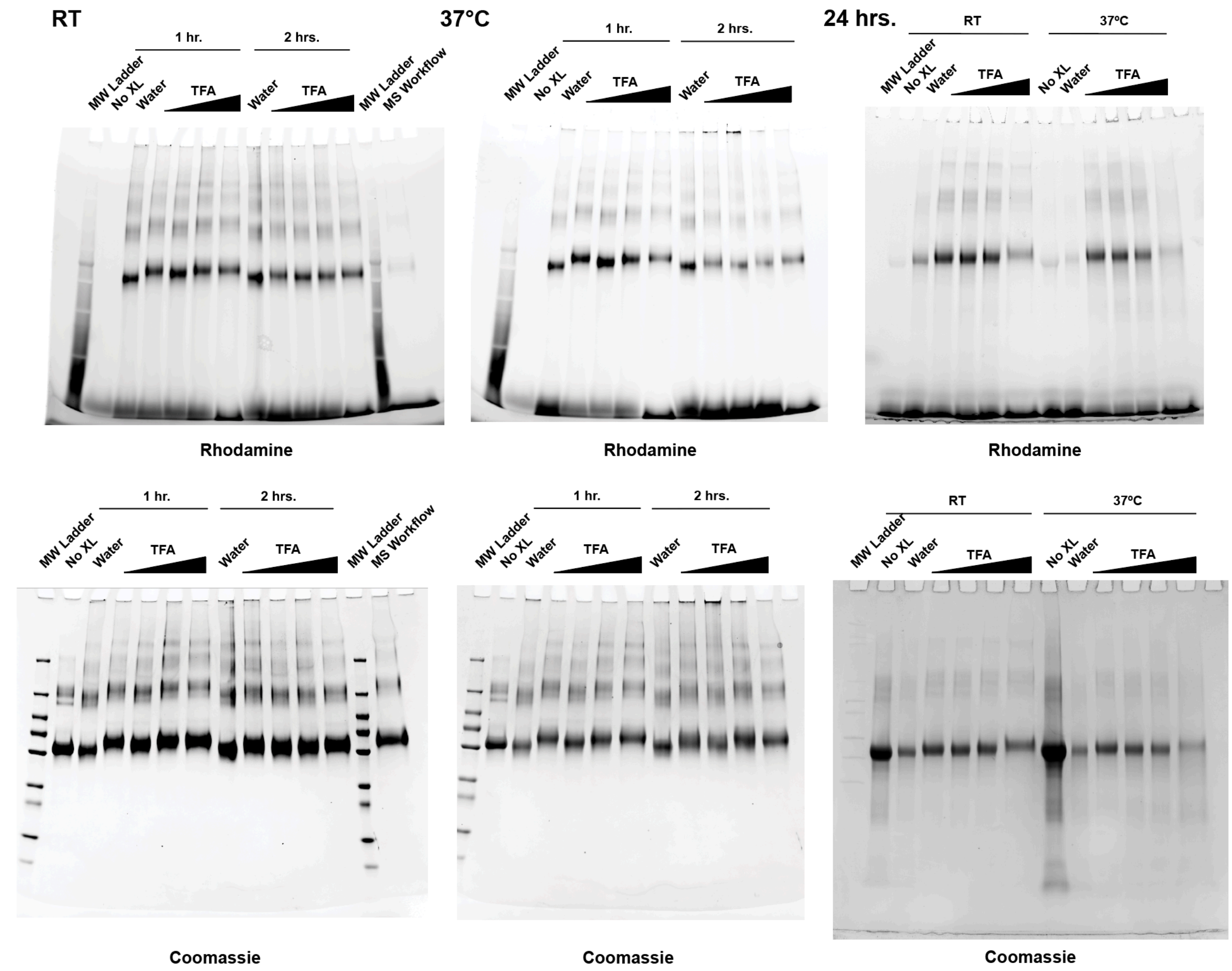

Supporting Figure 3. HSA crosslinked with Alkyne-BVSC *in vitro* then clicked on to rhodamine and subsequently treated with TFA at differing concentrations (0.1%, 0.5%, 1%, and 5%), timepoints (1, 2, and 24 hours), and temperatures (RT and 37ºC, *n=2*).

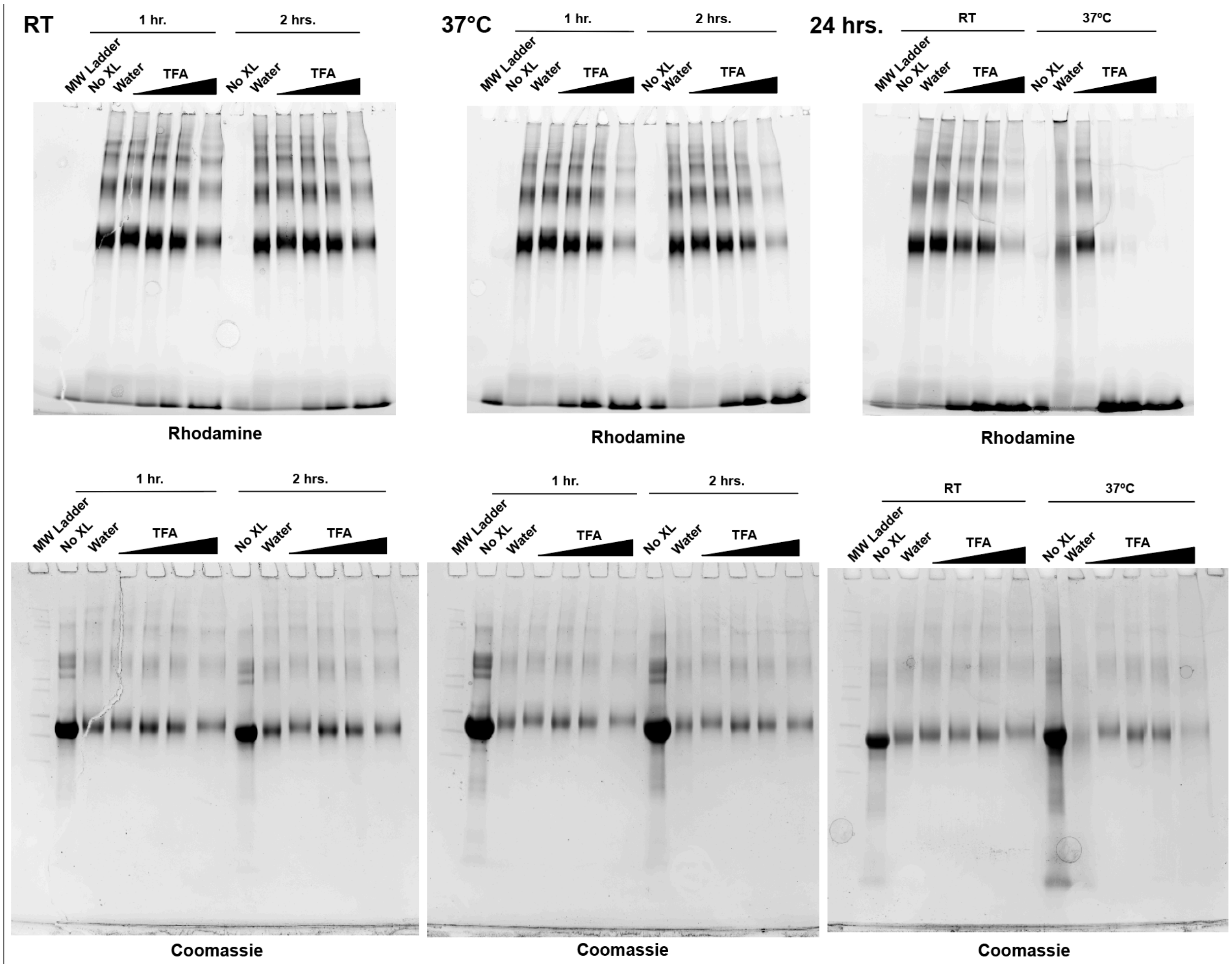

Supporting Figure 4. HSA crosslinked with Alkyne-A-DSBSO *in vitro* then clicked on to rhodamine and subsequently treated with TFA at differing concentrations (0.1%, 0.5%, 1%, and 5%), timepoints (1, 2, and 24 hours), and temperatures (RT and 37ºC, *n=2*).

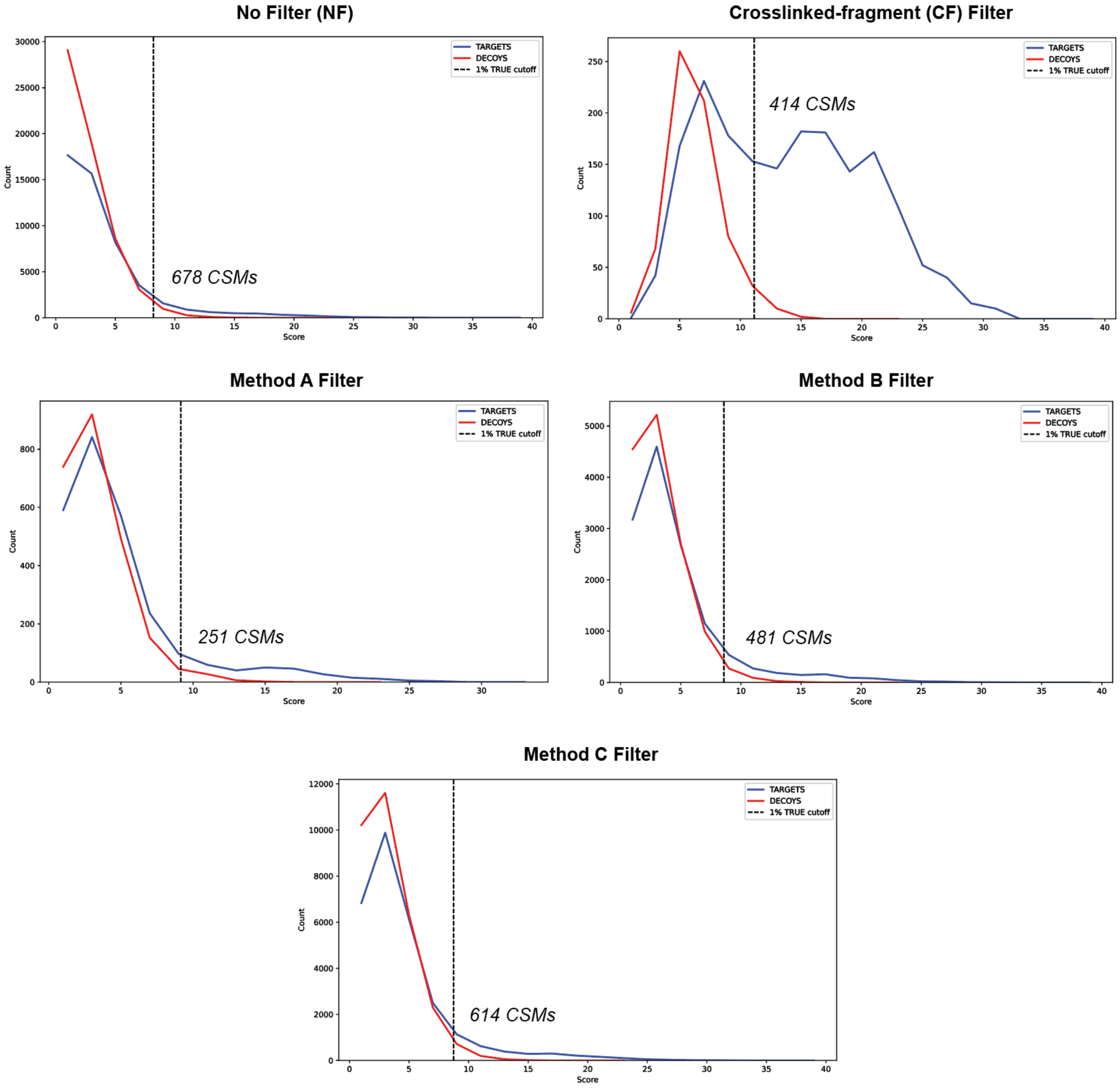

Supporting Figure 5. Targets and decoys plotted by score for each filtering method. Alkyne-BVSC HSA crosslinking data was generated from XiSearch and then post-search filtered using one of: NF, CF, method A, method B, or method C (see methods, **Figure 3**). Python scripts were used to generate histograms that plotted histograms for both the targets (blue lines) and decoys (red lines) at a 1% "real" FDR (black dashed line). No filter (NF) generates the most target CSMs using this type of analysis, but we argue that this is due to low sample complexity (*n=1*).

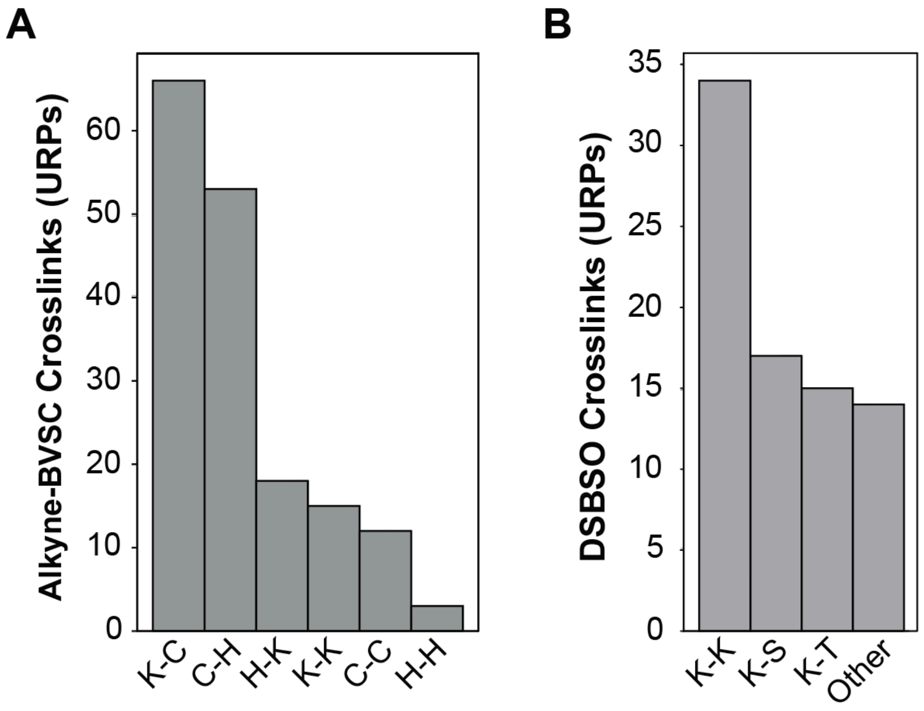

Supporting Figure 6. Residue-pairs annotated from crosslinking data. HSA was crosslinked *in vitro* with Alkyne-BVSC or DSBSO and labeled peptides were appended onto desthiobiotin and enriched via avidin beads. Offline fractionation using size-exclusion chromatography was then used to remove resulting bead contaminants as well as to further enrich for larger peptides (and thus crosslinks). We performed a crosslinking search on both samples using XiSearch and respective configs, before performing stub-based (method C, Alkyne-BVSC) or doublet-based (DSBSO) post-search filtering. We then used XiFDR to threshold our data to a 1% FDR at the residue-pair level and utilized XiView to count the distribution of listed unique residue-pairs (URPs) for both Alkyne-BVSC (**A**) and for DSBSO (**B**). 'Other' indicates a combination of lysine, serine, threonine, and tyrosine residue-pairs not already listed in the plot (*n=1*).

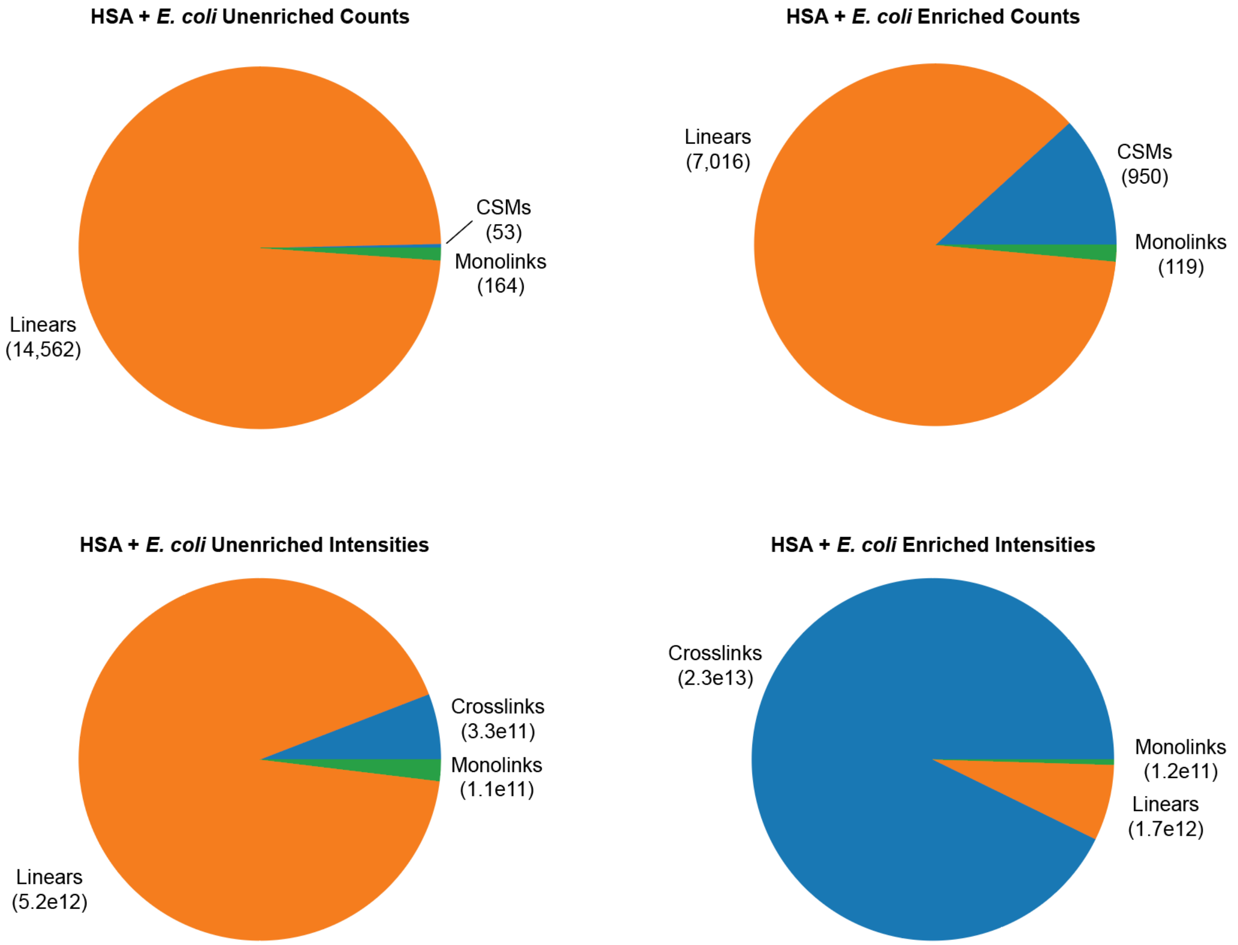

Supporting Figure 7. Linear and monolink peptide enrichment comparisons for HSA crosslinked with Alkyne-BVSC *in vitro* and mixed with E. coli peptides. Pie charts for unenriched (left) and enriched (right) peptide type counts (top) and intensities (bottom). Linears (orange), or unmodified peptides, are greatly reduced in count and intensity after enrichment when compared to monolinks (green) and to crosslinks (blue) at a 1% CSM FDR. Crosslink intensities were derived directly from the XiSearch output (*n=1*).

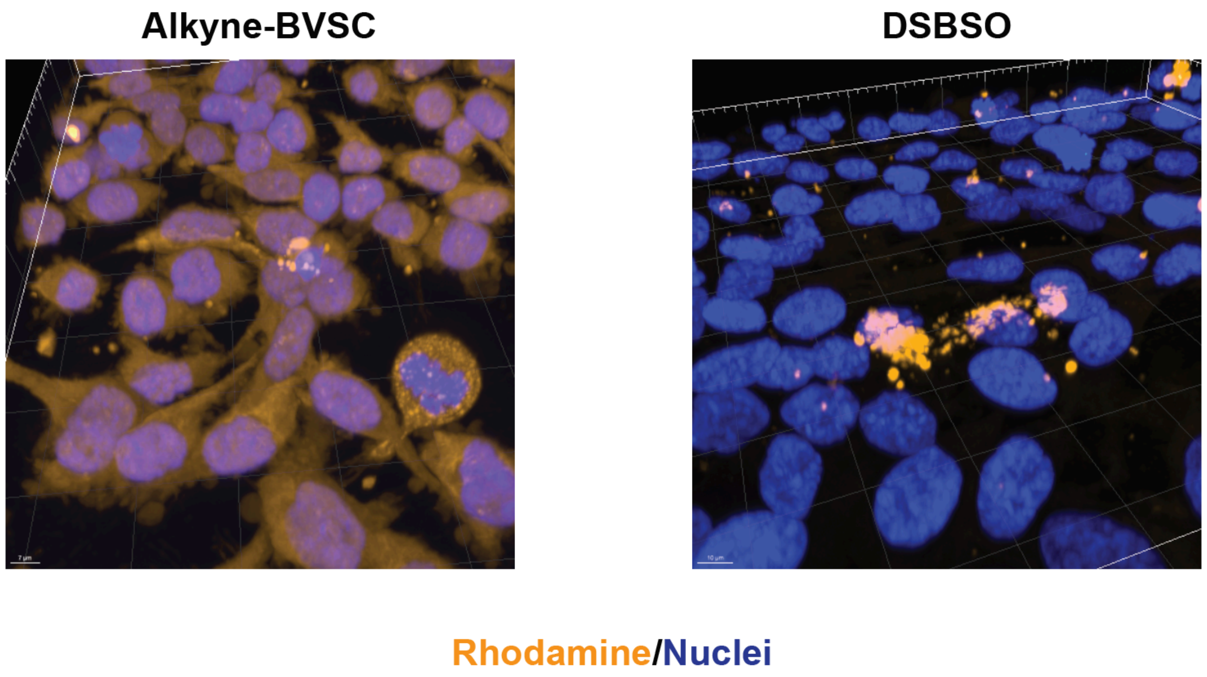

Supporting Figure 8. Live HeLa cells were incubated with 8mM of Alkyne-BVSC (left) or DSBSO (right), fixed with formaldehyde, permeabilized, clicked onto rhodamine, stained, and then imaged using confocal microscopy. A snapshot of a 3D image generated from a Z-stack of slices of cells is shown, with cell nuclei (DAPI, blue, 405nm) and crosslinking (rhodamine, orange, 561nm) visualized (*n=3*). Scale bars (bottom left) are displayed for reference (7µm for Alkyne-BVSC and 10µm for DSBSO).

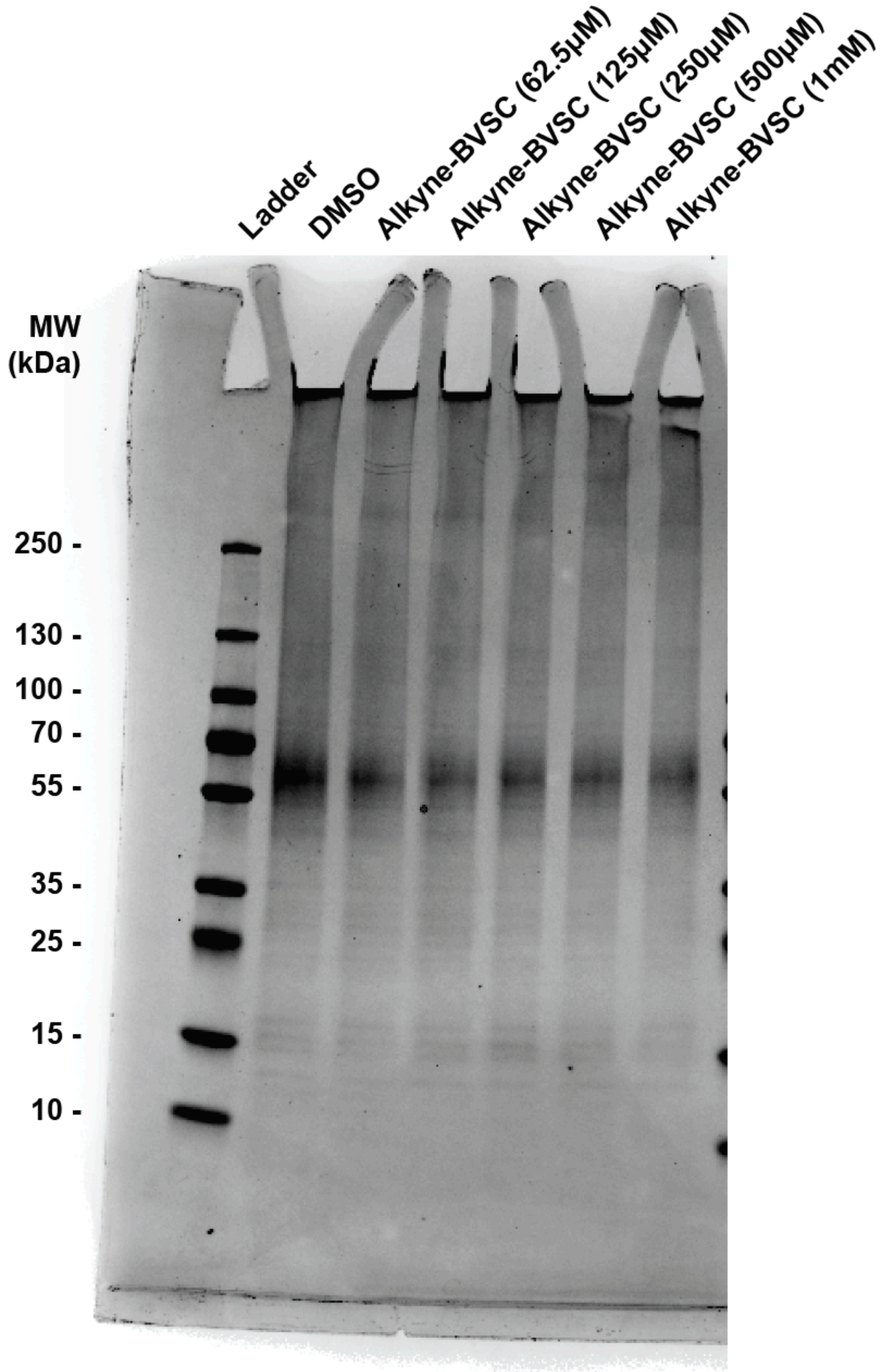

Supporting Figure 9. Coomassie stained gel for Alkyne-BVSC *in situ* crosslinked HeLa soluble protein fraction (*n=2*).

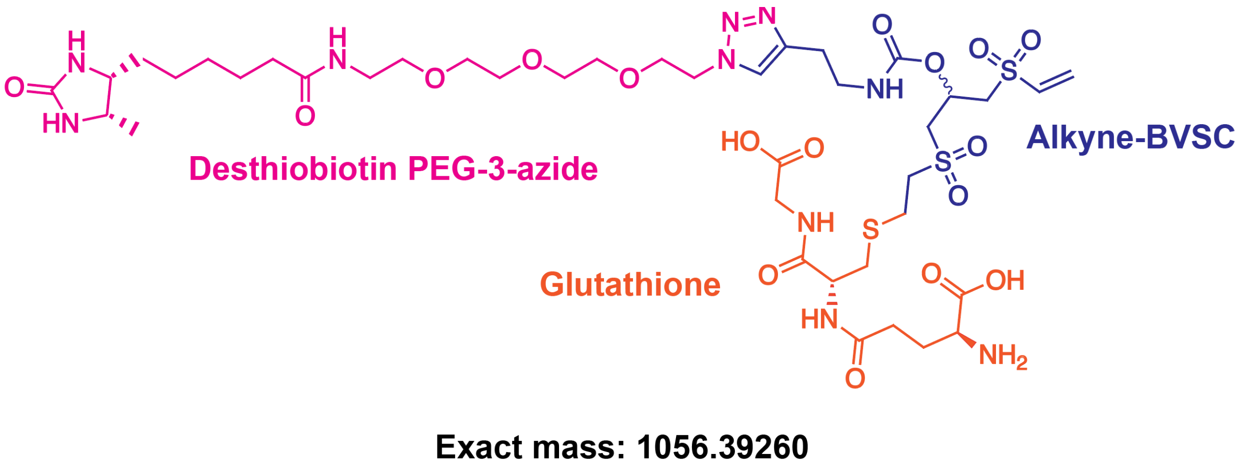

Supporting Figure 10. Alkyne-BVSC reacted with glutathione. Chemical structure of predicted Alkyne-BVSC crosslinker (blue) appended onto desthiobiotin (pink) via CuAAC and linked to glutathione (orange) via the free thiol group. This mass but linked to a cysteine (adjusted for the fixed added mass of carbamidomethylation) was also found with FragPipe's Open modification search (1056.39260 – 57.021464 = 999.371136).

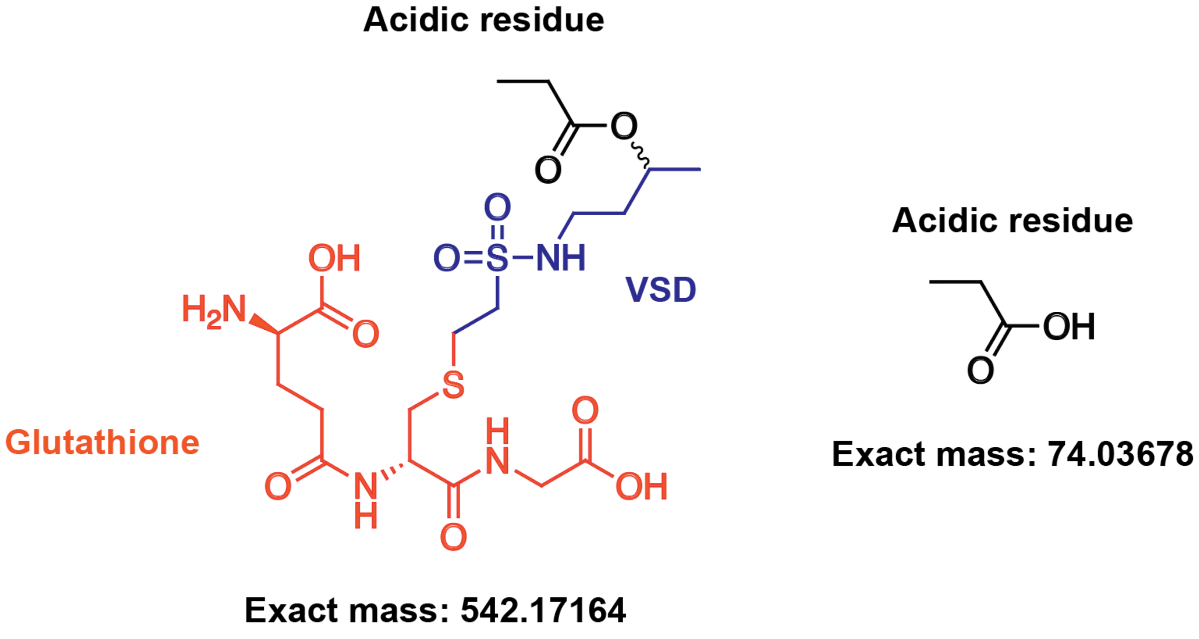

Supporting Figure 11. VSD monolink with glutathione reacted on the vinyl-sulfone end. Representation of the chemical structures for possible binding of VSD (blue) to an acidic residue (black, also right, simplified structure) on the diazirine end followed by glutathione (orange) binding on the vinyl sulfone end. The mass difference between the two species matches the MS1 mass found using FragPipe's Open modification search (542.17164 – 74.03678 = 468.13486).

I. Selected Annotated Spectra

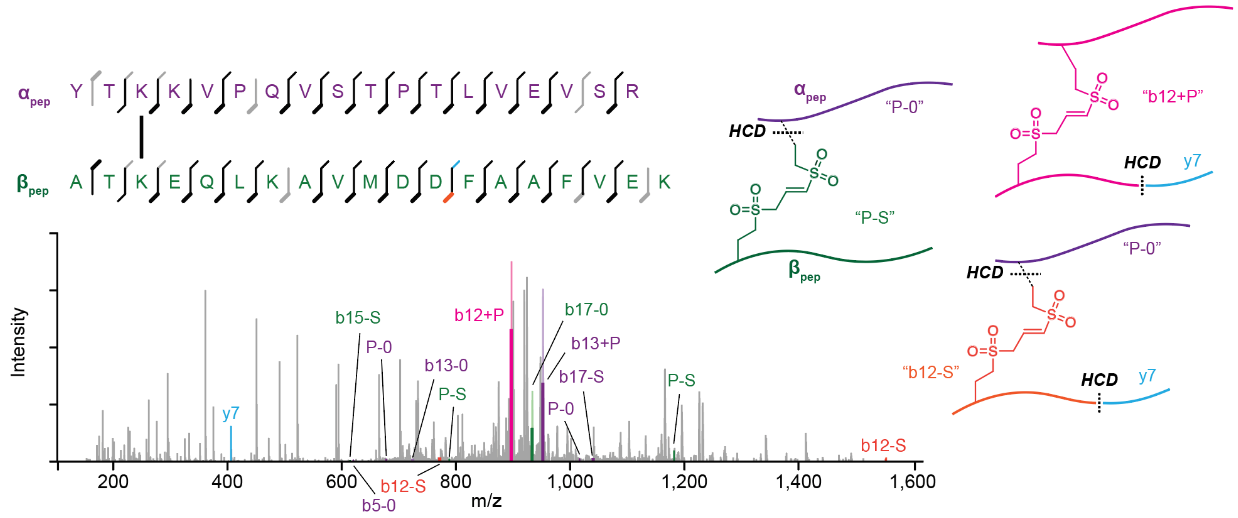

Example of a crosslinked spectrum match for Alkyne-BVSC. Partially annotated MS2 spectrum from XiView for an Alkyne-BVSC crosslink identified between HSA residues K437 and K565. Saturating peaks were removed and key peaks were highlighted for visualization purposes. Several different fragmentation possibilities are illustrated on the right. HCD cleavage (dashed black line) of the precursor ion results in generation of the alpha peptide (purple wavy line) alone ("P-0") along with the complementary beta peptide (green wavy line) with crosslinker ("P-S") that has been regenerated by the retro-Michael addition (green chemical structure) to reform the vinyl sulfone group. HCD can also fragment along the peptide bond, as shown with the "b12+P" (pink) and complementary y7 (cyan) fragment ions. Multiple HCD fragmentations can occur, resulting in more complex fragmentations, as illustrated by the bottom right depiction, where HCD fragments both the vinyl sulfone and along the beta peptide backbone, resulting in the alpha peptide ("P-0"), a fragment of the beta peptide with crosslinker ("b12-S"), and a beta peptide y-ion (y7, cyan).

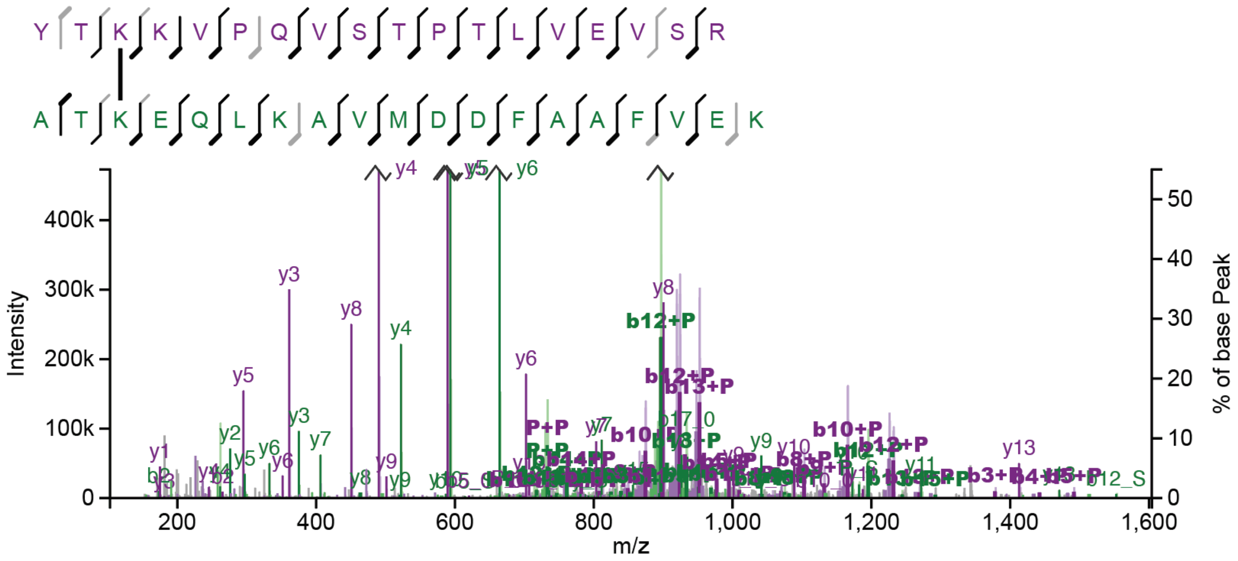

Original annotated spectrum for the partially annotated example. The dark dashes on the b- and y-ion fragments indicate that fragment is found with the crosslinker and the other peptide. Results are shown for b- and y-ions at a 5 ppm error with the crosslinker mass as a loss (240.0166, "S") and the peptide with the crosslinker cleaved by HCD (0, "0").

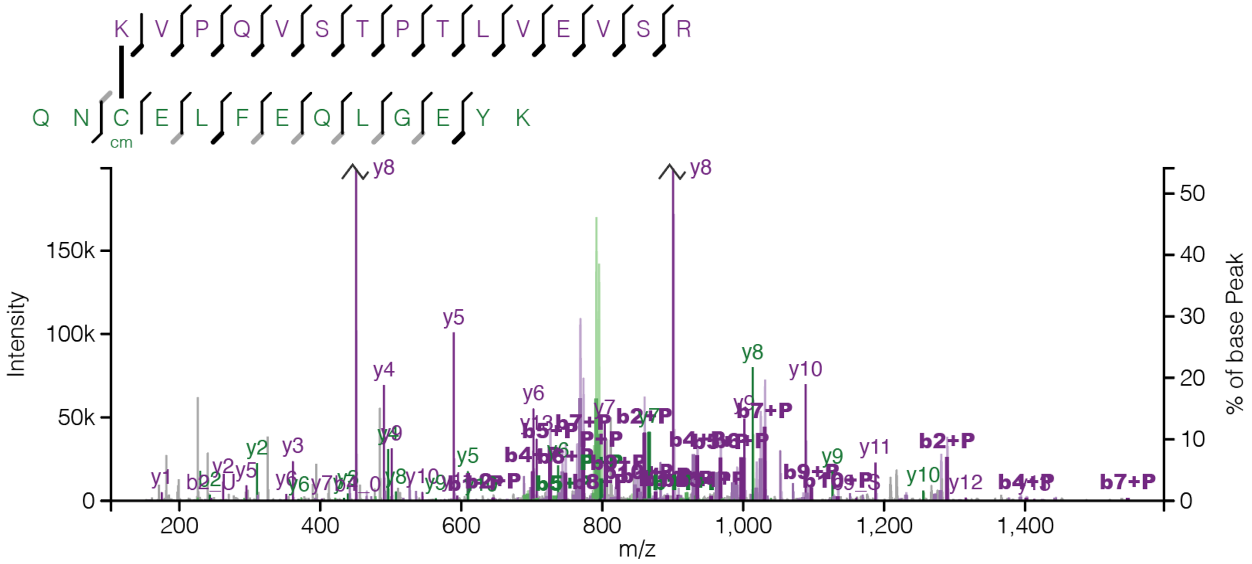

Alkyne-BVSC crosslinked spectrum match for HSA C416 to K438. Annotated spectrum for HSA crosslinked with Alkyne-BVSC between residues C416 and K438. The dark dashes on the b- and y-ion fragments indicate that fragment is found with the crosslinker and the other peptide. Results are shown for b- and y-ions at a 5 ppm error with the crosslinker mass as a loss (240.0166, "S") and the peptide with the crosslinker cleaved by HCD (0, "0").

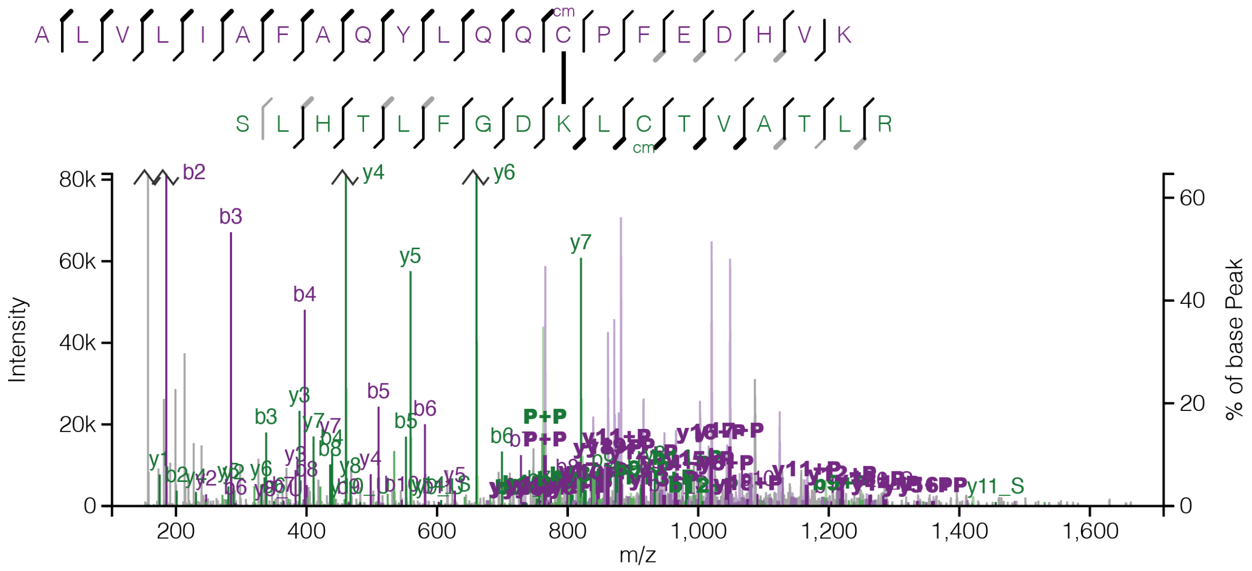

Top-scoring HSA Alkyne-BVSC crosslinked spectrum match. Annotated spectrum for HSA crosslinked with Alkyne-BVSC between residues C58 and K97. The dark dashes on the b- and y-ion fragments indicate that fragment is found with the crosslinker and the other peptide. Results are shown for b- and y-ions at a 5 ppm error with the crosslinker mass as a loss (240.0166, “S”) and the peptide with the crosslinker cleaved by HCD (0, “0”).

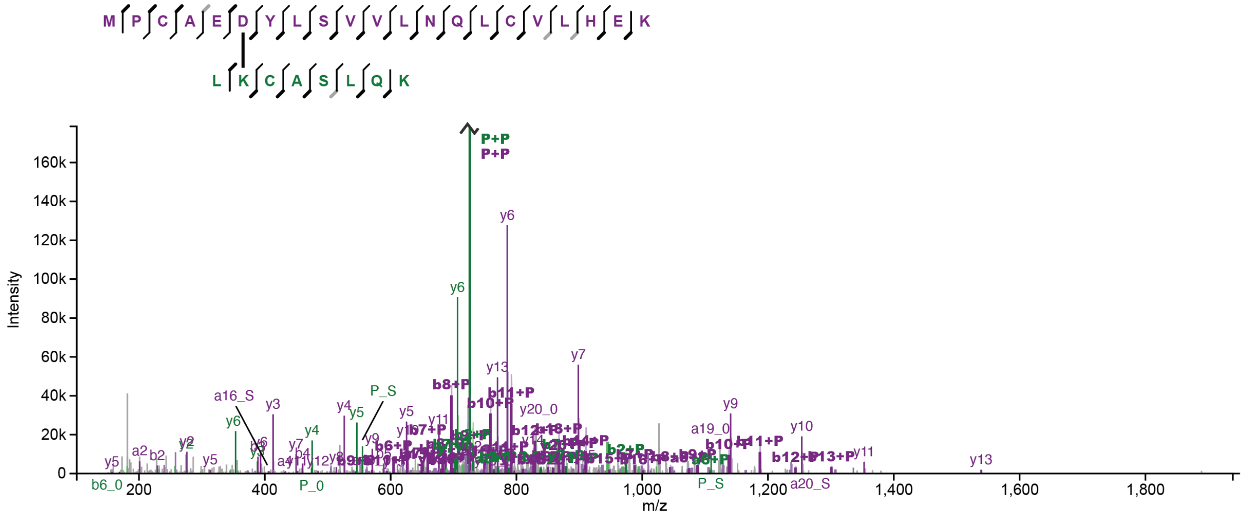

Example of an annotated HSA VSD crosslinked spectrum match. HSA crosslink between residues K223 and D475 from the VSD *in vitro* dataset. The dark dashes on the b- and y-ion fragments indicate that fragment is found with the crosslinker and the other peptide. Results are

shown for a-, b-, and y-ions at a 5 ppm error with the crosslinker mass as a loss (161.050964, “S”) and the peptide with the crosslinker cleaved by HCD (0, “0”). Both S and 0 stubs are found for both peptides, indicating MS-cleavability at both the vinyl-sulfone and diazirine ends of the crosslink.

II. Full-length Gels

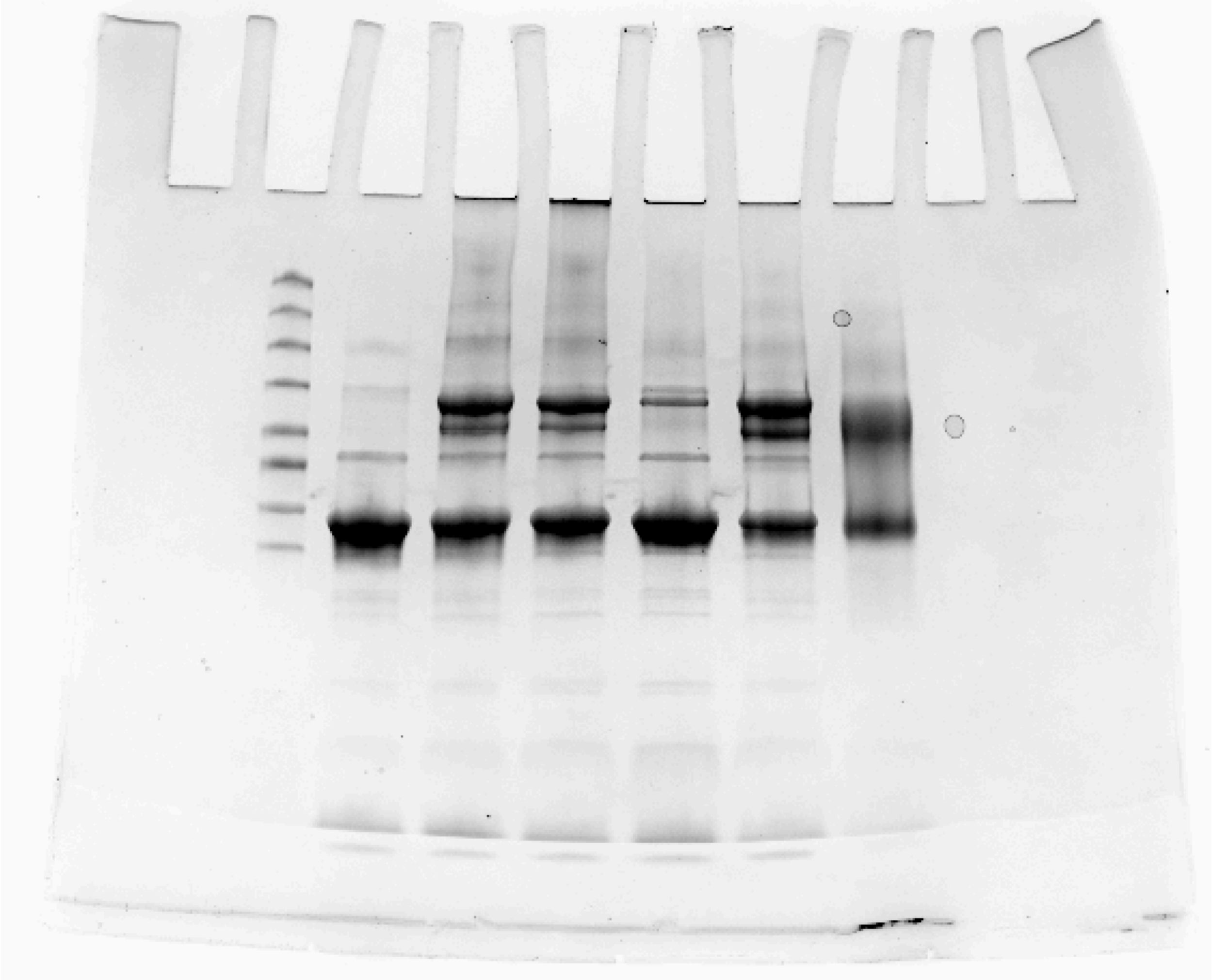

Full-length gel for Figure 1D.

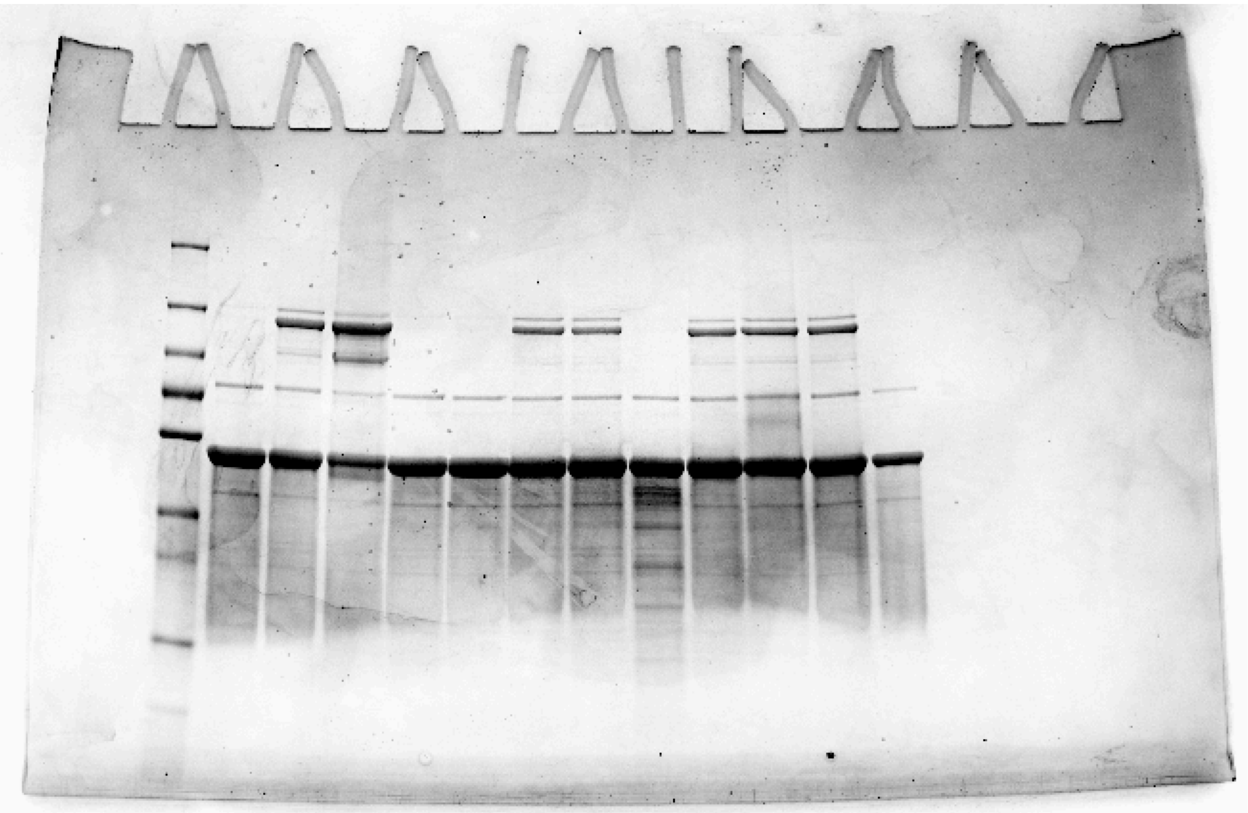
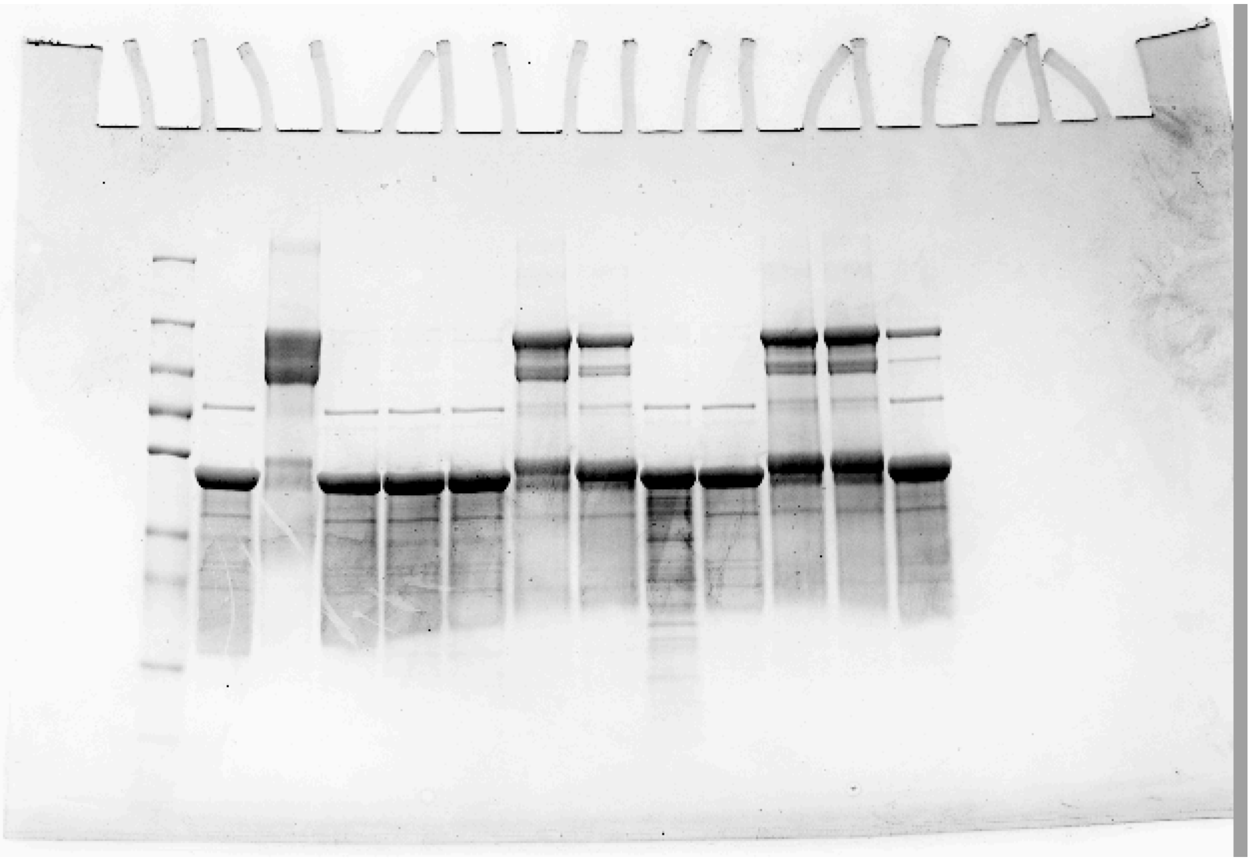

Full-length gels for Figure 1F.

B. Biochemical Methods

Materials

All solvents for mass spectrometry are LC-MS-grade unless otherwise stated: Water with 0.1% formic acid (Thermo, cat#85171), acetonitrile with 0.1% formic acid (ProteoChem, cat#LC6312-1L), acetonitrile ("ACN", Fisher Scientific, cat#AA47138M6), formic acid (Thermo, cat#85178), methanol (PerkinElmer, cat#N9304938). Other solvents are HPLC-grade unless otherwise stated: acetone (Sigma-Aldrich, cat#270725-4L), dimethylsulfoxide ("DMSO", anhydrous, Sigma-Aldrich, cat#276855-100ML), tert-butyl alcohol (ACS-grade, 99+%, Thermo, cat#AA33278AK). The following reagents were used: desthiobiotin PEG-3-azide (Sigma-Aldrich, cat#902020), tris(2-carboxyethyl)phosphine hydrochloride ("TCEP", Sigma-Aldrich, cat#C4706), tris[(1-benzyl-1H-1,2,3-triazol-4-yl)methyl]amine ("TBTA", Sigma-Aldrich, cat#678937), copper(II) sulfate pentahydrate ("$CuSO_4$", Sigma-Aldrich, cat#209198), TAMRA azide (Click Chemistry Tools, cat# AZ109-5), DL-dithiothreitol ("DTT", Sigma-Aldrich, cat#43815-5G), iodoacetamide ("IAA", Sigma-Aldrich, cat#I1149-5G), ammonium bicarbonate (Sigma-Aldrich, cat#A6141-1KG), urea (Sigma-Aldrich, cat#51456), phosphate-buffered saline (Research Products International Corp, cat#P32060-10000.0), human serum albumin (Sigma-Aldrich, cat#A9731), alpha-enolase (baker's yeast, Sigma-Aldrich, cat#E6126.2.5KU), trypsin (MS grade, Thermo, cat#90057), lys-c (MS-grade, Fukifilm Wako Chemicals, cat#125-05061), trifluoroacetic acid ("TFA", MS-grade, Fisher Scientific, cat#PI85183), SPE C18 disks (Empore, cat#66883-U), 4x Laemmli sample buffer (Bio-Rad, cat#1610747), beta-mercaptoethanol ("BME", Bio-rad, cat#1610710), 4–20% Mini-PROTEAN® TGX™ Precast Protein Gels (Bio-Rad, cat#4561096, 4561093, 4561094), 10x Tris/Glycine/SDS running buffer (Bio-Rad, cat#1610732), glutathione ("GSH", reduced form, Sigma-Aldrich, cat#G6529-25G), sodium hydroxide (Sigma-Aldrich, cat#S8045-500G), InstantBlue® Coomassie Protein Stain (Abcam, cat#ab119211), BCA protein assay kit (Thermo Fisher, cat#23225), cOmplete EDTA-free protease inhibitor cocktail (Sigma-Aldrich, cat# 5056489001), Dulbecco's Modified Eagle Medium ('DMEM', Thermo Fisher, cat#11995065), Fetal Bovine Serum ('FBS', Thermo Fisher, cat#26140079), DNase I (GoldBio, cat#D-300-100), lysozyme, egg white (GoldBio, cat#L-040-25), ultra centrifugal filter, 100kDa MWCO (Sigma-Aldrich, cat#UFC910008).

Escherichia coli (*E. coli*) was obtained from the ATCC (cat#700926) and cells were grown in RPI Luria Broth ("LB", Miller's LB Broth, Grainger, cat#31FZ62).

Chemical synthesis

Please see supporting methods.

Gel-based crosslinking assay

A 1 mg/mL solution of human serum albumin or alpha-enolase (baker's yeast) in phosphate-buffered saline (PBS) was incubated with 20x DMSO stocks of crosslinker for one hour at 37°C, or for 30 mins at RT for DSBSO, with shaking. For every 50uL of crosslinked solution, 6uL of a

master click-chemistry mix containing tetramethylrhodamine-azide, tris(2-carboxyethyl)phosphine hydrochloride (TCEP), tris[(1-benzyl-1H-1,2,3-triazol-4-yl)methyl]amine (TBTA), and copper (II) sulfate ($CuSO_4$) was added and reactions were incubated at RT for 1 hour with shaking as previously described[40]. Crosslinking and click reactions were quenched with addition of glutathione (5mM as a water stock) for 20 minutes at RT with shaking. A 4x Laemmli sample buffer (with beta-mercaptoethanol) was added to each sample, samples were boiled at 95°C for 5 mins, and samples were then cooled briefly and run on a 4-20% polyacrylamide gel at 150V for 40 minutes. Gels were imaged with a ChemiDoc Touch using the 565nm channel and subsequently incubated with InstantBlue Coomassie Protein Stain for at least 15 minutes at RT with rotation before being imaged using the Coomassie channel.

In vitro crosslinking protocol

Protein solutions were prepared in PBS at a concentration of 1mg/mL. Crosslinker stock solutions were made from dry compound stocks by resuspending in dry DMSO to give 20x working solutions. For the HSA crosslinking experiments, crosslinkers were made as 20mM DMSO stock solutions to give 1mM final concentrations. Protein solutions (in Eppendorf tubes) were flicked to mix and incubated at 37°C for one hour, or for 30 mins at RT for DSBSO, with shaking. Optionally, a 9x master CuAAC mix was generated using desthiobiotin PEG-3-azide as previously described[40] and this solution was added to the crosslinked protein solutions, solutions were vortexed to mix and then incubated at RT with shaking for one hour. Reactions were then optionally quenched with addition of glutathione (5mM final as a water stock), tubes were flicked to mix and incubated at RT for 20 mins. with shaking.

TFA cleavage assay

The gel-based crosslinking assay protocol was followed, except CuAAC was stopped by acetone protein precipitation at -20°C for at least one hour. Samples were then spun at 15,000xG at 4°C for 10 minutes and acetone was removed via pipette; samples were allowed to air-dry. Protein pellets were then resuspended in 50uL of listed concentrations of TFA at given temperatures (RT or 37°C) and timepoints (1, 2, or 24 hours). Acid-cleavage was quenched by addition of Laemmli sample buffer, sodium hydroxide was added to obtain a sample pH of ~7, and gels were loaded, run, and imaged as described in the gel-based crosslinking assay protocol.

In vitro PSMD2 pulldown crosslinking

Purified proteasome was obtained via PSMD2 (RPN1) pulldown as previously described[29]. The same *in vitro* crosslinking protocol was followed as above, except crosslinkers were treated at both 1mM and 250µM final concentrations, photoactivation of the relevant diazirine crosslinkers was accomplished as previously described[26], and no CuAAC was performed. For VSD, compound was incubated with eluates at 8, 16, and 24mM before UV-activating, quenching, and combining samples.

In-cell *E. coli* crosslinking

Escherichia coli (*E. coli*) were grown overnight in 5 mL of LB broth in 10mL falcon tubes at 37°C with shaking. 15 hours later, 10mL of cell growth was added to 1L of fresh LB in a 2L flask with an aerated top until cells reached an OD_{600} of 0.8. Cells were then spun at 4,000xG at 4°C for 15 minutes and supernatant was removed. The resulting cell pellet was weighed and resuspended in LB to achieve a concentration of 50mg/mL of cells in a 50mL conical. A 20x DMSO stock solution of Alkyne-BVSC (4mM final) was added and cells were rotated at 37°C for one hour. Crosslinking was quenched with addition of glutathione (50mM final as a water stock) with rotation at RT for 20 minutes. Sample was then spun at 4,000xG at 4°C for 10 minutes and the supernatant was poured off before pellets were snap-frozen in liquid nitrogen to store away at -80°C. The pellet was thawed on ice before resuspension in 50mL of cold PBS with protease inhibitor, DNase, and lysozyme. The cells were rotated at 4ºC for 20 minutes before being Dounce homogenized (5 passes of both course and fine). Sample was diluted with 50mL of cold PBS and subsequent lysis using an EmulsiFlex-C3 (Avestin, five passes at 15,000-20,000 psi). The resulting lysate was spun at 24,000xG at 4°C for 30 minutes to yield the supernatant, which was spun at 100,000xG at 4°C for one hour. Samples were concentrated using a 100,000 Dalton molecular weight cutoff spin column before incubation with a master CuAAC mix of desthiobiotin PEG-3-azide, TCEP, TBTA, and $CuSO_4$ for one hour at RT with shaking. Reaction was stopped and proteins were precipitated via addition of 4x the volume of ice-cold acetone.

In-cell HeLa crosslinking

HeLa cells were grown in T-175 flasks with 50mL of DMEM supplemented with 10% FBS to roughly 90% confluency. Media was aspirated and DMEM (20mL) that contained Alkyne-BVSC (8mM final as a 20x DMSO stock) was added to cells and flasks were gently swirled before incubation at 37ºC and 5% CO_2 for one hour. Media was then aspirated, cells were gently washed with cold PBS (2x10mL), scraped into conicals, and spun at 1,400xG for three minutes at 4ºC before the supernatant was aspirated. Cell pellets were snap-frozen using liquid nitrogen to store at -80ºC. Pellets were subsequently thawed on ice, resuspended in cold PBS with protease inhibitor, lysed via probe-tip sonication (3x1s pulses at 30% amplitude), and spun at 21,300xG for 30 minutes at 4°C to clarify the supernatant, which was clicked onto desthiobiotin and acetone precipitated to yield protein as described in the previous section.

Preparation of crosslinked peptides for LC-MS or size-exclusion chromatography

Proteins were crosslinked as described above, but instead of addition of sample buffer, proteins were precipitated in acetone at -20°C overnight. Pellets were then spun at 15,000xG at 4°C for 10 minutes and acetone was removed via pipette; samples were allowed to air-dry. For samples with large amounts of liquid, samples were spun at 4,000xG. Proteins were resuspended in an 8M/100mM urea/ammonium bicarbonate solution and a BCA assay was used to determine protein concentration; samples were subsequently diluted to 1-3 mg/mL. Proteins were then reduced with dithiothreitol (5mM) for 30 minutes at RT with shaking. Solutions were incubated with iodoacetamide (15mM) for 20 minutes at RT in the dark. Reactions were quenched with re-addition of DTT (5mM) and urea concentration was diluted to 1.6M using 100mM ammonium bicarbonate. Samples were incubated with trypsin (1:50 protease:protein) overnight at RT with shaking. For the HSA crosslinked samples, the Lys-C digest was skipped.

The following day, samples were either: A) acidified with TFA or formic acid (depending on the experiment) to pH 3 and a C18 stage-tip clean-up was performed or B) diluted with PBS and incubated with streptavidin beads for 1 hour at RT with rotation. For the latter, beads were pre-washed with PBS (3 x 10mL, 1,400xG spin for 1 min. at 4°C) and aliquoted as per manufacturer recommendation based on the amount of protein in the sample. Beads were washed with ammonium bicarbonate (25mM, 3 x 10mL, 1,400xG spin for 3 mins.) followed by sterile-filtered water (3 x 10mL, 1,400xG spin for 3 mins.). Beads were transferred to protein lo-bind tubes and liquid was removed after another spin. Beads were then incubated with 50% acetonitrile with 0.1% formic acid for 3 minutes at RT followed by spinning at 1,400xG spin for 3 mins. Supernatant was pipetted into a protein lo-bind tube this process was repeated twice. Samples were dried *in vacuo* before desalting with C18 stage tips to elution and then drying again *in vacuo*.

Stage tips[41] were made in-house using Empore SPE Disks by packing three C18 pieces on top of one another in a 200uL pipette tip. Stage tips were activated using 20uL of methanol, and the methanol was washed out twice using 20uL of 80% acetonitrile followed by 2 more washes of 0.1% TFA. All samples were loaded onto the stage tips and washed twice with 20uL of 0.1% TFA. To elute samples from still-wet stage-tips, two elutions of 10uL of 80% acetonitrile were performed. To elute samples from the dry stage tips, 10uL of methanol was pushed almost all the way through the stage tip followed by 2 x 10uL of 80% acetonitrile, with the first elution pushed almost all the way through and the second all the way to complete dryness. Eluted peptides were collected in protein lo-bind tubes and dried *in vacuo*. Stage tips were never allowed to go to full dryness unless storing washed, loaded sample at -20°C.

LC-MS data collection

Dried peptides were resuspended in 10uL of 1.6% acetonitrile with 0.1% formic acid, vortexed, and sonicated for 1 minute before injecting 1 ug of estimated peptide sample onto the Thermo Eclipse Orbitrap coupled to a Vanquish Neo HPLC system. Peptides were ionized using an EASY-Spray source and eluted over an EASY-Spray PepMap Neo 75um x 500mm C18 column (heated to 40°C) with LC-MS quality water or acetonitrile containing 0.1% formic acid (mobile A and B, respectively) with the following gradients (%B): early peptide size-exclusion samples: 300nL/min flow rate, 0-1 min. (1.6%), 1-10 min. (1.6 to 17.6%), 10-87 min. (17.6 to 32.0%), 87-92.5 min. (32.0 to 44.0%), 92.5-95 min. (44.0 to 76.0%), 400 nL/min flow rate, 95-100 min. (76.0%); middle peptide size-exclusion samples: 300nL/min flow rate, 0-1 min. (1.6%), 1-10 min. (1.6 to 10.4%), 10-87 min. (10.4 to 32.8%), 87-92.5 min. (32.8 to 44.0%), 92.5-95 min. (44.0 to 76.0%), 400 nL/min flow rate, 95-100 min. (76.0%); late peptide size-exclusion samples: 300nL/min flow rate, 0-1 min. (1.6%), 1-10 min. (1.6 to 7.2%), 10-87 min. (7.2 to 25.6%), 87-92.5 min. (25.6 to 44.0%), 92.5-95 min. (44.0 to 76.0%), 400 nL/min flow rate, 95-100 min. (76.0%). Note that the first biological replicate for the HSA HCD dose experiment was performed with the above gradient but using an UltiMate 3000 RSLC nano LC system with an Acclaim™ PepMap™ 100 C18 3µm, 75µm x 2cm trap column and with the analytical column heated to 50°C.

Peptides were analyzed using the following MS global parameters: method duration of 85 minutes, infusion mode – liquid chromatography, expected LC peak widths – 30s, advanced peak determination checked, default charge state of 2, EASY-IC internal mass calibration, NSI ion source, static spray voltage at 2000V in positive mode, static gas mode with a sweep gas setting

of 2 and ITT temperature of 280°C. Samples were collected using the following shared scan parameters: Duty-cycle of 3 seconds, MS-OT at 240k resolution, normal mass range, quadrupole isolation checked, scan range of 380-2000, RF lens of 35%, a custom AGC target of 150% with a max injection time of 100ms, 1 microscan in profile mode at positive polarity with a source fragmentation of 10V, EASY-IC checked; subbranch MIPS – peptide; subbranch intensity – 2.5e4, subbranch charge state 3-7, subbranch dynamic exclusion of 1 time after 30 seconds with a mass tolerance of 10 ppm, exclude isotopes and dependent scan on single charge state checked; subbranch priority 1: subbranch A: charge state – 4, precursor selection range – 380-1800, subbranch B: charge state – 5, precursor selection range – 380-1350, subbranch C: charge state – 6-7, precursor selection range – 380-1000; rejoined subbranch: sort by intensity – most intense, subbranch ddMS2 OT HCD: isolation mode – quadrupole with a window of 1.4 m/z, stepped HCD (normalized) of 18, 26, and 30, detector type – orbitrap at 60k resolution from 150-2000 m/z, AGC target of 750% with a max injection time of 150ms, 1 microscan, centroid data. Subbranch priority 2: subbranch: charge state – 3, precursor selection range – 380-2000, subbranch: sort by intensity – most intense, subbranch ddMS2 OT HCD: isolation mode – quadrupole with a window of 1.4 m/z, stepped HCD (normalized) of 18, 26, and 30, detector type – orbitrap at 60k resolution from 150-2000 m/z, AGC target of 750% with a max injection time of 150ms, 1 microscan, centroid data. For the HCD energy dose-response HSA experiment, the lowest of the three HCD energies was changed to 9, 12, 15, 18, or 21, while the higher two energies remained the same and a 95-minute method duration was used. For DSBSO crosslinked samples, we used a stepped HCD of 20, 26, and 30 with the rest of the method being the same; these energies closely mirror what has been used previously[42]. For the VSD and SDA experiments, the above standard method was used, but with a stepped HCD with NCEs of 20, 26, and 30, an MS2 max injection time of 250ms, and RunStart Easy-IC internal mass calibration. For the RPN1 experiments, a second injection of each fraction was performed using the relevant base method, except the scan and precursor selection ranges were narrowed to 650-905 m/z.

The HSA HCD dose-response crosslinking datasets were acquired with the above methods, adjusted to accommodate an UltiMate 3000 RSLC nano LC system with an Acclaim™ PepMap™ 100 C18 3µm, 75µm x 2cm trap column.

Peptide size-exclusion chromatography

Dried peptides were resuspended in 25uL of 30% acetonitrile with 0.1% TFA, vortexed, and sonicated for 1 minute before injecting onto a Superdex 30 Increase 3.2/300 column (Cytiva, cat#29219758) with a 20uL injection loop using an Äkta pure micro system and eluting peptides using the following gradient: 0-0.03 mL (0.010 µL/min), 0.03-0.50 mL (0.010 µL/min), 0.50-0.95 mL (0.015 µL/min), 0.95-1.45 mL (0.030 µL/min), 1.45-1.85 mL (0.040 µL/min), 1.85-3 mL (0.040 µL/min). Crosslinked peptides eluted over the range 1.00-1.45 mL as 50uL fractions pipetted into protein lo-bind tubes.

Crosslinked peptide identification workflow

Raw files were processed using the preprocessing workflow described by the Rappsilber lab (https://github.com/Rappsilber-Laboratory/preprocessing). xiSEARCH (https://github.com/Rappsilber-Laboratory/XiSearch, v. 1.8)[25] was used to perform the

crosslinked peptide search. Recalibrated mgfs were searched against a fasta file of the select protein using the following global parameters: methionine oxidation (variable, 147.035395), cysteine carbamidomethylation (fixed, 160.03065, "Ccm"), b and y ions, water loss (S/T/D/E/c-term, 18.01056027), ammonia loss (R/K/N/Q/*N*-terminus, 17.02654493), methionine hydroxylation loss (63/99828547), digestion after K or R except when followed by P (Trypsin), 3 missed cleavages allowed, non-covalent search, MS1 precursor tolerance of 2.0 ppm, MS2 fragment tolerance of 5.0 ppm, linears evaluated, PeptideIon enabled, 3 conservative losses, missing monoisotopic automated match, 10 peaks to consider for alpha peptide, 3 peptide modifications max, 20 modified peptides per peptide match, fragment tree FU, 2 missing isotope peaks, and minimum charge of 3. The 'write peak annotations' box was selected. For Alkyne-BVSC, the following search parameters were added: monolink (variable, K/H/*N*-terminus, 222.0016), cleavable crosslinker mass (0, name: 0; 222.0016, name: S), crosslinker mass (222.0016, K/H/*N*-terminus to K/H/*N*-terminus), crosslinker mass (164.980136, Ccm to K/H/*N*-terminus), crosslinker mass (107.958672, Ccm to Ccm). For BVSS-Me/BVSS-Et/BVSS-Ph, the following search parameters were added: monolink (variable, K/H/*N*-terminus, 240.0166), cleavable crosslinker mass (0, name: 0; 240.0166, name: S), crosslinker mass (240.0166, K/H/*N*-terminus to K/H/N-terminus), crosslinker mass (182.995136, Ccm to K/H/*N*-terminus). For *in situ* experiments, the monolink variable modification was removed. For Alkyne-A-DSBSO, the following search parameters were added: cleavable crosslinker mass (0, name: 0; 54.0106, name: A; 85.9824, name: B; 158.0038, name: S), crosslinker mass (158.0038, K/*N*-terminus to K/*N*-terminus) as previously described[42]. For VSD, the following search parameters were added: glutathione quench (variable, D/E, 468.1352), cleavable crosslinker mass (0, name: 0; 161.050964, name: S), crosslinker mass (161.050964, K/H/*N*-terminus to any), crosslinker mass (104.0295, Ccm to any), crosslinker mass (47.008036, Ccm to Ccm).

For Alkyne-BVSC with CuAAC but without TFA cleavage, the following search parameters were added to the base Alkyne-BVSC config: deamidation (variable, N/Q, 0.984016), pyroglutamate (variable, Q (peptide *N*-terminus only), glutathione-quench (variable, K/H/*N*-terminus, 529.08586), uncleaved glutathione-quench (variable, K/H/*N*-terminus, 1056.392), uncleaved glutathione-quench (variable, C, 999.3724), cleavable crosslinker mass (749.30880, name: U), crosslinker mass (749.30880, K/H/*N*-terminus to K/H/N-terminus), crosslinker mass (692.287336, Ccm to K/H/*N*-terminus), crosslinker mass (635.265872, Ccm to Ccm).

For post-search filtering: for CF filtering, the same filters were applied post-search as previously described[26]. For stub-based filtering, we used three different methods: method A – require a P-S on both peptides, method B – method A in addition to allowing for one P-S on one peptide and 3 S stubs on the other peptide, and method C – method B in addition to allowing for 5 S stubs on both peptides. See **Figure 3** for an illustration of these filters. Custom Python scripts were used to achieve these filters and are available upon request. The non-acid-cleaved crosslinker on the full peptide (P-U) or fragmented peptides (U) were also allowed for these filters. The resulting file that contained a list of these matches was then used to filter the original xiSEARCH csv file output.

The Python scripts used to achieve this filtering work as follows: the first script filters the unzipped peak tsv annotations file from xiSEARCH for instances of column 13 ('FragmentName') that contain the text "_S" and instances of column 21 ('Description') that contain the text "crosslinked". The output of the first script is then filtered by a subsequent script for whether the

combination of scan (column 2, 'ScanNumber') and peptide type ("alpha" or "beta" denoting the alpha or beta peptide match, column 9, 'MatchedPeptide') contains "P_S" or "_S" as text in column 13. If both the alpha and beta peptide for that scan contain "P_S" in any of the rows for column 13, or if one of the alpha or beta peptides contains "P_S" and the other contains three text instances of "_S", or if both the alpha and beta peptides for that scan contain at least five instances of "_S", the entire row is written into a new tsv file. Finally, this resulting tsv file is used to filter for scans from the csv xiSEARCH output file. The "S" in "P_S" and "_S" is set in the xiSEARCH configuration as a cleavable crosslinker loss ("loss:CleavableCrossLinkerPeptide:MASS:222.0016;NAME:S"), so this letter is customizable, and more letters can be added for filtering for additional cleavable crosslinker losses. For example, we also searched for "U" the exact same way as "S" for experiments where we appended on desthiobiotin in the above scripts ("loss:CleavableCrossLinkerPeptide:MASS:749.30880;NAME:U").

The filtered csv was imported along with the xiSEARCH config file and the fasta into XiFDR (https://github.com/Rappsilber-Laboratory/xiFDR, v. 2.2.1) and run with the following parameters for setting a 1% residue-pair FDR: complete FDR selected, PSM: 100, Peptide Pair: 100, Protein Group: 100, Residue Pairs: 1, Protein Pairs: 100, min. pep. length of 5, no consecutive selected, boost separately selected, boost selected: residue pairs with 4 steps. For RPN1 experiments, the above parameters were changed: Residue Pairs: 5, min. pep. Length of 6, no consecutive not selected, 'betweens' selected. For setting a 1% PSM FDR, the following parameters were set: PSM: 1, Peptide Pair: 100, Protein Group: 100, Residue Pairs: 100, Protein Pairs: 100. Boosting on 'betweens' was performed optionally, where noted. The resulting mzid and csv files were exported and the CSM, mgfs, and fasta files were uploaded to XiView[43] (https://github.com/Rappsilber-Laboratory/xiview) to visualize crosslinking data.

Open modification search

FragPipe (https://github.com/Nesvilab/FragPipe, v. 22) was used in conjunction with MSFragger (v. 4.1), IonQuant (v. 1.10.27), diaTracer (v. 1.1.5), DIA-NN (v. 1.8.2_beta_8), and Python (v. 3.9.13)[22, 44-46]. 'Open' workflow[21] was loaded and run against selected raw files using the selected fasta with reverse sequence(s) incorporated by FragPipe. Two missed cleavages were allowed ('stricttrypsin', KR) with a precursor mass tolerance of -150-500 Da and a fragment mass tolerance of 20 ppm with a 1% FDR at both the peptide and protein level. For the Alkyne-BVSC *in situ* Open modification search, a custom fasta was generated based on the top 300 HeLa proteins by intensity rank as described in 'FASTA file generation' with decoys added using FragPipe's built-in feature. Additionally, a precursor mass tolerance of -150-1,000 Da was utilized.

FASTA file generation

The FASTA file used for human serum albumin (P02768, download date: August 2, 2023) was downloaded from UniProt. For experimental samples, protein identifications were generated using the FragPipe 'HeLa' workflow with MS1 Quant enabled (Top 3 N) to search against raw files from the unenriched protein sample using the human (taxonomy ID: 9606, Reviewed entries only, download date: March 3, 2025, size: 20,430) or *E. coli* proteome (taxonomy ID: 83333, Reviewed entries only, download date: April 15, 2024, size: 6,066), both downloaded from UniProt. Proteins

were then filtered for at least $1x10^8$ (HeLa, 1,998 proteins or *E. coli*, 1,919 proteins) or $5x10^8$ intensity (proteasome, human, 202 proteins).

Confocal microscopy

HeLa cells were seeded at 10,000 cells per well and grown on 8-well dishes (Ibidi, cat#80826) in 300uL of DMEM (Life Technologies, cat#11995065) supplemented with 10% FBS (Thermo, cat#A3382001) and L-glutamine (ATCC, cat#30-2214) for 48 hours at 37°C, 5% CO2. For cell treatments, media was aspirated and replaced with 300uL of fresh DMEM containing chemical crosslinker at noted concentrations. Dishes were gently mixed and incubated for one hour at 37°C, 5% CO2. The media was aspirated and cells were washed with 300uL of PBS once before being incubated with formaldehyde (4% wt/v in PBS made from commercial stock, Sigma-Aldrich, cat#252549) for 20 mins. under a chemical hood. Cells were then washed with PBS with gentle shaking for 3 mins. twice before being incubated with 0.2% Triton X-100 in PBS for 5 mins. with shaking at RT. The PBS washes were repeated and rhodamine was appended onto crosslinked protein by incubating CuAAC mix with cells for one hour at RT with gentle shaking. The PBS washes were repeated and cells were incubated with a 1:1000 DAPI solution (Thermo, cat#62248) in PBS for one hour at 37°C with gentle shaking. PBS washes were repeated and cells were stored in PBS at 4°C in the dark until ready to image.

Confocal microscopy was performed using an Andor spinning disk confocal on Leica DMi8 base with a 63x water objective lens. Images were captured via a Z-stack of 22µm with an automatic number of steps for each of the following excitation wavelengths (in order): 561nm, 488nm, and 405nm. Exposure times and laser powers were adjusted based on the sample set.

Custom ImageJ (Fiji) macros were used to process the images to quantify rhodamine intensity normalized to the number of cells in the given image. Custom python scripts were written to combine outputted csv files and to generate plot summaries of the corresponding data. Imaris Viewer was used to generate the 3D images.

Data Availability

The HSA HCD dose-response crosslinking data can be accessed via PRIDE (PXD065869). The Alkyne-BVSC enriched HSA crosslinking data can be accessed via PRIDE (PXD065859). The Alkyne-BVSC proteasome crosslinking data can be accessed via PRIDE (PXD065858). The DSSO proteasome crosslinking data can be accessed via PRIDE (PXD065912). The VSD HSA crosslinking data can be accessed via PRIDE (PXD065870). The VSD proteasome crosslinking data can be accessed via PRIDE (PXD065871). The SDA proteasome crosslinking data can be accessed via PRIDE (PXD065946). The DSBSO enriched HSA proteomics data can be accessed via PRIDE (PXD065949).

Experimental Design and Statistical Rationale

We have performed two independent biological replicates for all experiments, unless otherwise stated. Crosslinking MS experiments can vary greatly, even within samples, as identification of ions to undergo MS2 is highly stochastic, especially considering the stoichiometries of crosslinked

peptides within typical proteomics samples. For many of these experiments, only one biological replicate was performed; however, as we do not make any claims about new biology, we emphasize that our results are appropriate for the constraints of the experiments.

C. Chemical Methods

I. General. Bis(vinylsulfonyl)propanol was purchased from Combi-Block, Inc.(San Diego, CA). All other chemicals were purchased from Sigma-Aldrich, Inc. (Milwaukee, WI). HPLC was performed on an Agilent 1200 series instrument equipped with a multi-wavelength detector and Agilent 385-ELSD detector. The ^{1}H NMR and the ^{13}C NMR spectra were recorded on a Varian 400 MHz spectrometer operating at 400 MHz and 100 MHz respectively. Chemical shifts are reported in part per million (δ) and referenced internally to deuterated solvents. Mass spectroscopy data were acquired on an Agilent single quadrupole LC/MSD XT mass spectrometer.

II. Synthetic Schema

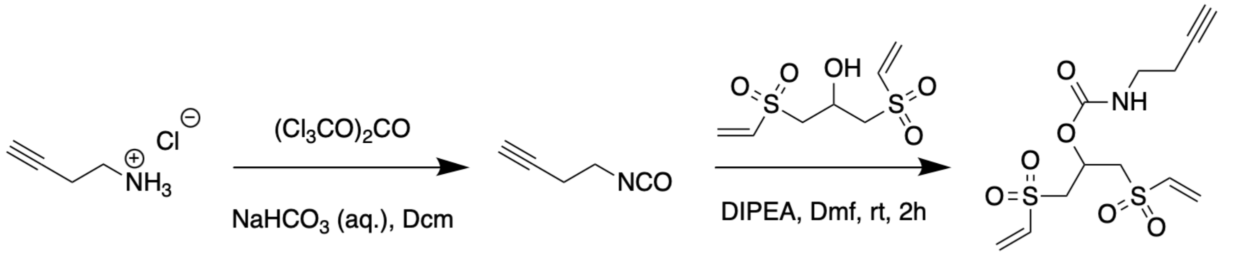

Alkyne-BVSC

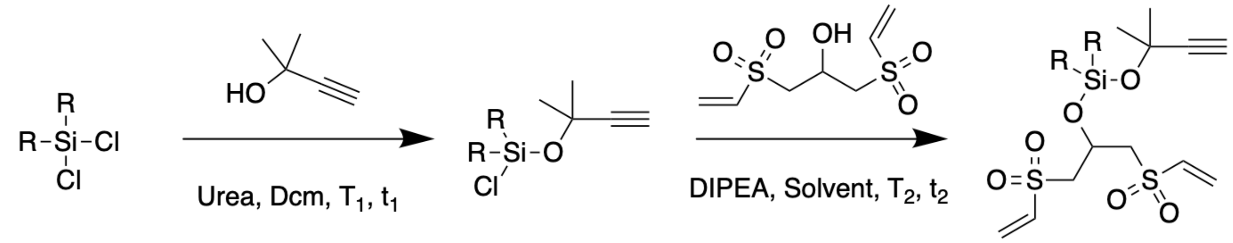

BVSS-Me: R = Me, T_1 = 0°C, t_1 = 1h; T_2 = microwave 60°C, t_2 = 30min, solvent = Dcm; 66%.

BVSS-Et: R = Et, T_1 = rt, t_1 = 2h; T_2 = microwave 70°C, t_2 = 2h, solvent = THF; 15%.

BVSS-Ph: R = Ph, T_1 = rt, t_1 = 1h; T_2 = microwave 80°C, t_2 = 72h, solvent = 1,2-dichloroethane; 40%.

1, 3-bis(vinylsulfonyl)propan-2-yl but-3-yn-1-ylcarbamate (Alkyne-BVSC)

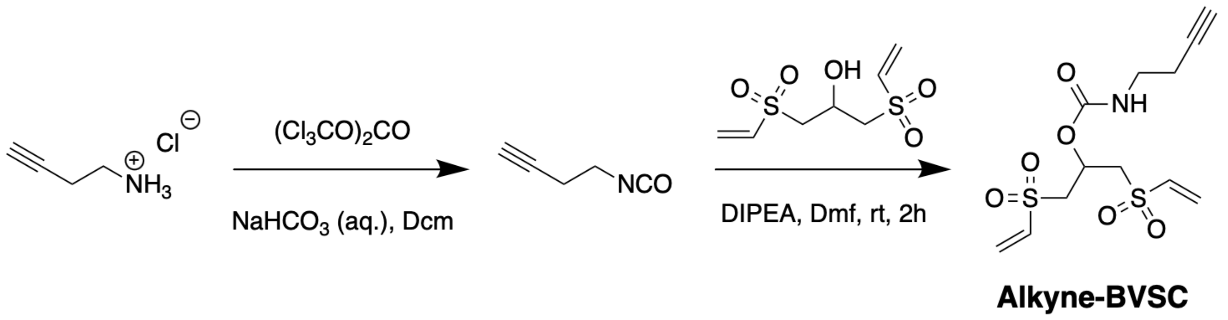

4-isocyanatobut-1-yne. [Reference: Org. Syn., Coll. Vol. 10, p.544 (2004); Vol. 78, p.220 (2002).] A 300-mL round-bottomed flask was charged with dichloromethane (100 mL), saturated aqueous sodium bicarbonate (100 mL), and but-3-yn-1-amine hydrochloric acid (2.69 g, 25.5 mmol). The biphasic mixture is cooled in an ice bath and vigorously stirred with a magnetic bar while triphosgene (2.52 g, 8.42 mmol) was added in a single portion. The reaction mixture was stirred in the ice bath for 15 min, additional 34 mL saturated sodium bicarbonate was added until pH 7 to destroy excess triphosgene, and then poured into a 250-mL separatory funnel. The organic layer was collected, and the aqueous layer was extracted with methylene chloride (3 × 15 mL). The combined organic layers were washed with HCl (15 mL, 1M) and then dried ($MgSO_4$), filtered, and concentrated at reduced pressure (caution: product is volatile) to give a colorless liquid which was used without further purification. 1.4 g, 58 % in yield. ^1H NMR (400 MHz, DMSO) δ 3.47 (t, J = 6.3 Hz, 2H), 3.04 (t, J = 2.6 Hz, 1H), 2.57 (td, J = 6.3, 2.6 Hz, 2H). ^{13}C NMR (101 MHz, DMSO-d6) δ 125.13, 82.08, 74.32, 42.38, 21.64.

1,3-Bis(vinylsulfonyl)propan-2-yl but-3-yn-1-ylcarbamate (Alkyne-BVSC). To a solution of 1,3-bis (vinylsulfonyl) propan-2-ol (1.2 g, 5 mmol) in DMF (5 mL) was added 4-isocyanatobut-1-yne (0.76 g, 8 mmol) followed by DIPEA (0.65 g, 0.87 mL, 5 mmol), the mixture was then stirred at rt for 2h. The mixture was diluted with dichloromethane and washed with aq. NH_4Cl, brine and then dried over $MgSO_4$. Removal of solvent gave 2.1 g yellow oil. The crude product was further purified by flash chromatography on a silica column eluted with 20-90% ethyl acetate in hexanes. Removal of solvent gave an off-white solid. 1.33 g. 80 % in yield. ^1H NMR (400 MHz, $CDCl_3$) δ 6.70 (dd, J = 16.5, 9.8 Hz, 2H), 6.46 (d, J = 16.6 Hz, 2H), 6.23 (d, J = 9.8 Hz, 2H), 5.51 – 5.40 (m, 1H), 5.32 (s, 1H), 3.55 (dd, J = 5.7, 4.1 Hz, 4H), 3.38 – 3.27 (m, 2H), 2.40 (td, J = 6.3, 3.0 Hz, 2H), 2.03 (t, J = 2.6 Hz, 1H). ^{13}C NMR (101 MHz, $CDCl_3$) δ 154.36, 136.56, 131.52, 81.22, 70.58, 64.30, 56.67, 39.95, 19.85. MS: $C_{12}H_{17}NO_6S_2$ calculated: 335.05, found: 336.0 (MH^+).

((1,3-bis(vinylsulfonyl)propan-2-yl)oxy)dimethyl((2-methylbut-3-yn-2-yl)oxy)silane (BVSS-Me)

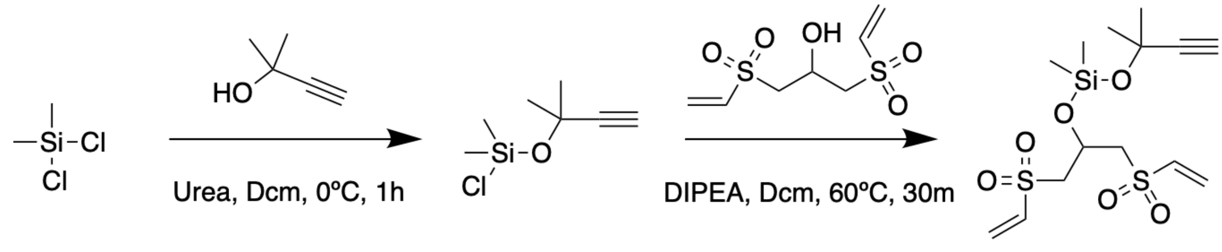

BVSS-Me-S1 **BVSS-Me**

Chlorodimethyl((2-methylbut-3-yn-2-yl)oxy)silane (BVSS-Me-S1). Dichlorodimethylsilane (12.9 g, 100 mmol, 1 Eq) and urea (7.2g, 120 mmol, 1.2 Eq) were stirred in anhydrous dichloromethane (10 mL) at rt for 15 min, and then 2-methylbut-3-yn-2-ol (8.41 g, 100 mmol, 1 Eq) was slowly added. The mixture was stirred at rt for 4 h and then filtered. The filtrate was dried by $CaCl_2$ and then isolated by fractional distillation under reduced pressure to give a colorless oil. 11.8 g. 67 % in yield. ^{1}H NMR (400 MHz, $CDCl_3$) δ 2.49 (s, 1H), 1.58 (s, 6H), 0.54 (s, 6H). ^{13}C NMR (101 MHz, $CDCl_3$) δ 72.10, 68.59, 32.92, 32.66, 4.43.

((1,3-bis(vinylsulfonyl)propan-2-yl)oxy)dimethyl((2-methylbut-3-yn-2-yl)oxy)silane (BVSS-Me). Chlorodimethyl ((2-methylbut-3-yn-2-yl)oxy) silane (1.94 g, 1.1 Eq, 11.0 mmol) and 1,3-bis (vinylsulfonyl) propan-2-ol (2.40 g, 1 Eq, 10.0 mmol) were stirred in DCM (8 mL), and then DIPEA (1.42 g, 1.1 Eg, 11 mmol) was added. *The mixture was heated under microwave* at 60°C for 30 min, a clear solution formed. The mixture was washed with 0.1 N HCl (2 × 5 mL), brine (5 mL) and then dried over MgSO4. Solvent was evaporated to give a colorless oil. 2.51 g, 66 % in yield. Further purification by flash chromatography on silica eluted with ethyl acetate in hexanes at 0-100% gradient to give a colorless oil. 1.63 g, 43 % in yield and 97 % in purity. ^{1}H NMR (400 MHz, $cdcl_3$) δ 6.71 (dd, J = 16.6, 9.9 Hz, 2H), 6.42 (d, J = 16.6 Hz, 2H), 6.13 (d, J = 9.9 Hz, 2H), 4.85 (t, 1H), 3.58 (dd, J = 14.7, 5.8 Hz, 2H), 3.47 (dd, J = 14.7, 5.4 Hz, 2H), 2.50 (s, 1H), 1.52 (s, 6H), 0.29 (s, 6H). ^{13}C NMR (101 MHz, $cdcl_3$) δ 137.86, 129.83, 88.11, 72.01, 67.06, 62.90, 59.47, 32.84, 0.00.

((1,3-bis(vinylsulfonyl)propan-2-yl)oxy)diethyl((2-methylbut-3-yn-2-yl)oxy)silane (BVSS-Et)

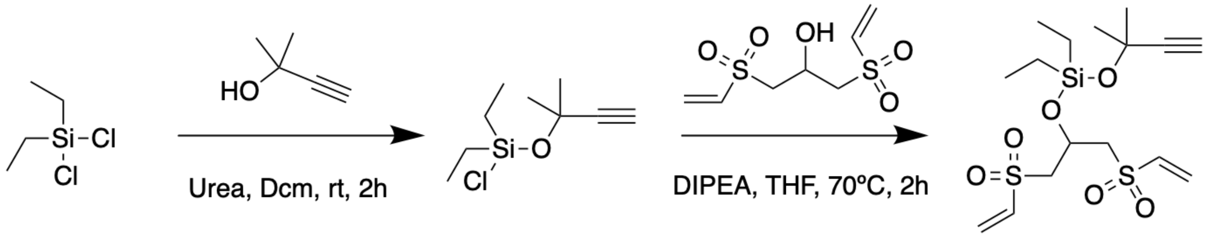

BVSS-Et-S1 **BVSS-Et**

Chlorodiethyl((2-methylbut-3-yn-2-yl)oxy)silane (BVSS-Et-S1). Urea (3.6 g, 60 mmol) and dichlorodiethylsilane (7.9 g, 50 mmol) were stirred in dichloromethane (10 mL) at rt for 15 min, and then 2-methylbut-3-yn-2-ol (4.2 g, 50 mmol) was added dropwise. The mixture was stirred at rt for 2h and then filtered. The filtrate was concentrated (Caution: the product is volatile) and then distilled under vacuum to give a colorless oil. 6.8 g, 68% in yield. ^{1}H NMR (400 MHz, $CDCl_3$) δ 2.47 (s, 1H), 1.57 (s, 6H), 1.03 (m, 6H), 0.93 (m, 4H). ^{13}C NMR (101 MHz, $CDCl_3$) δ 87.96, 77.55, 77.23, 76.91, 71.73, 68.39, 32.66, 9.92, 6.62.

((1,3-bis(vinylsulfonyl)propan-2-yl)oxy)diethyl((2-methylbut-3-yn-2-yl)oxy)silane (BVSS-Et). Chlorodiethyl((2-methylbut-3-yn-2-yl)oxy)silane (1.0 g, 5.0 mmol) and 1,3-bis(vinylsulfonyl)propan-2-ol (1.2 g, 5.0 mmol) were mixed in THF (10 mL), and then TEA (0.60 g, 5.5 mmol) was added. The mixture was microwaved at 70 ºC for 2h. The solid was filtered, the filtrate was concentrated and then purified by flash chromatography on silica eluted with 0-70% ethyl acetate in hexanes to give the product as colorless oil. 0.30g, 15 % in yield. ^{1}H NMR (400 MHz, $CDCl_3$) δ 6.73 (dd, J = 16.6, 9.9 Hz, 2H), 6.43 (d, J = 16.7 Hz, 2H), 6.13 (d, J = 9.9 Hz, 2H), 4.89 (ddd, J = 11.1, 6.2, 4.9 Hz, 1H), 3.68 (dd, J = 14.7, 6.3 Hz, 2H), 3.48 (dd, J = 14.7, 4.8 Hz, 2H), 2.49 (s, 1H), 1.53 (s, 6H), 1.00 (t, J = 7.9 Hz, 6H), 0.75 (d, J = 8.3 Hz, 4H). ^{13}C NMR (101 MHz, $CDCl_3$) δ 137.88, 129.88, 88.27, 71.81, 67.06, 62.82, 59.12, 32.91, 6.64, 6.22. MS: $C_{16}H_{28}O_6S_2Si$ Calculated for 408.60, found: 431.0 (M+Na).

((1,3-bis(vinylsulfonyl)propan-2-yl)oxy)((2-methylbut-3-yn-2-yl)oxy)diphenylsilane (BVSS-Ph)

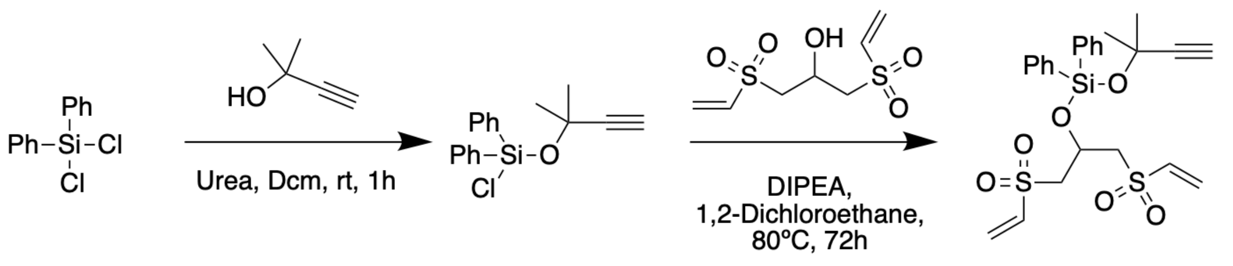

BVSS-Ph-S1 **BVSS-Ph**

Chloro((2-methylbut-3-yn-2-yl)oxy)diphenylsilane (BVSS-Ph-S1). Urea (7.2 g, 1.5 Eq, 0.12 mol) and dichlorodiphenylsilane (20 g, 1 Eq, 0.080 mol) were stirred at rt for 15 min, and then 2-methylbut-3-yn-2-ol (9.4 g, 1.4 Eq, 0.11 mol) was added dropwise, the mixture was stirred for 2 h at room temperature, and then filtered carefully by excluding moisture, because urea hydrochloride is very hygroscopic. The clear liquid was separated and concentrated to give a colorless oil. 22 g. 91 % in yield, which was used without further purification. ^{1}H NMR (400 MHz, cdcl$_3$) δ 7.76 (d, *J* = 7.9 Hz, 4H), 7.45 (dd, *J* = 17.4, 7.2 Hz, 6H), 2.35 (s, 1H), 1.67 (s, 6H). ^{13}C NMR (101 MHz, cdcl$_3$) δ 134.70, 130.94, 128.06, 87.29, 72.42, 69.77, 32.64. Chemical Formula: $C_{17}H_{17}ClOSi$ calculated 300.07, found 323.0 (M+Na).

((1,3-bis(vinylsulfonyl)propan-2-yl)oxy)((2-methylbut-3-yn-2-yl)oxy)diphenylsilane (BVSS-Ph). Chloro((2-methylbut-3-yn-2-yl)oxy)diphenylsilane (1.3 g, 4.2 mmol) and 1,3-bis(vinylsulfonyl) propan-2-ol (1.0 g, 4.2 mmol) were mixed in 1,2-dicloroethane (10 mL) at room temperature, and then DIPEA (1.0 mL, 6.0 mmol) was added. The mixture was heated under microwave at 80 °C for 72 h, and then concentrated. The crude product was purified by flash chromatography on a silica column eluted with 0-70 % ethyl acetate in hexanes to afford yellowish sticky oily product which solidified upon standing. 0.83 g, 40 % in yield. ^{1}H NMR (400 MHz, cdcl$_3$) δ 7.69 (d, *J* = 7.6 Hz, 2H), 7.48 (t, *J* = 7.2 Hz, 1H), 7.40 (t, *J* = 7.4 Hz, 2H), 6.48 (dd, *J* = 16.6, 9.8 Hz, 1H), 6.29 (d, *J* = 16.6 Hz, 1H), 5.93 (d, *J* = 9.8 Hz, 1H), 4.79 (p, *J* = 5.4 Hz, 1H), 3.67 (dd, *J* = 14.8, 6.6 Hz, 1H), 3.48 (dd, *J* = 14.8, 4.5 Hz, 1H), 1.53 (s, 3H). ^{13}C NMR (101 MHz, cdcl$_3$) δ 137.27, 135.51, 132.15, 131.15, 130.17, 128.20, 88.08, 72.18, 68.76, 63.88, 58.70, 32.70. Chemical Formula: $C_{24}H_{28}O_6S_2Si$ calculated: 504.11, found: 527.00 (M+Na).

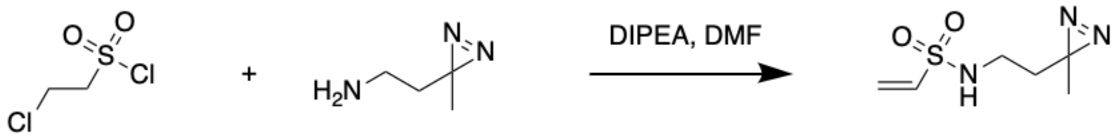

VSD

***N*-(2-(3-methyl-3*H*-diazirin-3-yl)ethyl)ethenesulfonamide (VSD)**. To a dry round bottom flask covered from light was added 2-chloroethylsulfonyl chloride (493.3 mg, 3.0 mmol) that was dissolved in DMF (5mL) followed by DIPEA (1 mL, 6.1 mmol) under N_2 gas. Next, 3-methyl-3A-diazirine-3-ethanamine (200 mg, 2.0 mmol) was added dropwise and the reaction mixture was stirred at RT for 18 hours. The reaction mixture was concentrated *in vacuo* and purified on an ISCO Combiflash system using 45-65% ethyl acetate/hexanes and product was dried in vacuo to afford a clear oil. 0.11 g (0.59 mmol), 29% in yield. ^{1}H NMR (400 MHz, MeOD) δ 6.62 (dd, *J*1 = 8 Hz, *J*2 = 4 Hz, 1H), 6.14 (d, 1H), 5.97 (d, 1H), 2.88 (t, J = 8 Hz, 2H), 1.55 (t, J = 8 Hz, 2H), 1.03 (s, 3H). ^{13}C NMR (101 MHz, MeOD) δ 137.55, 126.60, 38.98, 36.02, 25.12, 19.77.

D. Compound Characterization Data

NMR Data

4-isocyanatobut-1-yne

^{1}H NMR

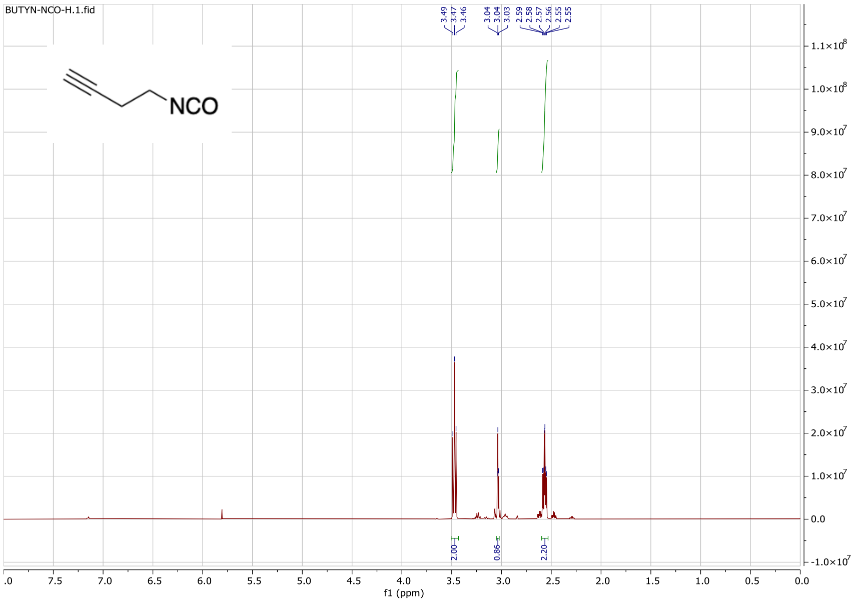

^{13}C NMR

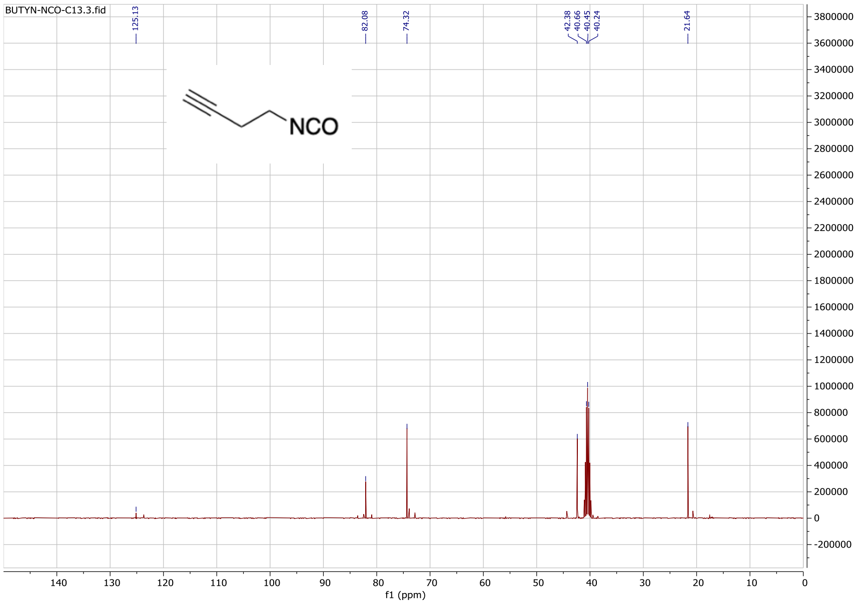

ALKYNE-BVSC

^{1}H NMR

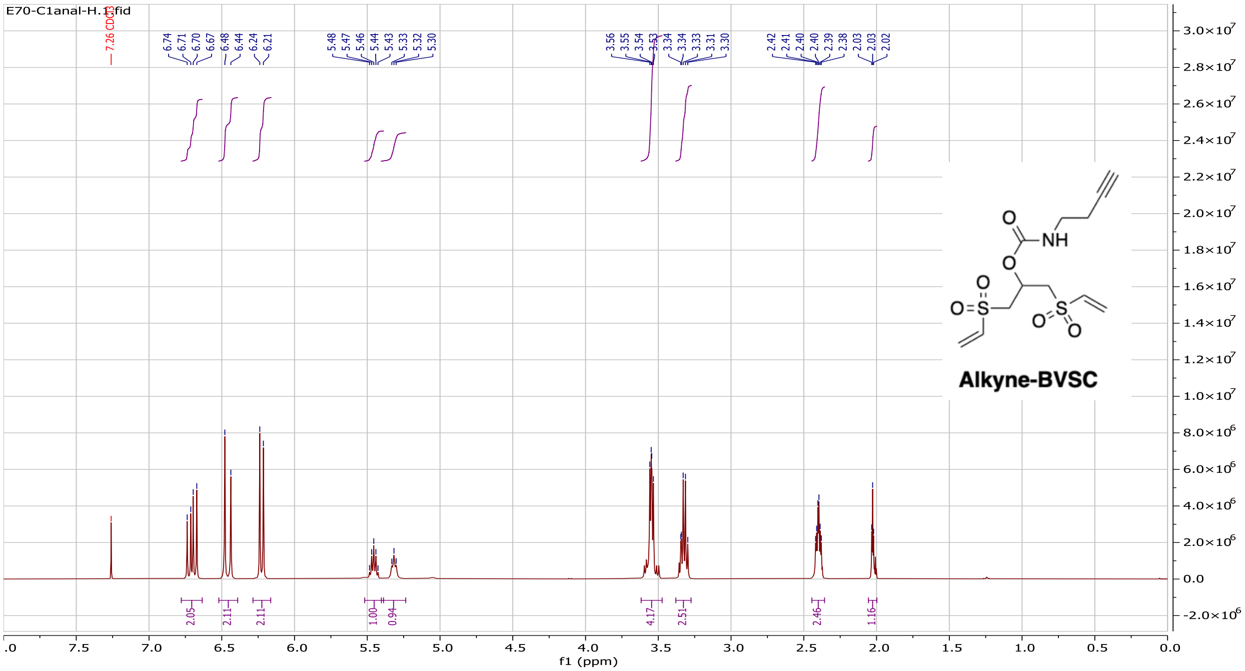

^{13}C NMR

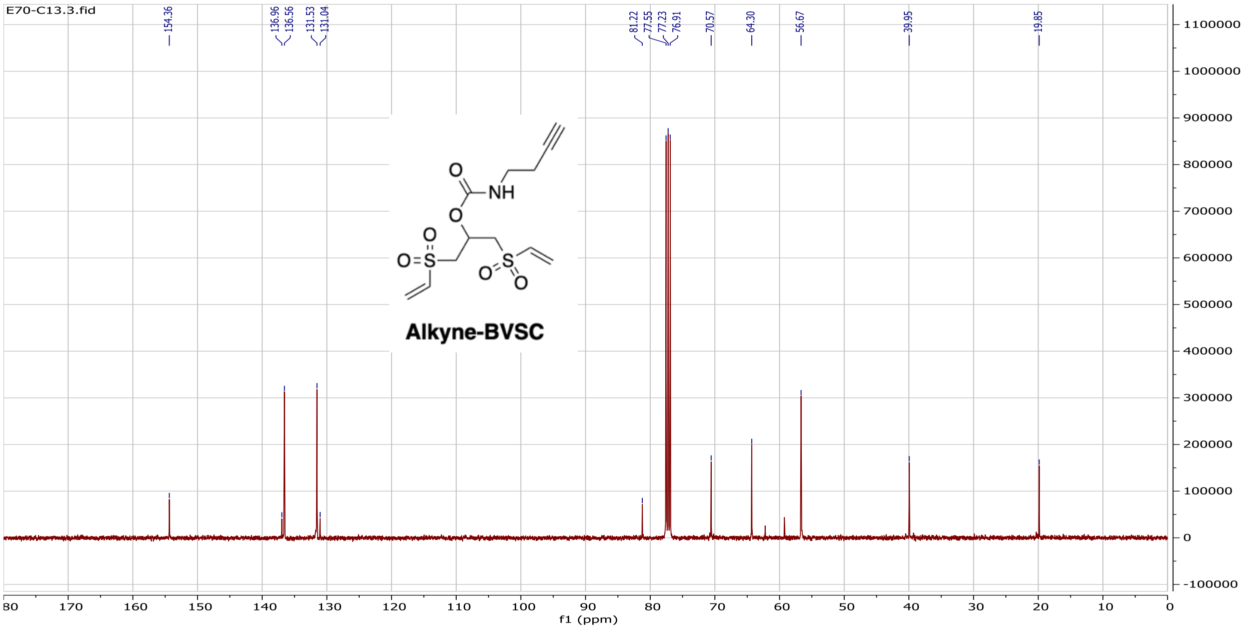

BVSS-Me-S1

^{1}H NMR

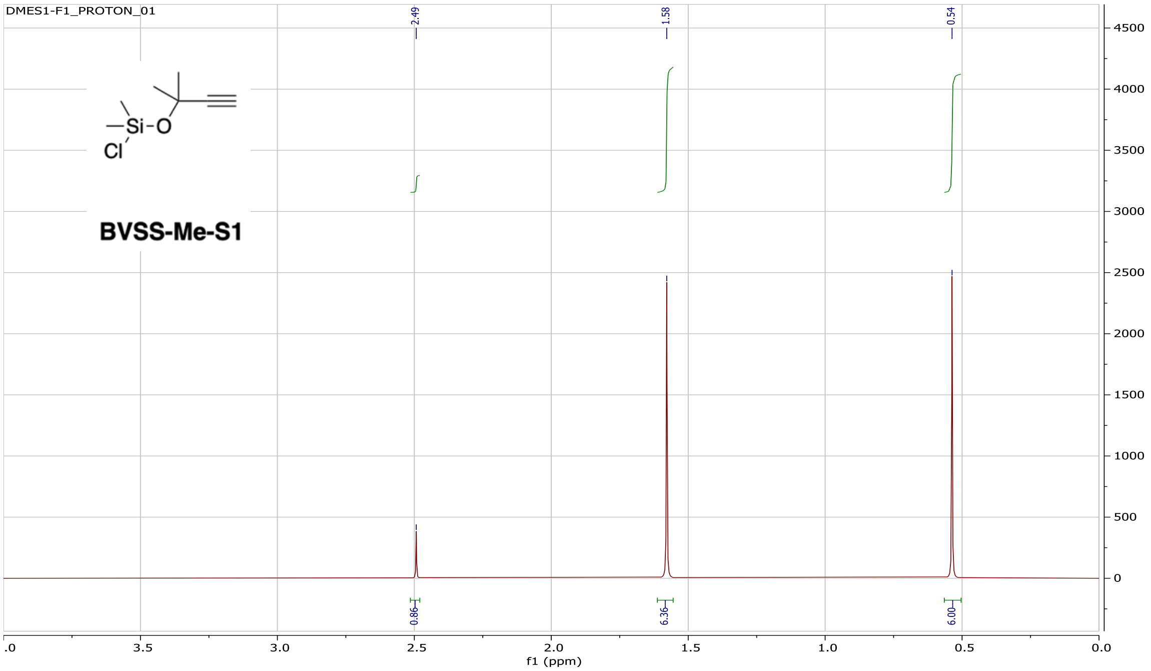

^{13}C NMR

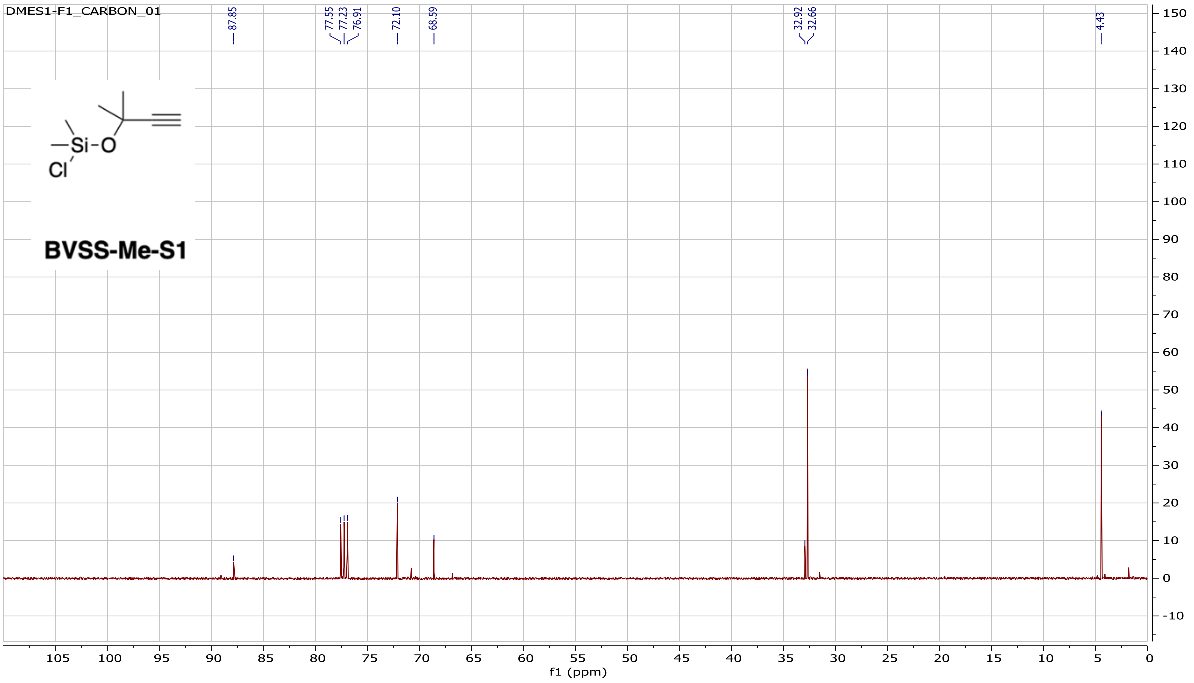

BVSS-Me

^{1}H NMR

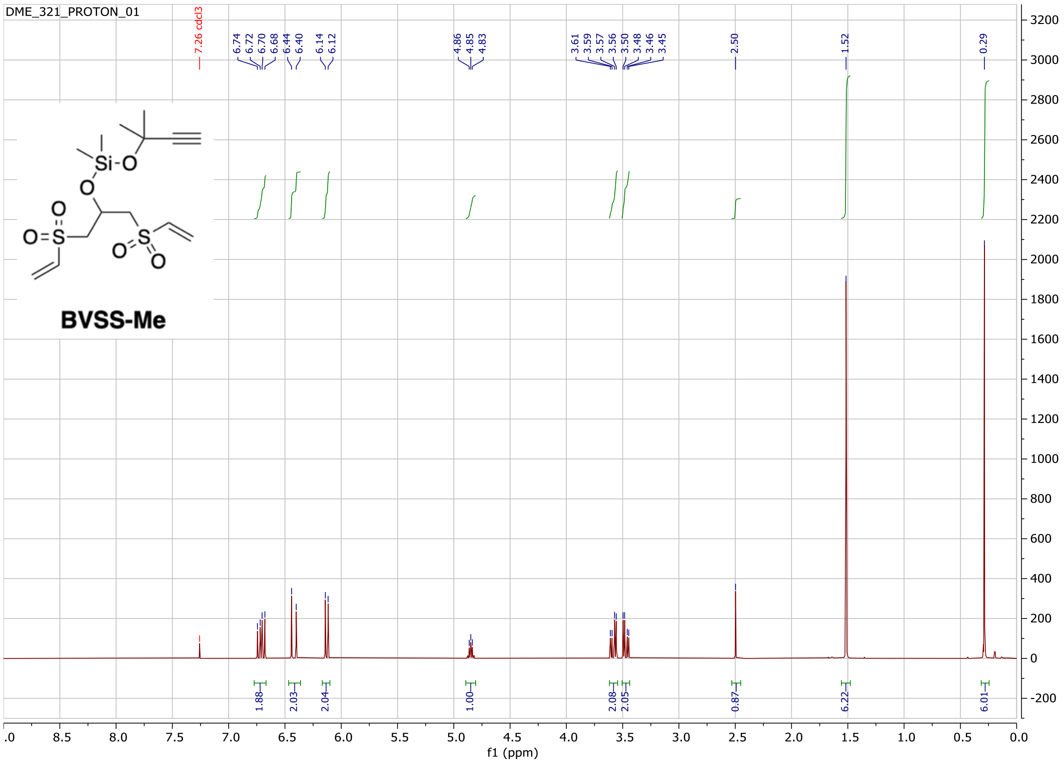

^{13}C NMR

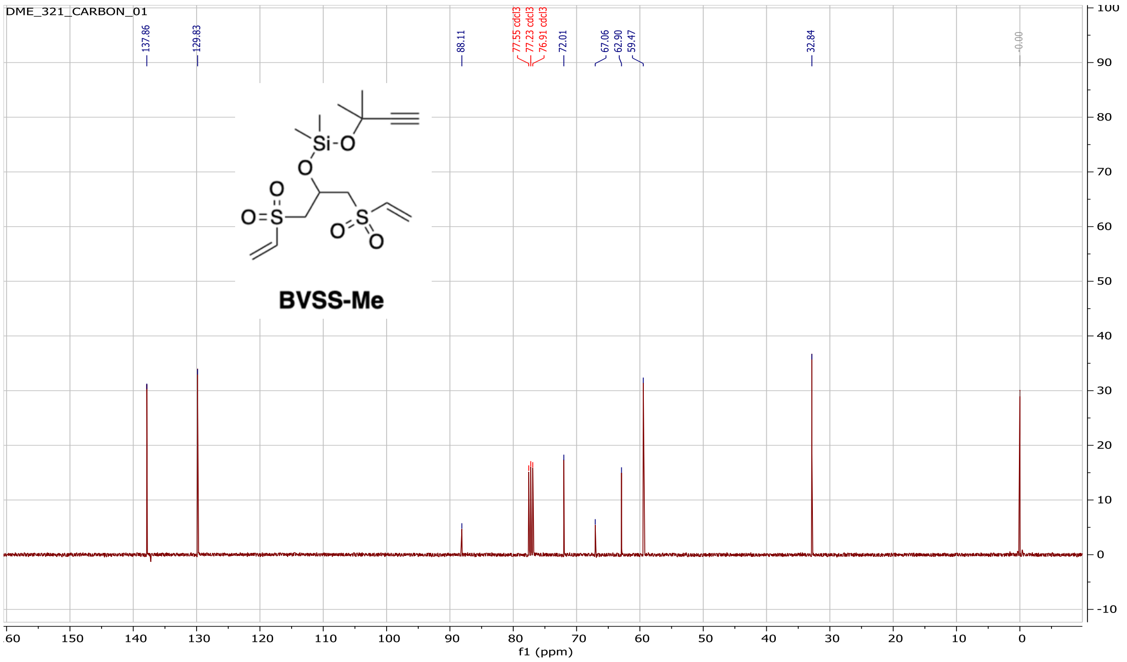

BVSS-Et-S1

^{1}H NMR

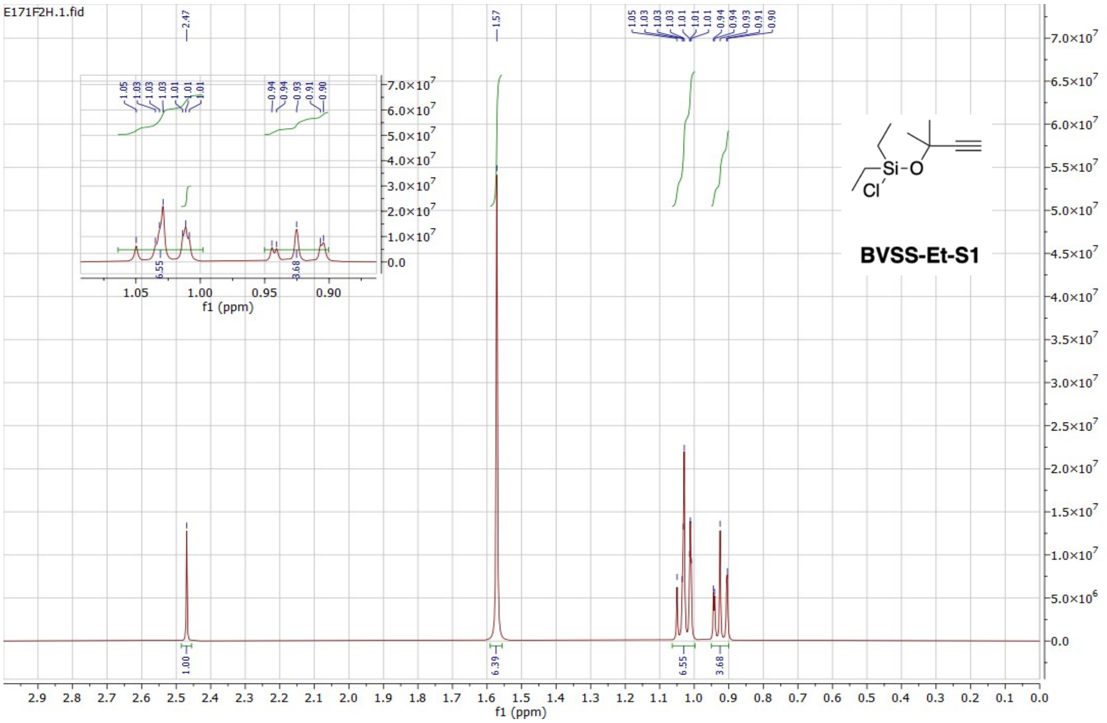

^{13}C NMR

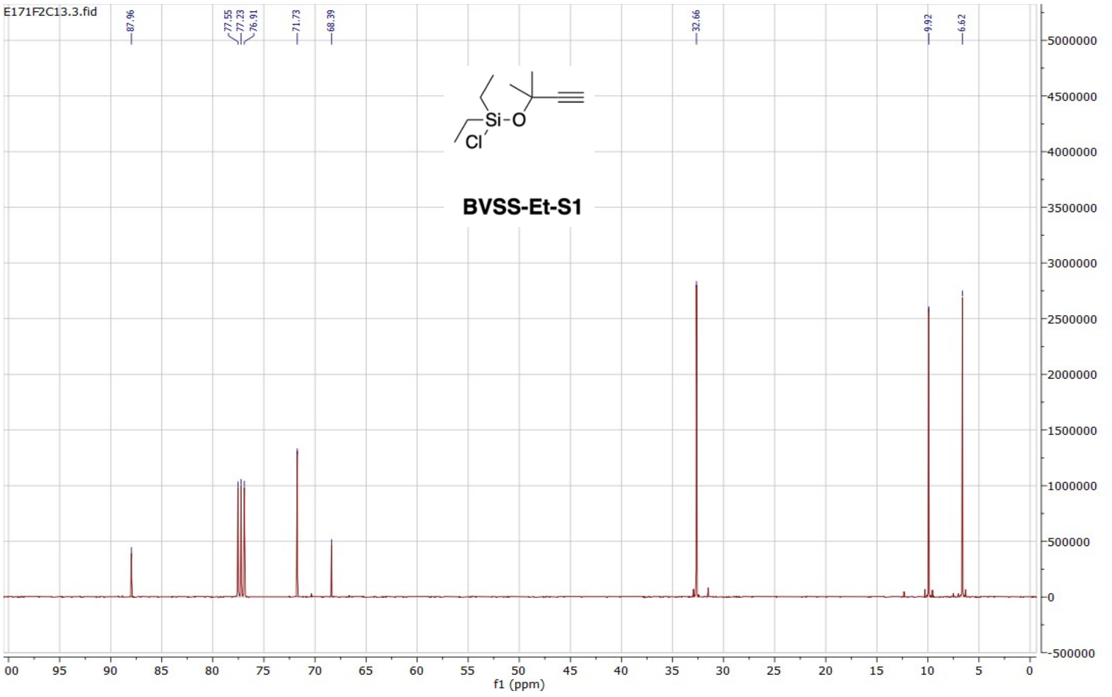

BVSS-Et

^{1}H NMR

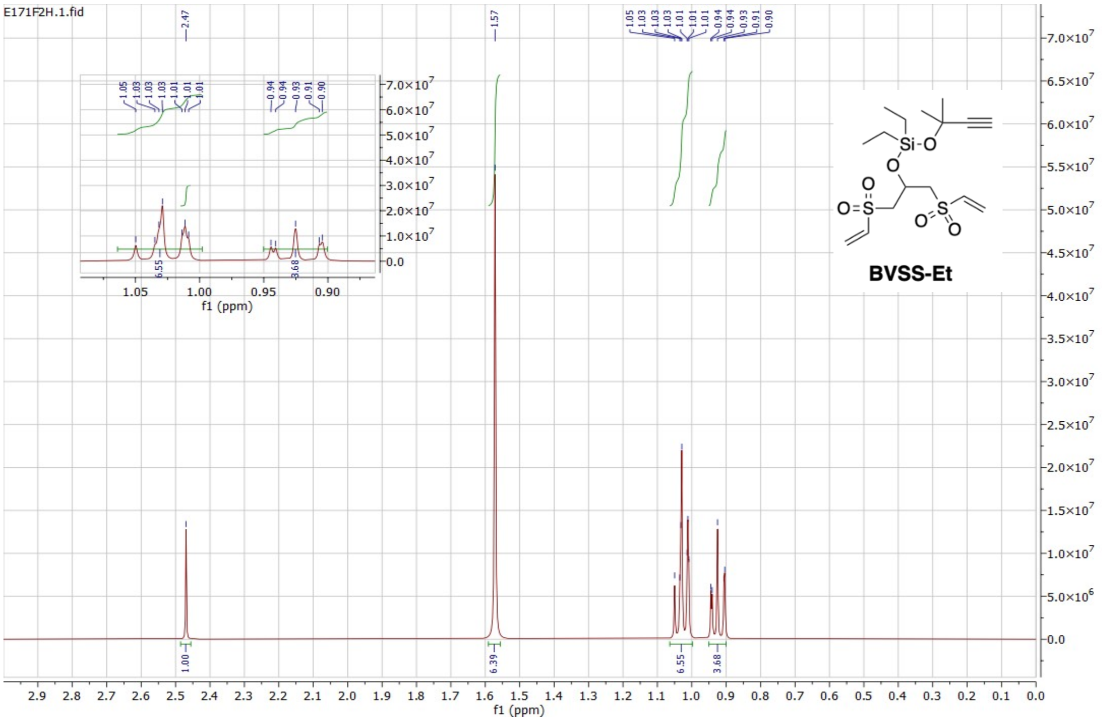

^{13}C NMR

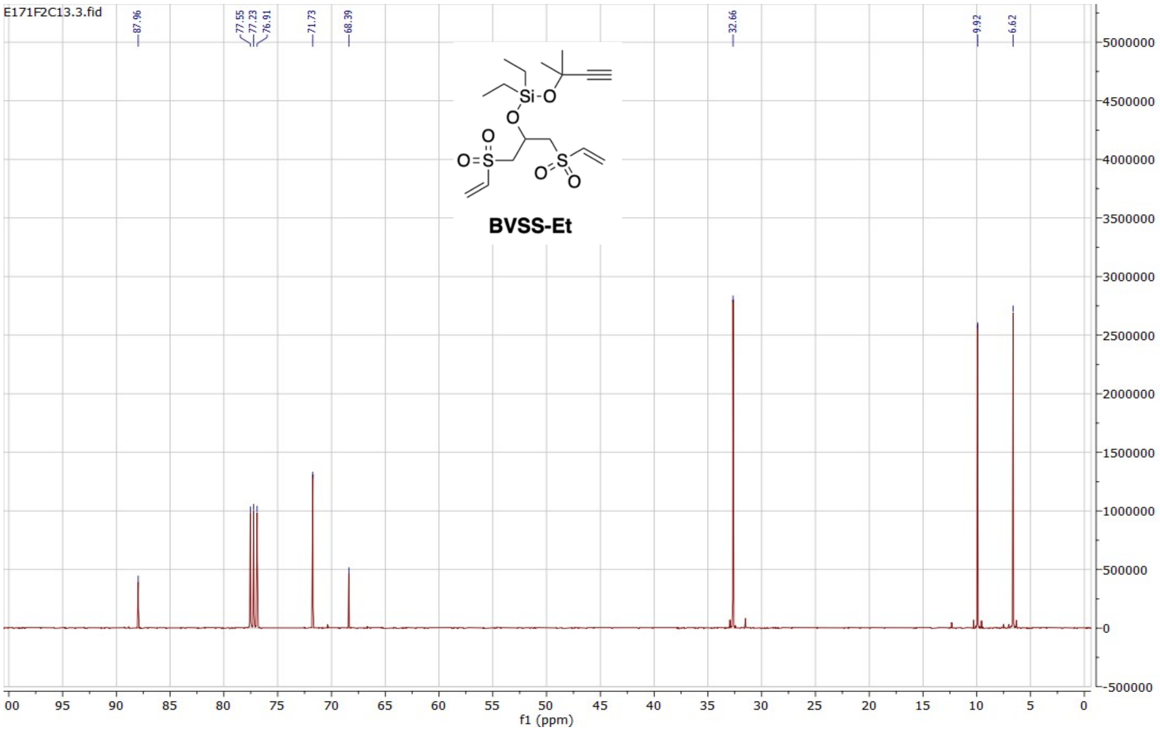

BVSS-Ph-S1

^{1}H NMR

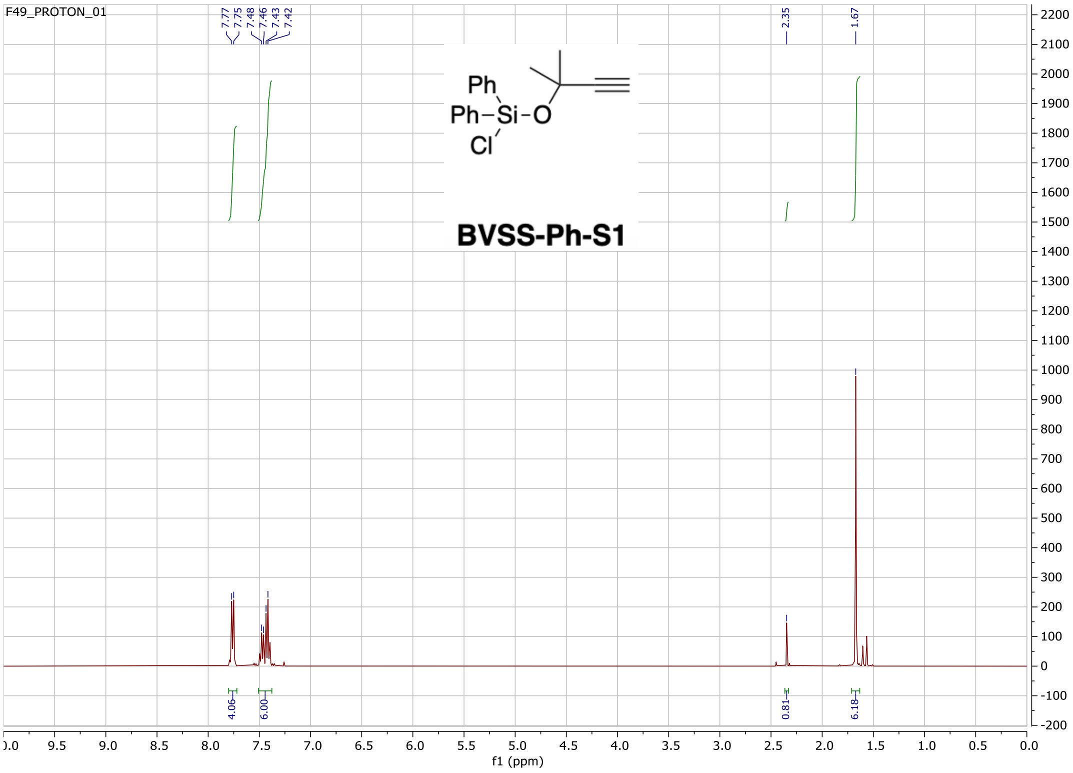

^{13}C NMR

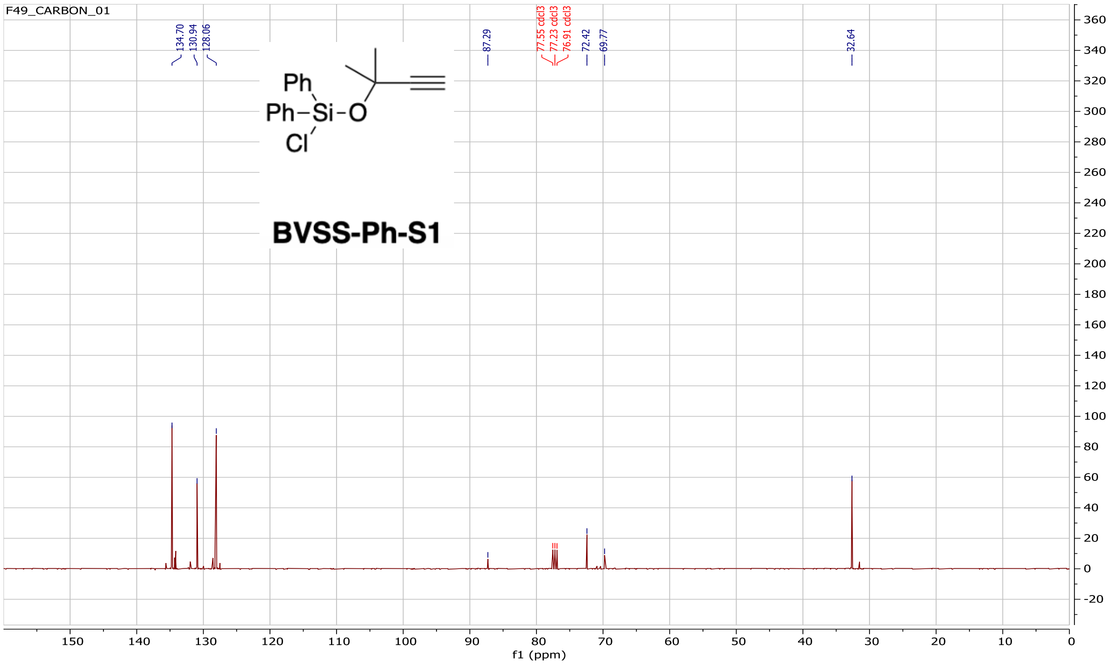

BVSS-Ph

^{1}H NMR

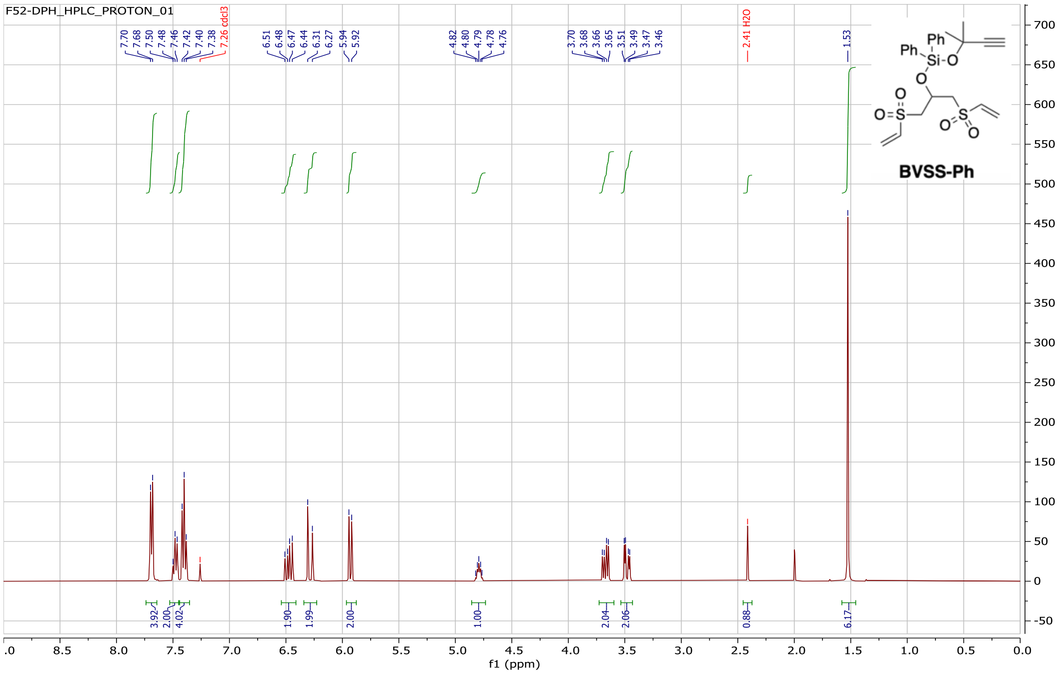

^{13}C NMR

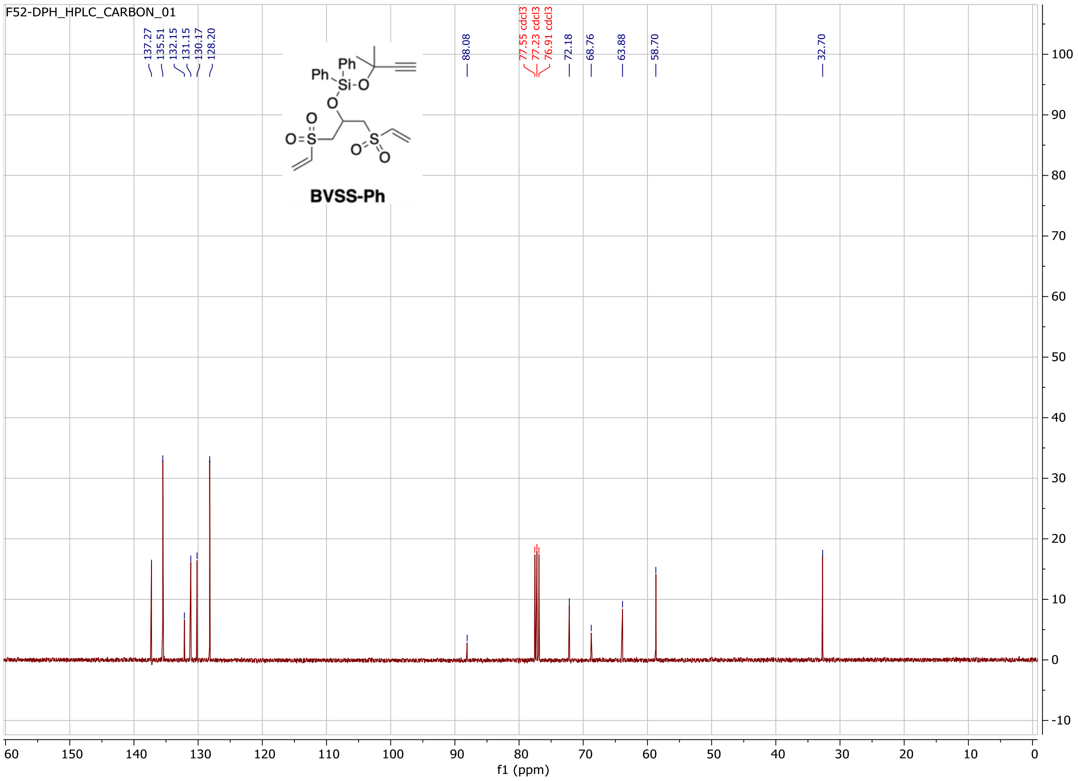

^{1}H NMR

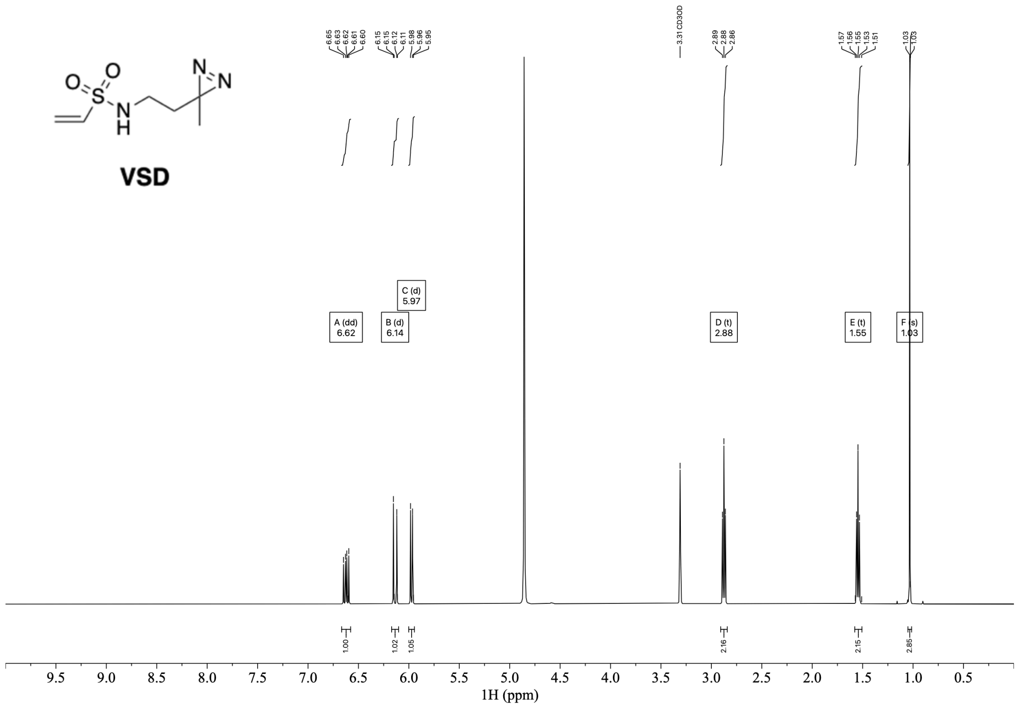

^{13}C NMR

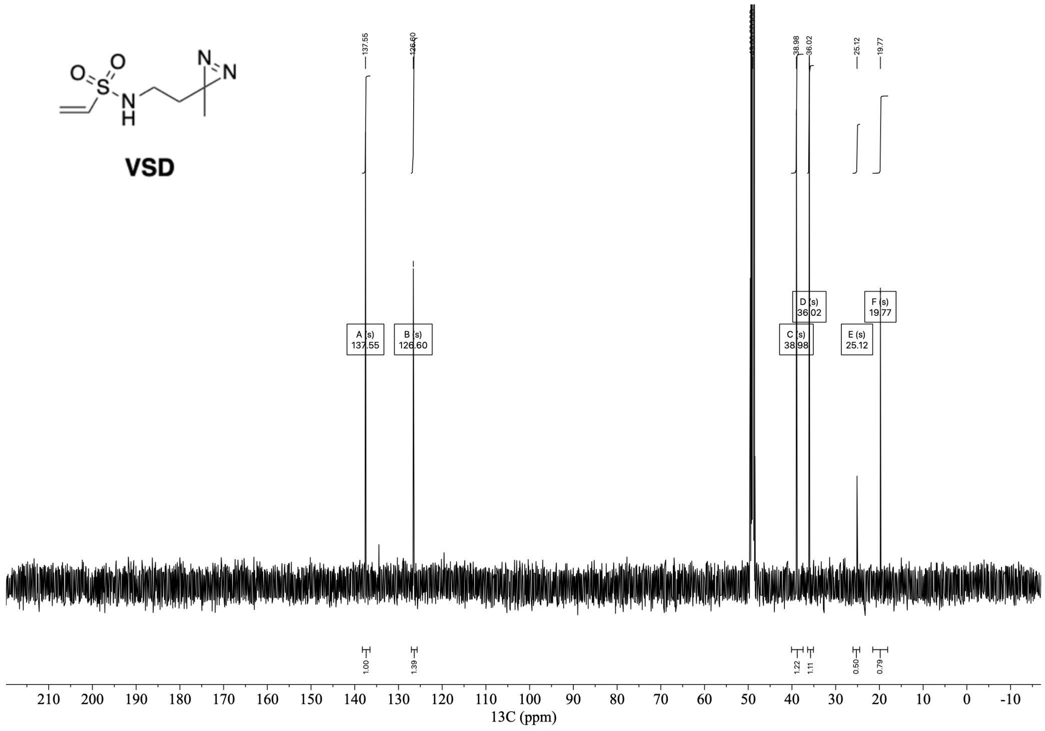

References

1. Hahm, H.S., et al., *Global targeting of functional tyrosines using sulfur-triazole exchange chemistry.* Nat Chem Biol, 2020. **16**(2): p. 150-159.
2. Negi, H., et al., *An engineered cell line with a hRpn1-attached handle to isolate proteasomes.* J Biol Chem, 2023. **299**(8): p. 104948.
3. Reimund, M., et al., *Structure of apolipoprotein B100 bound to the low-density lipoprotein receptor.* Nature, 2024.
4. Rappsilber, J., M. Mann, and Y. Ishihama, *Protocol for micro-purification, enrichment, pre-fractionation and storage of peptides for proteomics using StageTips.* Nat Protoc, 2007. **2**(8): p. 1896-906.
5. Zhu, Y., et al., *Cross-link assisted spatial proteomics to map sub-organelle proteomes and membrane protein topologies.* Nat Commun, 2024. **15**(1): p. 3290.
6. Colin W. Combe, M.G., Lars Kolbowski, Lutz Fischer, Juri Rappsilber, *xiVIEW: Visualisation of Crosslinking Mass Spectrometry Data.* Journal of Molecular Biology, 2024.
7. Chang, H.Y., et al., *Crystal-C: A Computational Tool for Refinement of Open Search Results.* J Proteome Res, 2020. **19**(6): p. 2511-2515.
8. da Veiga Leprevost, F., et al., *Philosopher: a versatile toolkit for shotgun proteomics data analysis.* Nat Methods, 2020. **17**(9): p. 869-870.
9. Demichev, V., et al., *DIA-NN: neural networks and interference correction enable deep proteome coverage in high throughput.* Nat Methods, 2020. **17**(1): p. 41-44.
10. Kong, A.T., et al., *MSFragger: ultrafast and comprehensive peptide identification in mass spectrometry-based proteomics.* Nat Methods, 2017. **14**(5): p. 513-520.
11. Yu, F., et al., *Identification of modified peptides using localization-aware open search.* Nat Commun, 2020. **11**(1): p. 4065.
